# Identification of *Populus* Small RNAs Responsive to Mutualistic Interactions With Mycorrhizal Fungi, *Laccaria bicolor* and *Rhizophagus irregularis*

**DOI:** 10.3389/fmicb.2019.00515

**Published:** 2019-03-18

**Authors:** Ritesh Mewalal, Hengfu Yin, Rongbin Hu, Sara Jawdy, Patrice Vion, Gerald A. Tuskan, François Le Tacon, Jessy L. Labbé, Xiaohan Yang

**Affiliations:** ^1^Biosciences Division, Oak Ridge National Laboratory, Oak Ridge, TN, United States; ^2^State Key Laboratory of Tree Genetics and Breeding, Research Institute of Subtropical Forestry, Chinese Academy of Forestry, Fuyang, China; ^3^INRA, UMR 1136 INRA-University of Lorraine, Interactions Arbres/Microorganismes, Laboratory of Excellence ARBRE, INRA-Nancy, VandIJuvre-lès-Nancy, France

**Keywords:** arbuscular mycorrhizal fungus, ectomycorrhizal fungus, *Laccaria*, microRNA, mutualistic symbiosis, *Populus*, *Rhizophagus*, small RNA

## Abstract

Ecto- and endo-mycorrhizal colonization of *Populus* roots have a positive impact on the overall tree health and growth. A complete molecular understanding of these interactions will have important implications for increasing agricultural or forestry sustainability using plant:microbe-based strategies. These beneficial associations entail extensive morphological changes orchestrated by the genetic reprogramming in both organisms. In this study, we performed a comparative analysis of two *Populus* species (*Populus deltoides* and *P. trichocarpa*) that were colonized by either an arbuscular mycorrhizal fungus (AmF), *Rhizophagus irregularis* or an ectomycorrhizal fungus (EmF), *Laccaria bicolor*, to describe the small RNA (sRNA) landscape including small open reading frames (sORFs) and micro RNAs (miRNAs) involved in these mutualistic interactions. We identified differential expression of sRNAs that were, to a large extent, (1) within the genomic regions lacking annotated genes in the *Populus* genome and (2) distinct for each fungal interaction. These sRNAs may be a source of novel sORFs within a genome, and in this regard, we identified potential sORFs encoded by the sRNAs. We predicted a higher number of differentially-expressed miRNAs in *P. trichocarpa* (4 times more) than in *P. deltoides* (conserved and novel). In addition, 44 miRNAs were common in *P. trichocarpa* between the EmF and AmF treatments, and only 4 miRNAs were common in *P. deltoides* between the treatments. Root colonization by either fungus was more effective in *P. trichocarpa* than in *P. deltoides*, thus the relatively few differentially-expressed miRNAs predicted in *P. deltoides* might reflect the extent of the symbiosis. Finally, we predicted several genes targets for the plant miRNAs identified here, including potential fungal gene targets. Our findings shed light on additional molecular tiers with a role in *Populus*-fungal mutualistic associations and provides a set of potential molecular targets for future enhancement.

## Introduction

Mycorrhizal fungi are a diverse group of beneficial organisms that colonize the roots of more than 90% of higher plant species. Typically, two main types of fungi are described: (1) arbuscular mycorrhizal fungi (AmF), developing inside the root cell walls, e.g., *Rhizophagus irregularis*, and (2) ectomycorrhizal fungi (EmF), colonizing the intercellular spaces of the roots, e.g., *Laccaria bicolor* (Bonfante and Genre, 2010). These fungi play an important role in the maintenance of the plant health and growth by promoting water cycling, nutrient exchange and enhanced tolerance/resistance to biotic and abiotic stresses, while in exchange, the fungi receive plant-fixed carbon (Smith and Read, 2008; Bonfante and Genre, 2010). Several studies have shown that field application of mycorrhizal fungi improves the overall productivity of a number of crops including cereals, legumes, fruits and trees (Abbott and Robson, 1977; Brundrett et al., 1996; Al-Karaki et al., 2004; Powell, 2018). To address the challenge to food and energy security caused by increases in the global population, and decreases in agricultural and forest land, it is important to gain a deeper understanding of the molecular mechanism underlying beneficial symbiosis between plant and fungi to effectively design and develop plant:microbe-based strategies to enhance forestry and agriculture health and sustainability (Martin et al., 2017).

Much progress has been made in understanding the establishment and maintenance of these mutualistic associations (Bonfante and Genre, 2010; Plett and Martin, 2011). Many studies support the hypothesis that fungi-derived protein signals or effectors facilitate and/or maintain the symbiotic interactions (Daguerre et al., 2017). For example, the genome of *L. bicolor* encodes a large number of mycorrhizal-induced small secreted proteins (MiSSPs), many of which are expressed and accumulated in the fungal hyphae during colonization (Martin et al., 2008). Plett et al. (2011) reported that the effector protein of *L. bicolor*, MiSSP7 could enter the nucleus of *Populus* roots cells to affect transcription and promote symbiosis. MiSSP7 protects the *Populus* jasmonate zim-domain protein 6 (PtJAZ6), which is a negative regulator of jasmonic acid (JA)-induced gene regulation in *Populus*, and promotes symbiosis by blocking the action of jasmonic acid (Plett et al., 2014). Similarly, a secreted protein of *R. irregularis*, SP7, interacts with ERF19, a pathogenesis-related transcription factor that counteracts the plant immune response and facilitates root colonization by this AmF (Kloppholz et al., 2011).

On the plant side, colonization by fungi requires that the plant distinguish between beneficial and pathogenic fungi (el Zahar Haichar et al., 2014). Plant root exudates have been shown to (1) mediate chemotaxis signaling that facilitate the colonization of roots by flagellated bacteria (Scharf et al., 2016) and (2) contain secondary metabolites, strigolactones, which promote hyphal branching and stimulates fungal spore germination (Akiyama et al., 2005). Moreover, transcriptome analysis of *Populus trichocarpa* roots colonized by *L. bicolor* has revealed 417 putative plant-encoded small secreted proteins (SSPs) with 39% of them appearing to be specific to *Populus*, where several of these SSPs were able to enter the hyphae and affect the growth of *L. bicolor* (Plett et al., 2017). These studies suggest that the genetic contributions from a plant in mutualistic association may be more complex than our current understanding and may involve several levels of regulation. It is unclear if this molecular toolbox for symbiosis, i.e., set of molecular determinants (e.g., protein-encoding genes, non-coding RNAs) are shared across different plant species when colonized by the same fungi or alternatively, the same plant species colonized by different types of symbiotic fungi.

In recent years, the role of small non-coding RNAs (sRNAs), broadly defined as regulatory RNA molecules ranging in size from 20 to 300 nucleotides (Großhans and Filipowicz, 2008), have become apparent in biotic stresses and regulation of plant development and physiology (Mallory and Vaucheret, 2006; Großhans and Filipowicz, 2008; Ruiz-Ferrer and Voinnet, 2009; Chen, 2012; Zhang and Chen, 2013). These regulatory RNA molecules include small interfering RNAs (siRNAs), microRNAs (miRNAs), piRNAs (Piwi-associated RNAs), and long non-coding RNAs (lncRNAs), which may originate from intergenic, intronic, or antisense transcripts. Several detailed reviews of molecular mechanism of these different population of non-coding RNAs (ncRNAs) were published recently (Ruiz-Ferrer and Voinnet, 2009; Chekanova, 2015; Mohanta and Bae, 2015; Huang et al., 2016). miRNAs, typically between 20 and 25 nucleotides, are processed from single-stranded RNA to form imperfect base-paired hairpin secondary structures, and generally negatively regulate their targets including mRNAs (Chen, 2008; Lanet et al., 2009) and ncRNAs such as TAS RNAs (Vaucheret, 2006). Many lines of evidence now confirm that miRNAs are necessary for plant association with AmF (Branscheid et al., 2010; Devers et al., 2011; Lauressergues et al., 2012; Etemadi et al., 2014). For example, colonization of *Medicago truncatula* roots by *R. irregularis* was reduced when miR171h was overexpressed (Lauressergues et al., 2012; Hofferek et al., 2014). In plants, sRNAs have also been shown to move between cells via the plasmodesmata, and transverse long distance via the phloem (Brosnan and Voinnet, 2011). Furthermore, sRNA can transverse into interacting fungi through the haustorium, to trigger gene silencing (Molnar et al., 2011; Huang et al., 2016). Consequently, sRNAs are now viewed as an important part of the genetic response network for evolutionary adaption (Amaral et al., 2013).

Studies have reported that sRNAs can also have protein-coding potential and encode small open reading frames (sORFs) which typically contain between 30 and 100 amino acids (Ulveling et al., 2011; Andrews and Rothnagel, 2014; Li et al., 2014; Ruiz-Orera et al., 2014; Kumari and Sampath, 2015). The portfolio of organisms with functional sORFs include bacteria, yeast, *Drosophila*, plants and human (Oyama et al., 2004; Kastenmayer et al., 2006; Hanada et al., 2007; Ladoukakis et al., 2011; Yang et al., 2011; Aspden et al., 2014; Ruiz-Orera et al., 2014; Shell et al., 2015; Ma et al., 2016). sORFs are partially classified based on their genomic position, e.g., upstream or downstream annotated genes, within annotated genes but out-of-frame, antisense to annotated genes, within novel transcripts and within ncRNAs. The biological significance for these types of sORFs varies. For example, the tarsel-less (tal) gene from *Drosophila* was classified as ncRNA but the transcript does encode four sORFs (11–32 AAs) that affect *Drosophila* gene expression and development (Galindo et al., 2007). The intergenic region of *Arabidopsis* has been predicted to encode approximately 33,809 sORFs (Hanada et al., 2007). Additionally, Castellana et al. (2008) identified ∼5,000 small peptides in *Arabidopsis* in a proteomic study, several of which were novel and/or identified in the aforementioned study by Hanada et al. (2007). Despite an unclear molecular mechanism, Hanada et al. (2013) showed varying morphological changes caused by overexpression of sORFs in *Arabidopsis*. Thus, it is plausible that sORFs derived from ncRNAs may provide another level of developmental regulation in mutualistic symbiosis.

In this study, we (1) identify and compare the *Populus*-derived sRNAs response to two different mutualistic symbionts (i.e., *R. irregularis* and *L. bicolor*) and (2) predict and compare the encoded sORFs, as well as their miRNAs and putative gene targets potentially involved in formation and maintenance of the beneficial associations. To achieve this goal, we profiled the sRNA response of two *Populus* species (*P. deltoides* and *P. trichocarpa*) that were each colonized by either of the two different fungi (Tagu et al., 2001; Labbé et al., 2011). Our study provides insight into the common and differential regulation that may be involved in both the endo- and ecto-mycorrhizal associations among members of the genus *Populus*.

## Materials and Methods

### Plant Material and Inoculation

Internode cutting of *P. trichocarpa* (genotype 101–74) and *P. deltoides* (genotype 73028-62) of the 54B F1 pedigree (Jorge et al., 2005) parental lines were rooted and individually inoculated with a 1/9 (v/v) mixture of *L. bicolor* inoculum and calcined clay (Oil Dri US Special, Damolin, Denmark). Eight replicates per genotype and treatment were carried out. *L. bicolor* S238N inoculum was produced by growing mycelium on an autoclaved peat-vermiculite nutrient mixture in 1-L glass jars for 2 months in the dark at 25°C and kept at 4°C before use (Duponnois and Garbaye, 1991). *R. irregularis* inoculum consisted of spores (200 spores per litter of calcinate clay). Inoculated cuttings were grown for 3 months post-inoculation in a greenhouse at 15–28°C, 12 h photoperiod at INRA-Nancy, France during summer and then harvested. One portion of the sampled root (about 25%) was used to assess successful root colonization, which was confirmed under a stereomicroscope by counting ectomycorrhizas for *L. bicolor* and for *R. irregularis* by counting arbuscules and measuring how much of the root length is colonized after Trypan blue staining procedure as described by Koske and Gemma (1989). The remaining lateral root samples (about 75%) were immediately frozen in liquid nitrogen at -80°C for RNA isolation.

### RNA Isolation and Sequencing

Total RNA was extracted from the root system of three of the eight replicates per genotype per treatment by combining a CTAB extraction method and the Spectrum^TM^ Total Plant RNA extraction kit (Sigma) as reported in Bryan et al. (2016). Prior to synthesizing sequencing libraries, RNA templates were quantified using a NanoDrop Spectrophotometer (Thermo Scientific) and RNA quality was determined using BioRad Experion^TM^. Samples with a 260/280 reading between 2.0 and 2.2 and a RIN > 7 were determined to be of high enough quality and the high-quality RNA samples were used for sRNA-sequencing library preparation with an Illumina TruSeq small RNA sample prep kit (Illumina, CA, United States). As specified in the Illumina protocol, small RNA fragments were selected after PCR amplification by gel purification to capture RNA approximately 20–170 bp in length. The Illumina TruSeq small RNA library prep workflow is considered to be stranded because adapters are ligated directionally, facilitating maintenance of strand information during analysis. A total of 18 sRNA-sequencing libraries (three biological replicates of the control *P. deltoides* and control *P. trichocarpa*, and from the treatments, EmF-*P. deltoides*, EmF- *P. trichocarpa*, and AmF-*P. deltoides* and AmF-*P. trichocarpa*) were constructed. The libraries were sequenced on an Illumina MiSeq system (Illumina, CA, United States) to generate singe-end reads (1 × 150 bp).

### sRNA-Seq Data Analysis

In this study, small RNAs are defined as the RNA molecules with size ranging from 20 to 300 nucleotides, consistent with the definition by Großhans and Filipowicz (2008). The raw RNA-Seq data in Fastq format were quality checked and trimmed for low-quality regions and adaptor sequences using Trimmomatic v0.35 (Bolger et al., 2014), parameters of “2:30:10 LEADING:3 TRAILING:3 SLIDINGWINDOW:4:15 MINLEN:20.” The trimmed sequencing reads from *P. trichocarpa* and *P. deltoides* were mapped to the *P. trichocarpa* genome v3.0 and *P. deltoides* WV94 v2.1^1^, respectively, using TopHat2 (Kim et al., 2013). Novel genes were assembled and transcript abundance in RPKM (Reads Per Kilobase of transcript per Million mapped reads) was estimated using Cufflinks (Trapnell et al., 2012). Analysis of differential gene expression was performed using Cuffdiff (Trapnell et al., 2012). The fold-changes of differential expression was calculated by log2(RPKM ratio), with a pseudo-count of 1 added to each RPKM value. To identify the homologs to the significantly differentially expressed transcripts, the sequences were used as query for BLASTN search against the publicly available genomes in the Phytozome^2^ using an *E*-value cutoff of 1.0E-3.

### sORF Annotation

ORFfinder (Wheeler et al., 2003) was used to annotate putative ORFs within the differentially expressed sRNAs. We searched for 6 translational frames with the standard genetic code between 30 and 300 nucleotides for each prediction and selected the longest full-length sORF (including start and stop codon). The subcellular localization was predicted for each sORF using Loctree3 (Goldberg et al., 2014).

### miRNA Identification

The small RNA sequencing reads were filtered to remove adaptors and low-quality bases by FastQC (Andrews, 2010). The sequencing reads were processed to remove adaptors and cleaned by Q30 value. Reads with over 20% bases less than Q30, and N base more than 10% were filtered. The clean reads were further selected by length between 18 and 30 bp for miRNA identification process. The miRDP1.3 package (Yang and Li, 2011) was used for miRNA precursor prediction with size set at 250 nt. To validate the miRNAs, the secondary structure and reads distribution were evaluated to generate the final set of miRNAs (Yin et al., 2016). The precursors and mature sequences were aligned with the searching toolbox in PNRD database for annotation of miRNAs (Yi et al., 2015). To define the conservation of miRNAs, a 17 bp match in the mature sequences were used to identify conserved miRNA families, allowing two mismatches. To quantify the abundance of miRNA, transcripts per million (TPM) value was defined as “counts of read mapped to miRNA^∗^1,000,000”/”reads mapped to reference genome” (Fahlgren et al., 2007). To identify differentially expressed miRNA DESeq2 software was used, and expression folder change greater than 2 and *p*-value (Benjamini-Hochberg FDR corrected) less than 0.01 were selected (Love et al., 2014). The miRNA expression data were normalized by row z-score method. Hierarchical clustering of gene expression was performed by clustergram function in MATLAB Bioinformatics toolbox with minor changes.

### Target Prediction and Degradome Analysis

The miRNA targets were annotated by standard settings of psRNATarget (Dai and Zhao, 2011) with maximum expectation value 2.0. The transcripts of *P. deltoides* (WV94_445 v2.1) and *P. trichocarpa* (Nisqually-1 v3.0) were downloaded from Phytozome 12^3^ while the transcripts for *L. bicolor* (20110203) and *R. irregularis* (Gloin1_GeneCatalog_transcripts_20120510.nt) was downloaded from MycoCosm^4^. To validate the targets, the degradome datasets in *P. trichocarpa* were downloaded from NCBI Short Read Archive (SRR4010497, SRR4010498). The clean reads from degradome library were obtained for target site identification. The CleaveLand4.0 pipeline (Addo-Quaye et al., 2009) was used for target scanning. The reads were mapped to transcript datasets and the alignment scores and *p*-value were calculated according the signatures (abundances of potential slicing end based on reads distributions). The t-plots were generated to visualize the miRNA directed slicing to targets and *p*-value of less than 0.05 was used to identify highly confident targets. Gene ontology enrichment for biological processes and molecular functions were performed online at PopGenIE^5^ and visualized with REVIGO using default settings (Sjödin et al., 2009; Supek et al., 2011).

## Results

### Differential Expression of sRNAs During Populus-AmF/EmF Colonization

**Figure 1:**
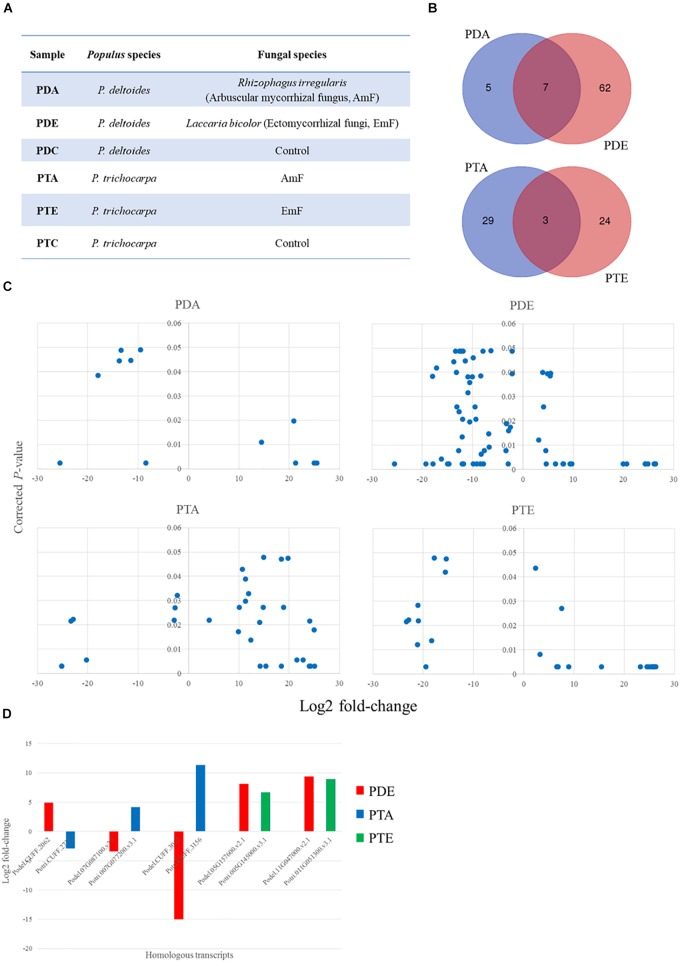
sRNA landscape of *Populus* species in response to the arbuscular mycorrhizal fungus (AmF), *Rhizophagus irregularis* and the ectomycorrhizal fungus (EmF), *Laccaria bicolor*. **(A)** *Populus* species and corresponding treatments used in the current study; **(B)** Venn diagram showing the transcript overlap from the different treatments in *P. deltoides* and *P. trichocarpa*; **(C)** Significant differentially expressed transcripts from the controls (*p* ≤ 0.05) and greater/less than two-fold change in the expression level; and **(D)** comparison of fold-change and trend for conserved transcripts across species. Transcript IDs and fold change can be found in Supplementary Tables S1, S2 and transcript sequences can be found in Supplementary Files S1, S2.

The AmF, *R. irregularis*, and the EmF, *L. bicolor*, were able to colonize both species of *Populus*, i.e., *P. deltoides* and *P. trichocarpa*, though colonization rates were higher for *P. trichocarpa* (Table 1). To identify differentially expressed sRNAs in response to the AmF and EmF treatments, we performed RNA-sequencing (RNA-Seq) of RNA fragments less than 300 nt isolated from roots of *P. deltoides* colonized with the AmF *R. irregularis* or the EmF *L. bicolor* (PDA and PDE, respectively) and from roots of *P. trichocarpa* colonized with *R. irregularis* or *L. bicolor* (PTA and PTE, respectively, Figure 1A). Roots without fungal inoculation from *P. deltoides* and *P. trichocarpa* were used as controls (PTC and PDC, respectively). Thus, a total of 18 sRNA-sequencing libraries (three biological replicates per treatment PDA, PDE, PTA, PTE, PDC, and PTC) were constructed and sequenced. Following trimming, the RNA-Seq reads were mapped to the respective *P. trichocarpa* v3.0 and *P. deltoides* WV94 v2.1 genome sequences (Table 2). Based on a corrected *p ≤* 0.05 and > 2-fold change in expression compared to the controls, we found 81 and 59 differentially expressed transcripts in *P. deltoides* and *P. trichocarpa*, respectively, relative to the control (Supplementary Tables S1, S2 and Supplementary Files S1, S2). Among the 81 fungal-responsive transcripts in *P. deltoides*, 12 and 69 transcripts were differentially expressed in the PDA and PDE interactions, respectively. Furthermore, among the 59 fungal-responsive transcripts in *P. trichocarpa*, 32 and 27 transcripts were differentially expressed in the PTA and PTE interactions, respectively. Interestingly, the differential transcripts were mostly specific to each plant-fungal combination, except for 7 transcripts that were shared between the PDA and PDE (all down-regulated except Podel.CUFF.90 which was up-regulated) and 3 transcripts shared between PTA and PTE (two down-regulated while Potri.013G036500 showed an opposite trend in each treatment; Figure 1B). The expression fold-change trend is shown in Figure 1C and Supplementary Tables S1, S2. Across species comparison showed that 5 homologous transcripts could be detected between species, of which three homologous transcript pairs showed the opposite expression trend between PDE and PTA and the other two homologous transcripts pairs were up-regulated in PDE and PTE (Figure 1D). These results suggest that, to a large extent, the expression of the sRNAs were distinct for each plant species within corresponding fungal treatments despite both fungi having a mutualistic relationship with these plants.

**Table 1:** Frequency of root colonization by arbuscular (AmF) and ectomycorrhizal (EmF) mycorrhizal fungus in *Populus deltoides* (PD) and *Populus trichocarpa* (PT).

Treatments	AmF colonization (%)		EmF colonization (%)	
	PD	PT	PD	PT
Control	0	0	0	0
Inoculated	72^a^ (^+/-^5)	87^a^ (^+/-^5)	34^b^ (^+/-^5)	57^b^ (^+/-^5)

*Root colonization rate was measured at 2 months post-inoculation (^+/-^*Standard Deviation)*. *^*aorb*^Different letters within each line denote significant differences at P ≤ 0.05.**

**Table 2:** Total number of RNA-Seq reads (in the range of three biological replicates per genotype/treatment), following trimming, that were mapped to *Populus deltoides* WV94 v2.1 and *Populus trichocarpa* genome v3.0 available on Phytozome 12.

Tissue	Treatment	Total reads	Reads mapped to respective genome	Percent mapped (%)
*P. deltoides*	Control-1	5749345	2611186	45.4
*P. deltoides*	Control-2	3014126	1940271	64.4
*P. deltoides*	Control-3	3075042	2066300	67.2
*P. deltoides*	*Rhizophagus irregularis*-1	4980677	2539132	51.0
*P. deltoides*	*Rhizophagus irregularis*-2	2993283	1524495	50.9
*P. deltoides*	*Rhizophagus irregularis*-3	2206148	1244202	56.4
*P. deltoides*	*Laccaria bicolor*-1	3564011	666296	18.7
*P. deltoides*	*Laccaria bicolor*-2	2080970	712191	34.2
*P. deltoides*	*Laccaria bicolor*-3	2232290	603916	27.1
*P. trichocarpa*	Control-1	2612622	1478334	56.6
*P. trichocarpa*	Control-2	1928877	982300	50.9
*P. trichocarpa*	Control-3	2744908	1280051	46.6
*P. trichocarpa*	*R. irregularis*-1	4234040	2492972	58.9
*P. trichocarpa*	*R. irregularis*-2	3012095	1482320	49.2
*P. trichocarpa*	*R. irregularis*-3	1968986	775833	39.4
*P. trichocarpa*	*L. bicolor*-1	4053717	781250	19.3
*P. trichocarpa*	*L. bicolor*-2	2063636	516891	25.0
*P. trichocarpa*	*L. bicolor*-3	1486150	268589	18.1

Apart from the 17 transcripts that were previously annotated within the respective genomes (Supplementary Table S3), the remaining significantly differentially expressed fungus-responsive plant sRNAs (with name starting as “CUFF”) are located in the genomic regions without previously annotated genes. From the 17 previously annotated genes, 6 were proteins of known function (e.g., MYB-LIKE DNA-BINDING PROTEIN and AMMONIUM TRANSPORTER 1 MEMBER), while the remaining 11 genes were annotated as proteins of unknown function (Supplementary Table S3). We assessed the conservation of the differentially expressed sRNA across 63 plant genomes available on Phytozome 12.0 and found homology to 47 *P. deltoides* and 20 *P. trichocarpa* sRNAs in at least one genome analyzed (*E*-value ≤ 03; Supplementary Table S4).

### Novel sORFs in Populus in Response to Mycorrhizal Fungi

Since sRNAs can potentially encode sORFs (Li et al., 2014; Ruiz-Orera et al., 2014), we used ORFfinder (Wheeler et al., 2003) to scan the differentially expressed sRNAs for potential sORF translations. In total, we could predict 22 and 6 full-length sORFs from the treatments in *P. deltoides* and *P. trichocarpa*, respectively (Supplementary Tables S5, S6). The shortest predicted sORF was 39 bp (12 AA) while the longest was 273 bp (90 AA). The transcript, coding, and protein sequences of these sORFs can be found in Supplementary Files S1, S2, S5, S6. Prediction of subcellular localization showed that 26 sORFs are secreted extracellularly while a single sORF is targeted to the nucleus and another to the chloroplast (Supplementary Table S7).

### Identification of miRNAs in Populus in Response to Mycorrhizal Fungi

**Figure 2:**
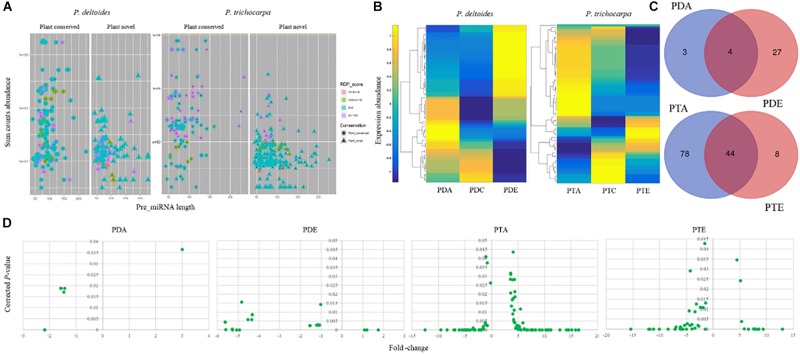
miRNA landscape *Populus deltoides* and *P. trichocarpa* roots colonized with *Rhizophagus irregularis* (PDA and PTA, respectively) and *Laccaria bicolor* (PDE and PTE, respectively). **(A)** miRNAs identified were classified as “Plant conserved” or “Plant novel”; **(B)** Hierarchical clustering of differential expression in transcripts per million of identified miRNAs; **(C)** Venn showing significant differentially expressed miRNAs that were common and unique miRNAs to each treatment; and **(D)** Fold-change and the significance (*p* ≤ 0.05) of differentially expressed miRNAs.

To gain insight into the role of miRNAs in mutualistic interactions, we analyzed the sRNA datasets to identify miRNAs that were responsive to the different mycorrhizal treatments. Using the miRDeep-P pipeline (Yang and Li, 2011), we identified 287 putative miRNAs in *P. deltoides* and 357 putative miRNAs in *P. trichocarpa*, including precursor and mature sequences (Supplementary Files S3, S4 and Supplementary Tables S8, S9). In *P. deltoides and P. trichocarpa*, 174 and 130 miRNAs were classified into “Plant conserved” miRNA families, respectively, and 113 and 227 miRNAs were classified as “Plant novel” miRNAs, respectively (Figure 2A and Supplementary Tables S8, S9). Note that the corresponding known families for the miRNAs identified in this study can found in Supplementary Tables S8, S9 while the miRNA abundance expressed as TPM for each treatment can be found in Supplementary Tables S10, S11. To refine the list of miRNAs, we focused on those that were significantly differentially expressed (negative binomial test, FDR corrected *p* ≤ 0.05; fold-change > 2; Soneson and Delorenzi, 2013) between the treatment and control. In this regard, we only considered miRNAs with greater than 50 counts in total and found 34 and 130 unique miRNAs with significant differential expression in *P. deltoides* and *P. trichocarpa*, respectively, including Plant Novel miRNAs (Supplementary Tables S12, S13). Hierarchical clustering of differentially expressed miRNAs showed distinct differences in expression pattern in response to EmF and AmF in both *P. deltoides* and *P. trichocarpa* (Figure 2B).

We next investigated whether each species shared a common set of miRNAs during the different mutualistic symbiosis. We found that in *P. deltoides* 4 miRNAs were common between PDA and PDE and in *P. trichocarpa* 44 miRNAs were common between PTA and PTE (Figure 2C and Supplementary Tables S12, S13). In *P. deltoides*, all 4 common miRNAs were down-regulated relative to the control while in *P. trichocarpa*, 16 shared miRNAs were up-regulated, 23 were down-regulated and 5 showed the opposite fold-change trend (Table 3). Further, this analysis also revealed that, within species, 3 and 27 miRNAs were unique to PDA and PDE, respectively, while 78 and 8 miRNAs were unique to PTA and PTE, respectively (Figure 2C and Supplementary Tables S12, S13). We further compared the Best-Hit of the significantly differentially expressed miRNAs across species, i.e., PDA vs. PTA and PDE vs. PTE (Supplementary Tables S12, S13). We did not find any miRNAs in common between different species colonized by a common fungus (data not shown). Also, we compared the differential transcripts listed in DATA 1 and 2 with the miRNA precursor sequences using the CAP3 program implemented in http://biosrv.cab.unina.it/webcap3/ and identified that five differential transcripts were miRNA precursors (Supplementary Table S14). The miRNA expression fold-change trend can be seen in Figure 2D and Supplementary Tables S12, S13. In *P. trichocarpa*, we found that 23 and 7 miRNAs were only detected in the PTA and PTE treatments, respectively, and not in the controls.

**Table 3:** Expression fold-change trend of miRNAs in *Populus deltoides* and *P. trichocarpa* in response to *Rhizophagus irregularis* (PDA and PTA, respectively) and *Laccaria bicolor* (PTA and PTE, respectively).

		Down-regulated miRNAs in *P. deltoides*	
miRNA ID	miRNA name	**PDA**	**PDE**
		
Pde_miRNA_219	pde-MIRf11885b	-1.57	-5.28
Pde_miRNA_228	pde-MIR393f	-1.58	-5.6
Pde_miRNA_155	pde-MIR396a	-1.47	-5.08
Pde_miRNA_156	pde-MIRf12503b	-1.43	-4.93

		**Up-regulated miRNAs in *P. trichocarpa***	

		**PTA**	**PTE**
		
Ptr_miRNA_178	ptr-miR482a	7.6	5.31
Ptr_miRNA_277	ptr-miR164c	5.29	12.95
Ptr_miRNA_299	ptr-miR166g	14.54	9.39
Ptr_miRNA_248	ptr-miR162a	5.06	7.8
Ptr_miRNA_206	ptr-miR167g	6.2	7.77
Ptr_miRNA_62	ptr-miR2912x	3.58	4.46
Ptr_miRNA_179	ptr-miR167a	5.19	8.56
Ptr_miRNA_298	ptr-miR159b	10.31	5.83
Ptr_miRNA_15	ptr-miR812a	4.43	12.96
Ptr_miRNA_342	ptr-miR156h	Not detected in control, induced in PTA	Not detected in control, induced in PTE
Ptr_miRNA_339	ptr-miR171i	Not detected in control, induced in PTA	Not detected in control, induced in PTE
Ptr_miRNA_330	ptr-miR482a	Not detected in control, induced in PTA	Not detected in control, induced in PTE
Ptr_miRNA_282	ptr-miR319a	Not detected in control, induced in PTA	Not detected in control, induced in PTE
Ptr_miRNA_247	ptr-miR159a	Not detected in control, induced in PTA	Not detected in control, induced in PTE
Ptr_miRNA_113	ptr-miR166d	Not detected in control, induced in PTA	Not detected in control, induced in PTE
Ptr_miRNA_131	ptr-miR156g	Not detected in control, induced in PTA	Not detected in control, induced in PTE

		**Down-regulated miRNAs in *P. trichocarpa***	

		**PTA**	**PTE**
		
Ptr_miRNA_155	ptr-miR2912g	-4.65	-4.84
Ptr_miRNA_473	ptr-miR2912w	-1.58	-2.67
Ptr_miRNA_44	ptr-miR2912v	-6.92	-10.09
Ptr_miRNA_160	Ptr_miRNA_160	-12.58	-12.07
Ptr_miRNA_210	ptr-miR2912m	-3.37	-4.3
Ptr_miRNA_311	ptr-miR2111b	-1.19	-6
Ptr_miRNA_357	ptr-miR156j	-4.59	-7.08
Ptr_miRNA_16	ptr-miR2912i	-8.4	-15.39
Ptr_miRNA_255	ptr-miR2912n	-1.08	-4.25
Ptr_miRNA_209	Ptr_miRNA_209	-3.99	-6.1
Ptr_miRNA_105	ptr-miR482d	-6.09	-1.43
Ptr_miRNA_328	ptr-miR839	-9.57	-11.79
Ptr_miRNA_329	ptr-miR156d	-5.59	-11.13
Ptr_miRNA_135	ptr-miRf11683	-1.58	-3.39
Ptr_miRNA_29	ptr-miR6427	-1.54	-3.14
Ptr_miRNA_110	ptr-miR2912b	-2.31	-6.18
Ptr_miRNA_156	ptr-miR2912h	-4.33	-6.07
Ptr_miRNA_369	ptr-miR476	-0.82	-1.56
Ptr_miRNA_370	ptr-miR2912s	-3.26	-3.4
Ptr_miRNA_187	ptr-miR172b	-2.15	-3.95
Ptr_miRNA_106	Ptr_miRNA_106	-6.07	-7.17
Ptr_miRNA_21	Ptr_miRNA_21	-2.01	-5.36
Ptr_miRNA_232	Ptr_miRNA_232	-5.29	-8.41

		**miRNAs showing opposite expression trend in *P. trichocarpa* treatments**	

		**PTA**	**PTE**
		
Ptr_miRNA_191	ptr-miR475b	-2.05	7.58
Ptr_miRNA_134	ptr-miR2912e	-0.81	5.14
Ptr_miRNA_275	ptr-miR166f	11.17	-2.37
Ptr_miRNA_103	ptr-miR166b	10.58	-1.38
Ptr_miRNA_337	ptr-miR396c	5.18	-4.97

### Identification of Target Genes of miRNAs in Populus in Response to Mycorrhizal Fungi

**Figure 3:**
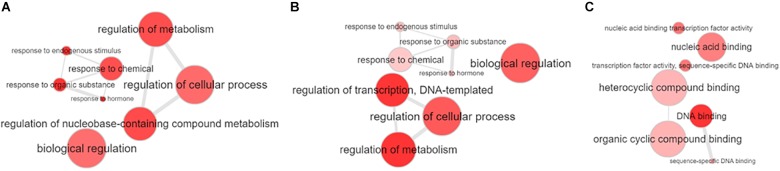
Gene ontology enrichment of the target genes of the miRNAs identified in *Populus deltoides* and *P. trichocarpa* roots colonized with the arbuscular mycorrhizal fungus (AmF), *Rhizophagus irregularis*, and the ectomycorrhizal fungus (EmF), *Laccaria bicolor*. **(A)** Gene ontology biological processes for *P. deltoides* with AmF and EmF; **(B)** Gene ontology biological processes for *P. trichocarpa* with AmF and EmF; and **(C)** Gene ontology molecular processes for *P. trichocarpa* with AmF and EmF. Enrichment was performed online at PopGenIE (http://popgenie.org/) and visualized with REVIGO (Sjödin et al., 2009; Supek et al., 2011).

To determine the biological function of all identified miRNAs, we searched the “degradome” libraries of public databases for potential gene targets in *Populus*. While we identified 61 gene targets to 41 unique miRNAs in *P. deltoides* and 117 gene targets to 46 unique miRNAs in *P. trichocarpa* (*p ≤* 0.05; Supplementary Tables S15, S16), there were only 12 and 59 unique gene targets (including alleles) to 7 and 25 unique differentially expressed *P. deltoides* and *P. trichocarpa* miRNAs, respectively (Supplementary Table S17). We also identified putative targets to the “Plant novel” miRNAs category, i.e., 10 miRNA-gene targets in *P. deltoides* and 18 miRNA-gene targets in *P. trichocarpa* (Table 4). In both *P. deltoides* and *P. trichocarpa*, gene ontology enrichment analysis of the miRNA targets (Supplementary Tables S15, S16) for biological processes included terms associated with regulation of metabolism, response to hormones and endogenous stimulus and regulation of transcription (Figure 3A,B) while molecular functions in *P. trichocarpa* were enriched in transcription factor activity, nucleic acid binding and DNA binding (Figure 3C). There were no enrichments for molecular functions in *P. deltoides*.

**Table 4:** Gene targets predicted for the “plant novel” *Populus* miRNAs in response to *Rhizophagus irregularis* and *Laccaria bicolor*.

miRNA ID	miRNA name	Target gene	Annotation of target gene
Pde_miRNA_73	pde-MIR2628b	Podel.02G280600.1	Photosystem I subunit IV (psaE)
Pde_miRNA_85	pde-MIR857	Podel.03G089000.1	Beta-amylase/Saccharogen amylase
Pde_miRNA_115	pde-MIRf12523d	Podel.03G183100.1	CALMODULIN-BINDING TRANSCRIPTION ACTIVATOR
Pde_miRNA_21	pde-MIR395a	Podel.09G062800.1	ZINC FINGER CCCH DOMAIN-CONTAINING PROTEIN 56
Pde_miRNA_187	pde-MIRf10448b	Podel.07G012300.1	MATE EFFLUX FAMILY PROTEIN
Pde_miRNA_133	pde-MIR2912h	Podel.04G165900.1	FRUCTOSE-BISPHOSPHATE ALDOLASE 2, CHLOROPLASTIC-RELATED
Pde_miRNA_149	pde-MIRf12506i	Podel.01G087300.1	Mediator of RNA polymerase II transcription subunit 10
Pde_miRNA_225	pde-MIRf10282	Podel.19G057400.1	D-XYLOSE-PROTON SYMPORTER-LIKE 1-RELATED
Pde_miRNA_135	pde-MIR5715a	Podel.09G129400.1	FRUCTOSE-BISPHOSPHATE ALDOLASE 2, CHLOROPLASTIC-RELATED
Pde_miRNA_53	pde-MIRf10816c	Podel.11G131500.1	POLYUBIQUITIN 3
Ptr_miRNA_169	ptr-miR166a	Potri.009G014500.2	Homeobox-leucine zipper family protein/lipid-binding START domain-containing protein
Ptr_miRNA_221	ptr-miRf10117a	Potri.005G091700.1	Serine carboxypeptidase-like 34
Ptr_miRNA_283	ptr-miR847	Potri.014G071000.1	Protein of unknown function
Ptr_miRNA_269	ptr-miRf10610b	Potri.002G154600.2	Ribosomal L5P family protein
Ptr_miRNA_105	ptr-miR482d	Potri.018G138500.1	NB-ARC domain-containing disease resistance protein
Ptr_miRNA_335	ptr-miR7748a	Potri.008G181000.5	Response regulator 11
Ptr_miRNA_409	ptr-miRf10535	Potri.001G038100.1	Serine-domain containing serine and sphingolipid biosynthesis protein
Ptr_miRNA_223	ptr-miRf10117d	Potri.001G234400.1	Nudix hydrolase homolog 9
Ptr_miRNA_85	ptr-miRf10350	Potri.007G013100.1	Ribosomal protein L2 family
Ptr_miRNA_215	ptr-miRf10010b	Potri.002G124200.5	Poly(A) binding protein 2
Ptr_miRNA_467	Ptr_miRNA_467	Potri.006G138600.1	Chaperonin 20
Ptr_miRNA_259	ptr-miRf10010c	Potri.002G124200.3	Poly(A) binding protein 2
Ptr_miRNA_71	ptr-miRf10010d	Potri.002G124200.5	Poly(A) binding protein 2
Ptr_miRNA_161	ptr-miRf10116a	Potri.005G091700.3	Serine carboxypeptidase-like 34
Ptr_miRNA_421	Ptr_miRNA_421	Potri.005G192300.1	Tubby-like protein 5
Ptr_miRNA_417	Ptr_miRNA_417	Potri.001G189700.1	NADP-malic enzyme 4
Ptr_miRNA_51	Ptr_miRNA_51	Potri.001G001500.1	RING/U-box superfamily protein
Ptr_miRNA_285	ptr-miR169h	Potri.010G218700.2	GTP binding Elongation factor 1 alpha family protein

### Identification of Putative Target Genes for Populus-Derived miRNAs in *R. irregularis* and *L. bicolor*

We predicted potential miRNA-gene targets in the respective fungi since small RNA may transverse between symbionts via extracellular vesicles to mediate RNA interference and effect physiological changes across species (Lefebvre and Lécuyer, 2017). We found 5 gene targets *in L. bicolor* for 14 significantly differentially expressed *P. deltoides* miRNAs, 4 gene targets in *L. bicolor* for 6 significantly differentially expressed *P. trichocarpa* miRNAs and 5 gene targets in *R. irregularis* for 5 significantly differentially expressed *P. trichocarpa* miRNAs (Supplementary Table S18). We did not find any genes targets in *R. irregularis* to the significantly differentially expressed *P. deltoides* miRNAs. The majority of the predicted gene targets within the respective fungi were hypothetical protein/predicted proteins while a transport protein particle complex subunit (TRAPP 20 K subunit) was a common target for several *P. deltoides* and *P. trichocarpa* miRNAs. In addition, despite an annotation as a hypothetical protein, the target genes of two miRNAs had a KOG description as transcription factors.

## Discussion

In the current study, we applied high-throughput RNA sequencing to describe the sRNA landscape of the root of two *Populus* species (*P. deltoides* and *P. trichocarpa)* colonized by an EmF, *L. bicolor*, or an AmF, *R. irregularis*, with specific focus on the sORF and miRNA response. A striking observation was that these beneficial plant-fungal interactions triggered the expression of otherwise intergenic regions of the genome. Furthermore, despite being mutualistic symbiosis, only a small number of these transcripts were conserved within the species between the two fungi (7 in *P. deltoides* and 3 in *P. trichocarpa;* Figure 1B). This may be a reflection of the different classes of fungi, i.e., arbuscular mycorrhizal vs. ectomycorrhizal, suggesting that at least on the plant side and for the sRNAs, mutualistic associations do not necessarily have a common response/regulatory mechanism. Additionally, only 5 transcripts were shared between *P. deltoides* and *P. trichocarpa* (Figure 1D), leading to a hypothesis that the molecular differences (i.e., the low number of sRNAs shared between two *Populus* species under two different fungal treatments) in the expression of plant small RNAs in response to fungi might contribute to the phenotypic difference in colonization efficiency (see Table 1; Tagu et al., 2001). This hypothesis needs to be tested using experimental approaches such as gain-of-function via gene overexpression and loss-of-function via gene knockout in the future.

While we do not know for certain what the end products of each sRNA is, several lines of evidence exist for the functionality of sORFs translated from sRNAs (Andrews and Rothnagel, 2014; Ruiz-Orera et al., 2014). Given that non-coding RNA may be an important source of new peptides, our predicted coding potential of the sRNAs advocates for a functional role for several sORFs in the symbiotic interactions. This is further supported by the predicted subcellular targeting of these sORFs (Supplementary Table S7). Recently, Plett et al. (2017) showed that several *Populus*-encoded SSPs (≤ 250 AA), including a novel SSP that was not annotated in the *P. trichocarpa* genome, could enter the fungal hyphae, localize to the nucleus and affect growth of *L. bicolor*.

The differential expression of a large number of miRNAs identified in response to the fungal treatments (34 in *P. deltoides* and 130 *P. trichocarpa*) suggest that the plant miRNAs are an important regulator of this mutualistic symbiosis. In this respect, our differentially expressed miRNA list (Supplementary Tables S12, S13) contained previously identified miRNAs that are previously reported to be involved in mutualistic interactions, albeit in different plant species such as *Medicago*, tomato and rice, including miR156, miR160, miR170, miR167, miR393, and miR396 (Bazin et al., 2013; Etemadi et al., 2014; Wu et al., 2016). Unlike the sRNA landscape, several significantly differentially expressed miRNAs were shared between PTA and PTE treatments (Figure 2C), indicating that the miRNA landscape and hence, the molecular mechanism imparted by this level of regulation may be conserved to a certain extent within species. The latter is further substantiated by the same expression trend seen for 39 of the 44 miRNAs in common between PTA and PTE and all 4 miRNAs in common between PDA and PDE, where both miRNAs were either up- or down-regulated in the respective treatments (highlighted in blue in Supplementary Tables S12, S3). We however, also noted that the expression pattern of miRNAs belonging to the same family did also vary within a treatment. For example, miR156 family targeting members of the squamosa promoter binding and binding-like transcription factors were up- and down-regulated in PTA (Ptr_miRNA_245 [ptr-miR156b], Ptr_miRNA_329 [ptr-miR156d], Ptr_miRNA_127 [ptr-miR156e], Ptr_miRNA_131 [ptr-miR156g], and Ptr_miRNA_433 [ptr-miR156k]; Supplementary Table S13). This suggests a very intricate role for members of the same miRNA family in these plant-microbe interactions, and further, indicates the specificity to which the miRNAs functions in these tested relationships.

Given that the number of miRNAs identified in *P. trichocarpa* was approximately 4 times higher than in *P. deltoides*, and that a large number of miRNAs were in common between PTA and PTE, many with the same expression trend, we hypothesize that species-specific responsive miRNAs may play an important role in the preferential colonization of *P. trichocarpa* over *P. deltoides*, as previously reported (Tagu et al., 2001; Labbé et al., 2011). For example, consistent with previous findings in *Solanum*, *Medicago*, and *Oryza*, miR393, which negatively regulates arbuscule formation via an obstruction to the auxin perception, was also down-regulated in PTA in our study (Ptr_miRNA_289 [ptr-miR393b]) but was absent in PDA. The predicted target for Ptr_miRNA_289 [ptr-miR393b] was Potri.002G207800.1, annotated as Transport Inhibitor Response I (TIR/F-box/RNI-like superfamily protein; Supplementary Table S17). When auxin is perceived, TIR of the E3 ligase receptor complex targets the Aux/IAA protein for degradation via ubiquitination which releases the Auxin Response Factors (ARFs) to allow auxin induced gene expression of their targets (Sukumar et al., 2013; Etemadi et al., 2014). Gene components of this auxin signaling are associated with root development where auxin typically stimulates lateral root development and inhibits root elongation (Casimiro et al., 2003; Sukumar et al., 2013). Interestingly, in our study, the two ARFs targets identified, Potri.002G055000.2 and Potri.011G091900.4, should have increased expression since their corresponding miRNA, Ptr_miRNA_407 (ptr-miR167b) was down-regulated as well; thereby supporting the auxin- perception and signaling previously described (Sukumar et al., 2013).

It is also possible that AmF colonization of the *P. deltoides* roots, which still occurs albeit at a low frequency, proceeds via alternative means. Lauressergues et al. (2012) showed that there was an up-regulation of miR171h during the colonization of *Medicago truncatula* roots by *R. irregularis.* The gene target of miR171h is a GRAS transcription factor, NSP2, that is believed to be involved in the biosynthesis of a hormone, strigolactone which promotes germination and growth of AmF. Interestingly, the overexpression of miR171h is associated with a decrease in the target gene expression and an overall decrease in the fungal colonization; while the overexpression of a miR171h-resistant NSP2 gene showed over-colonization. These results suggest that that miR171h is a negative regulator of NSP2 thereby preventing over-colonization. Another study reported increased NSP2 gene levels in mycorrhizal roots despite the presence of miR171h; therefore, suggesting the presence of an addition component regulating miR171h-NSP2 (Hofferek et al., 2014). In our study, we noted an up-regulation of miR171 in PTA and PTE (Ptr_miRNA_339 [ptr-miR171i], common between the treatments; Supplementary Table S13) and down-regulation of miR171 in PDA and PDE (Pde_miRNA_228 [pde-MIR393f], also common between the treatments; Supplementary Table S12). According to Lauressergues et al. (2012), if miR171 is down-regulated, as in the case of PDA, then NSP expression will not be down-regulated thereby increasing mycorrhizal colonization. The alternative proposed by Hofferek et al. (2014) is also true where the NSP2 transcript was elevated during the progression of mycorrhizal colonization independent of miR171h levels, and in the case of PDA will once again promote increased colonization. This hypothesized route may assist in the symbiotic interaction in *P. deltoides* which has a lower propensity for colonization. A similar trend was noted with pathogenic fungi where a specific miRNAs were either repressed or induced in different plant species, suggesting functional differences in these alternant plants to common pathogens (Gupta et al., 2014). Moreover, as AmF symbiosis progresses miR171 levels should increase (Lauressergues et al., 2012; Hofferek et al., 2014). Ptr_miRNA_339 (ptr-miR171i) was upregulated in the PTA interactions. If miR171 is a negative regulator of NSP2 thereby preventing over-colonization (Lauressergues et al., 2012), the increase in expression in miR171 in PDA could serve as a checkpoint to prevent an imbalance in the mutualism between *P. trichocarpa*, with its high propensity to be colonized, and the fungus. Future experimental characterization and profiling of these responsive miRNAs and targets should shed some light on this method of colonization and control.

We were also able to identify putative gene targets to the significantly differentially expressed conserved and novel *Populus* miRNAs (Supplementary Table S17). Our gene ontology enrichment analysis showed consistency with a previous genome-wide study investigating the miRNAs responsive to arbuscular mycorrhiza in tomato, including transcription regulation, biological regulation and response to stimulus (Figure 3; Wu et al., 2016). It was interesting to note that the molecular functions of *P. trichocarpa* in mycorrhizal associations included transcription factor activity, nucleic acid binding and DNA binding. Plett et al. (2017) showed that SSPs could enter the hyphal nucleus of *L. bicolor* to affect the fungal physiology. Closer inspection of the miRNAs and their corresponding gene target revealed a large number of transcription factors associated with root formation including growth-regulating factors (GRFs), homeodomain-leucine zipper transcription factor, AP2 transcription factor and MYB transcription factors (Supplementary Table S17). We also found phytohormone related genes including ethylene-responsive transcription factor and auxin response factor, as noted in other studies.

In plants, sRNAs move to adjacent cells via the plasmodesmata and systemically via the vasculature system (Baulcombe, 2004). Recently, it was shown that cross-kingdom/organism RNA interference occurs via exosome-like extracellular vesicles that transfer the host sRNAs to the interacting partner at the infection site, which then consequently causes gene silencing (Wang et al., 2017; Cai et al., 2018; Shahid et al., 2018). Therefore, it is highly probable that plant-encoded sRNAs, during mycorrhizal symbiosis, extends beyond the self-regulation. In support of this hypothesis, we used the plant-encoded miRNAs to predict fungal gene targets in the respective genomes. We identified several fungal gene targets to the *Populus*-derived miRNAs, including transport proteins, transcription factors and several genes encoding proteins of unknown function in the two fungi analyzed (Supplementary Table S18).

Finally, our study adds to the growing number of reports suggesting the active role of plants in mutualistic associations with microbes (Akiyama et al., 2005; Scharf et al., 2016; Plett et al., 2017). While a number of studies have focused on the arbuscular mycorrhizal-responsive miRNAs (Devers et al., 2011; Wu et al., 2016), genome-wide studies on the endomycorrhizal-responsive miRNAs is in its infancy. Given the role of miRNAs in fungal interactions (Bazin et al., 2013; Etemadi et al., 2014) and the expanding evidence for sORFs in plant physiology and mutualistic symbiosis (Hanada et al., 2007, 2013; Andrews and Rothnagel, 2014; Plett et al., 2017), the current study provides a useful resource of potential regulators that are involved in arbuscular/endomycorrhizal symbiosis with *Populus*.

## Data Availability

The Department of Energy (DOE) will provide public access to these results of federally sponsored research in accordance with the DOE Public Access Plan (http://energy.gov/downloads/doe-public-access-plan). The RNA-Seq reads, along with the processed data (including the sequence and abundance measurements of differentially-expressed transcripts) are deposited in NCBI GEO (https://www.ncbi.nlm.nih.gov/geo/) with the accession code GSE117158.

## Author Contributions

XY and JL conceived and supervised the project. RM and HY carried out bioinformatic analysis, interpreted the data, and wrote the manuscript. SJ, RH, and PV carried out the experiments. GT and FL supervised the study and interpreted the data. All authors read and commented on the manuscript.

## Conflict of Interest Statement

The authors declare that the research was conducted in the absence of any commercial or financial relationships that could be construed as a potential conflict of interest.

## Footnotes

1 https://phytozome.jgi.doe.gov

2 www.phytozome.com

3 https://phytozome.jgi.doe.gov/pz/portal.html

4 https://genome.jgi.doe.gov/Lacbi2/Lacbi2.home.html

5 http://popgenie.org/

## References

[B1] Abbott L. K. Robson A. (1977). Growth stimulation of subterranean clover with vesicular arbuscular mycorrhizas. *Aust. J. Agric. Res.* 28 639–649. 10.1071/AR9770639

[B2] Addo-Quaye C. Miller W. Axtell M. J. (2009). CleaveLand: a pipeline for using degradome data to find cleaved small RNA targets. *Bioinformatics* 25 130–131. 10.1093/bioinformatics/btn604 19017659 PMC3202307

[B3] Akiyama K. Matsuzaki K. Hayashi H. (2005). Plant sesquiterpenes induce hyphal branching in arbuscular mycorrhizal fungi. *Nature* 435 824–827. 10.1038/nature03608 15944706

[B4] Al-Karaki G. McMichael B. Zak J. (2004). Field response of wheat to arbuscular mycorrhizal fungi and drought stress. *Mycorrhiza* 14 263–269. 10.1007/s00572-003-0265-2 12942358

[B5] Amaral P. P. Dinger M. E. Mattick J. S. (2013). Non-coding RNAs in homeostasis, disease and stress responses: an evolutionary perspective. *Brief. Funct. Genomics* 12 254–278. 10.1093/bfgp/elt016 23709461

[B6] Andrews S. (2010). *FastQC: A Quality Control Tool for High Throughput Sequence Data.* Available at: http://www.bioinformatics.babraham.ac.uk/projects/fastqc

[B7] Andrews S. J. Rothnagel J. A. (2014). Emerging evidence for functional peptides encoded by short open reading frames. *Nat. Rev. Genet.* 15 193–204. 10.1038/nrg3520 24514441

[B8] Aspden J. L. Eyre-Walker Y. C. Phillips R. J. Amin U. Mumtaz M. A. S. Brocard M. (2014). Extensive translation of small open reading frames revealed by Poly-Ribo-Seq. *eLife* 3:e03528. 10.7554/eLife.03528 25144939 PMC4359375

[B9] Baulcombe D. (2004). RNA silencing in plants. *Nature* 431 356–363. 10.1038/nature02874 15372043

[B10] Bazin J. Khan G. A. Combier J. P. Bustos-Sanmamed P. Debernardi J. M. Rodriguez R. (2013). miR396 affects mycorrhization and root meristem activity in the legume *Medicago truncatula*. *Plant J.* 74 920–934. 10.1111/tpj.12178 23566016

[B11] Bolger A. M. Lohse M. Usadel B. (2014). Trimmomatic: a flexible trimmer for Illumina sequence data. *Bioinformatics* 30 2114–2120. 10.1093/bioinformatics/btu170 24695404 PMC4103590

[B12] Bonfante P. Genre A. (2010). Mechanisms underlying beneficial plant–fungus interactions in mycorrhizal symbiosis. *Nat. Commun.* 1:48. 10.1038/ncomms1046 20975705

[B13] Branscheid A. Sieh D. Pant B. D. May P. Devers E. A. Elkrog A. (2010). Expression pattern suggests a role of MiR399 in the regulation of the cellular response to local Pi increase during arbuscular mycorrhizal symbiosis. *Mol. Plant Microbe Interact.* 23 915–926. 10.1094/MPMI-23-7-0915 20521954

[B14] Brosnan C. A. Voinnet O. (2011). Cell-to-cell and long-distance siRNA movement in plants: mechanisms and biological implications. *Curr. Opin. Plant Biol.* 14 580–587. 10.1016/j.pbi.2011.07.011 21862389

[B15] Brundrett M. Bougher N. Dell B. Grove T. (1996). *Working Ylith Mycorrhizas in Forestry and Agriculture.* Canberra: Australian Centre for International Agricultural Research.

[B16] Bryan A. C. Jawdy S. Gunter L. Gjersing E. Sykes R. Hinchee M. A. (2016). Knockdown of a laccase in *Populus deltoides* confers altered cell wall chemistry and increased sugar release. *Plant Biotechnol. J.* 14 2010–2020. 10.1111/pbi.12560 26997157 PMC5043505

[B17] Cai Q. Qiao L. Wang M. He B. Lin F.-M. Palmquist J. (2018). Plants send small RNAs in extracellular vesicles to fungal pathogen to silence virulence genes. *Science* 360 1126–1129. 10.1126/science.aar4142 29773668 PMC6442475

[B18] Casimiro I. Beeckman T. Graham N. Bhalerao R. Zhang H. Casero P. (2003). Dissecting Arabidopsis lateral root development. *Trends Plant Sci.* 8 165–171. 10.1016/S1360-1385(03)00051-7 12711228

[B19] Castellana N. E. Payne S. H. Shen Z. Stanke M. Bafna V. Briggs S. P. (2008). Discovery and revision of *Arabidopsis* genes by proteogenomics. *Proc. Natl. Acad. Sci. U.S.A.* 105 21034–21038. 10.1073/pnas.0811066106 19098097 PMC2605632

[B20] Chekanova J. A. (2015). Long non-coding RNAs and their functions in plants. *Curr. Opin. Plant Biol.* 27 207–216. 10.1016/j.pbi.2015.08.003 26342908

[B21] Chen X. (2008). MicroRNA metabolism in plants. *Curr. Top. Microbiol. Immunol.* 320 117–136. 10.1007/978-3-540-75157-1_6 18268842 PMC2570777

[B22] Chen X. (2012). Small RNAs in development–insights from plants. *Curr. Opin. Genet. Dev.* 22 361–367. 10.1016/j.gde.2012.04.004 22578318 PMC3419802

[B23] Daguerre Y. Plett J. M. Veneault-Fourrey C. (2017). “Signaling pathways driving the development of ectomycorrhizal symbiosis,” in *Molecular Mycorrhizal Symbiosis*, ed. Martin F. (Hoboken, NJ: John Wiley & Sons, Inc), 141–157.

[B24] Dai X. Zhao P. X. (2011). psRNATarget: a plant small RNA target analysis server. *Nucleic Acids Res.* 39 W155–W159. 10.1093/nar/gkr319 21622958 PMC3125753

[B25] Devers E. A. Branscheid A. May P. Krajinski F. (2011). Stars and symbiosis: microRNA-and microRNA^∗^-mediated transcript cleavage involved in arbuscular mycorrhizal symbiosis. *Plant Physiol.* 156 1990–2010. 10.1104/pp.111.172627 21571671 PMC3149951

[B26] Duponnois R. Garbaye J. (1991). Mycorrhization helper bacteria associated with the Douglas fir-*Laccaria laccata* symbiosis: effects in aseptic and in glasshouse conditions. *Ann. Sci. For.* 48 239–251. 10.1051/forest:19910301

[B27] el Zahar Haichar F. Santaella C. Heulin T. Achouak W. (2014). Root exudates mediated interactions belowground. *Soil Biol. Biochem.* 77 69–80. 10.1016/j.soilbio.2014.06.017

[B28] Etemadi M. Gutjahr C. Couzigou J.-M. Zouine M. Lauressergues D. Timmers A. (2014). Auxin perception is required for arbuscule development in arbuscular mycorrhizal symbiosis. *Plant Physiol.* 166 281–292. 10.1104/pp.114.246595 25096975 PMC4149713

[B29] Fahlgren N. Howell M. D. Kasschau K. D. Chapman E. J. Sullivan C. M. Cumbie J. S. (2007). High-throughput sequencing of *Arabidopsis* microRNAs: evidence for frequent birth and death of MIRNA genes. *PLoS One* 2:e219. 10.1371/journal.pone.0000219 17299599 PMC1790633

[B30] Galindo M. I. Pueyo J. I. Fouix S. Bishop S. A. Couso J. P. (2007). Peptides encoded by short ORFs control development and define a new eukaryotic gene family. *PLoS Biol.* 5:e106. 10.1371/journal.pbio.0050106 17439302 PMC1852585

[B31] Goldberg T. Hecht M. Hamp T. Karl T. Yachdav G. Ahmed N. (2014). LocTree3 prediction of localization. *Nucleic Acids Res.* 42 W350–W355. 10.1093/nar/gku396 24848019 PMC4086075

[B32] Großhans H. Filipowicz W. (2008). Molecular biology: the expanding world of small RNAs. *Nature* 451 414–416. 10.1038/451414a 18216846

[B33] Gupta O. P. Sharma P. Gupta R. K. Sharma I. (2014). Current status on role of miRNAs during plant–fungus interaction. *Physiol. Mol. Plant Pathol.* 85 1–7. 10.1016/j.pmpp.2013.10.002

[B34] Hanada K. Higuchi-Takeuchi M. Okamoto M. Yoshizumi T. Shimizu M. Nakaminami K. (2013). Small open reading frames associated with morphogenesis are hidden in plant genomes. *Proc. Natl. Acad. Sci. U.S.A.* 110 2395–2400. 10.1073/pnas.1213958110 23341627 PMC3568369

[B35] Hanada K. Zhang X. Borevitz J. O. Li W.-H. Shiu S.-H. (2007). A large number of novel coding small open reading frames in the intergenic regions of the *Arabidopsis thaliana* genome are transcribed and/or under purifying selection. *Genome Res.* 17 632–640. 10.1101/gr.5836207 17395691 PMC1855179

[B36] Hofferek V. Mendrinna A. Gaude N. Krajinski F. Devers E. A. (2014). MiR171h restricts root symbioses and shows like its target NSP2 a complex transcriptional regulation in *Medicago truncatula*. *BMC Plant Biol.* 14:199. 10.1186/s12870-014-0199-1 25928247 PMC4115173

[B37] Huang J. Yang M. Zhang X. (2016). The function of small RNAs in plant biotic stress response. *J. Integr. Plant Biol.* 58 312–327. 10.1111/jipb.12463 26748943

[B38] Jorge V. Dowkiw A. Faivre-Rampant P. Bastien C. (2005). Genetic architecture of qualitative and quantitative *Melampsora larici-populina* leaf rust resistance in hybrid poplar: genetic mapping and QTL detection. *New Phytol.* 167 113–127. 10.1111/j.1469-8137.2005.01424.x 15948835

[B39] Kastenmayer J. P. Ni L. Chu A. Kitchen L. E. Au W.-C. Yang H. (2006). Functional genomics of genes with small open reading frames (sORFs) in *S. cerevisiae*. *Genome Res.* 16 365–373. 10.1101/gr.4355406 16510898 PMC1415214

[B40] Kim D. Pertea G. Trapnell C. Pimentel H. Kelley R. Salzberg S. L. (2013). TopHat2: accurate alignment of transcriptomes in the presence of insertions, deletions and gene fusions. *Genome Biol.* 14:R36. 10.1186/gb-2013-14-4-r36 23618408 PMC4053844

[B41] Kloppholz S. Kuhn H. Requena N. (2011). A secreted fungal effector of *Glomus intraradices* promotes symbiotic biotrophy. *Curr. Biol.* 21 1204–1209. 10.1016/j.cub.2011.06.044 21757354

[B42] Koske R. Gemma J. (1989). A modified procedure for staining roots to detect VA mycorrhizas. *Mycol. Res.* 92 486–488. 10.1016/S0953-7562(89)80195-9

[B43] Kumari P. Sampath K. (2015). cncRNAs: Bi-functional RNAs with protein coding and non-coding functions. *Semin. Cell Dev. Biol.* 47–48, 40–51. 10.1016/j.semcdb.2015.10.024 26498036 PMC4683094

[B44] Labbé J. Jorge V. Kohler A. Vion P. Marçais B. Bastien C. (2011). Identification of quantitative trait loci affecting ectomycorrhizal symbiosis in an interspecific F1 poplar cross and differential expression of genes in ectomycorrhizas of the two parents: *Populus deltoides* and *Populus trichocarpa*. *Tree Genet. Genomes* 7 617–627. 10.1007/s11295-010-0361-3

[B45] Ladoukakis E. Pereira V. Magny E. G. Eyre-Walker A. Couso J. P. (2011). Hundreds of putatively functional small open reading frames in *Drosophila*. *Genome Biol.* 12:R118. 10.1186/gb-2011-12-11-r118 22118156 PMC3334604

[B46] Lanet E. Delannoy E. Sormani R. Floris M. Brodersen P. Crété P. (2009). Biochemical evidence for translational repression by *Arabidopsis* microRNAs. *Plant Cell* 21 1762–1768. 10.1105/tpc.108.063412 19531599 PMC2714937

[B47] Lauressergues D. Delaux P. M. Formey D. Lelandais-Brière C. Fort S. Cottaz S. (2012). The microRNA miR171h modulates arbuscular mycorrhizal colonization of *Medicago truncatula* by targeting NSP2. *Plant J.* 72 512–522. 10.1111/j.1365-313X.2012.05099.x 22775306

[B48] Lefebvre F. A. Lécuyer E. (2017). Small luggage for a long journey: transfer of vesicle-enclosed small RNA in interspecies communication. *Front. Microbiol.* 8:377. 10.3389/fmicb.2017.00377 28360889 PMC5352665

[B49] Li H.-M. Hu C.-S. Bai L. (2014). Genome-wide identification of coding small open reading frames: the unknown transcriptome. *J. Shanghai Jiaotong Univers.* 19 663–668. 10.1371/journal.pone.0152363 27018591 PMC4809551

[B50] Love M. I. Huber W. Anders S. (2014). Moderated estimation of fold change and dispersion for RNA-seq data with DESeq2. *Genome Biol.* 15:550. 10.1186/s13059-014-0550-8 25516281 PMC4302049

[B51] Ma J. Diedrich J. K. Jungreis I. Donaldson C. Vaughan J. Kellis M. (2016). Improved identification and analysis of small open reading frame encoded polypeptides. *Anal. Chem.* 88 3967–3975. 10.1021/acs.analchem.6b00191 27010111 PMC4939623

[B52] Mallory A. C. Vaucheret H. (2006). Functions of microRNAs and related small RNAs in plants. *Nat. Genet.* 38 S31–S36. 16736022 10.1038/ng1791

[B53] Martin F. Aerts A. Ahrén D. Brun A. Danchin E. Duchaussoy F. (2008). The genome of *Laccaria bicolor* provides insights into mycorrhizal symbiosis. *Nature* 452 88–92. 10.1038/nature06556 18322534

[B54] Martin F. M. Uroz S. Barker D. G. (2017). Ancestral alliances: plant mutualistic symbioses with fungi and bacteria. *Science* 356:eaad4501. 10.1126/science.aad4501 28546156

[B55] Mohanta T. K. Bae H. (2015). Functional genomics and signaling events in mycorrhizal symbiosis. *J. Plant Interact.* 10 21–40. 10.1080/17429145.2015.1005180

[B56] Molnar A. Melnyk C. Baulcombe D. C. (2011). Silencing signals in plants: a long journey for small RNAs. *Genome Biol.* 12:215. 10.1186/gb-2010-11-12-219 21235831 PMC3091295

[B57] Oyama M. Itagaki C. Hata H. Suzuki Y. Izumi T. Natsume T. (2004). Analysis of small human proteins reveals the translation of upstream open reading frames of mRNAs. *Genome Res.* 14 2048–2052. 10.1101/gr.2384604 15489325 PMC528919

[B58] Plett J. M. Daguerre Y. Wittulsky S. Vayssières A. Deveau A. Melton S. J. (2014). Effector MiSSP7 of the mutualistic fungus *Laccaria bicolor* stabilizes the *Populus* JAZ6 protein and represses jasmonic acid (JA) responsive genes. *Proc. Natl. Acad. Sci. U.S.A.* 111 8299–8304. 10.1073/pnas.1322671111 24847068 PMC4050555

[B59] Plett J. M. Kemppainen M. Kale S. D. Kohler A. Legué V. Brun A. (2011). A secreted effector protein of *Laccaria bicolor* is required for symbiosis development. *Curr. Biol.* 21 1197–1203. 10.1016/j.cub.2011.05.033 21757352

[B60] Plett J. M. Martin F. (2011). Blurred boundaries: lifestyle lessons from ectomycorrhizal fungal genomes. *Trends Genet.* 27 14–22. 10.1016/j.tig.2010.10.005 21112661

[B61] Plett J. M. Yin H. Mewalal R. Hu R. Li T. Ranjan P. (2017). *Populus trichocarpa* encodes small, effector-like secreted proteins that are highly induced during mutualistic symbiosis. *Sci. Rep.* 7:382. 10.1038/s41598-017-00400-8 28336910 PMC5428498

[B62] Powell C. L. (2018). “Field inoculation with VA mycorrhizal fungi,” in *VA Mycorrhiza*, eds Powell C. L. Bagyaraj D. J. (Boca Raton, FL: CRC Press), 205–222.

[B63] Ruiz-Ferrer V. Voinnet O. (2009). Roles of plant small RNAs in biotic stress responses. *Annu. Rev. Plant Biol.* 60 485–510. 10.1146/annurev.arplant.043008.092111 19519217

[B64] Ruiz-Orera J. Messeguer X. Subirana J. A. Alba M. M. (2014). Long non-coding RNAs as a source of new peptides. *eLife* 3:e03523. 10.7554/eLife.03523 25233276 PMC4359382

[B65] Scharf B. E. Hynes M. F. Alexandre G. M. (2016). Chemotaxis signaling systems in model beneficial plant–bacteria associations. *Plant Mol. Biol.* 90 549–559. 10.3389/fmicb.2016.01982 26797793

[B66] Shahid S. Kim G. Johnson N. R. Wafula E. Wang F. Coruh C. (2018). MicroRNAs from the parasitic plant *Cuscuta campestris* target host messenger RNAs. *Nature* 553 82–85. 10.1038/nature25027 29300014

[B67] Shell S. S. Wang J. Lapierre P. Mir M. Chase M. R. Pyle M. M. (2015). Leaderless transcripts and small proteins are common features of the mycobacterial translational landscape. *PLoS Genet.* 11:e1005641. 10.1371/journal.pgen.1005641 26536359 PMC4633059

[B68] Sjödin A. Street N. R. Sandberg G. Gustafsson P. Jansson S. (2009). The *Populus* Genome Integrative Explorer (PopGenIE): a new resource for exploring the *Populus* genome. *New Phytol.* 182 1013–1025. 10.1111/j.1469-8137.2009.02807.x 19383103

[B69] Smith S. E. Read D. J. (2008). *Mycorrhizal Symbiosis.* Cambridge, MA: Academic press.

[B70] Soneson C. Delorenzi M. (2013). A comparison of methods for differential expression analysis of RNA-seq data. *BMC Bioinformatics* 14:91. 10.1186/1471-2105-14-91 23497356 PMC3608160

[B71] Sukumar P. Legue V. Vayssieres A. Martin F. Tuskan G. A. Kalluri U. C. (2013). Involvement of auxin pathways in modulating root architecture during beneficial plant–microorganism interactions. *Plant Cell Environ.* 36 909–919. 10.1111/pce.12036 23145472

[B72] Supek F. Bošnjak M. Škunca N. Šmuc T. (2011). REVIGO summarizes and visualizes long lists of gene ontology terms. *PLoS One* 6:e21800. 10.1371/journal.pone.0021800 21789182 PMC3138752

[B73] Tagu D. Rampant P. F. Lapeyrie F. Frey-Klett P. Vion P. Villar M. (2001). Variation in the ability to form ectomycorrhizas in the F1 progeny of an interspecific poplar (*Populus* spp.) cross. *Mycorrhiza* 10 237–240. 10.1007/PL00009997

[B74] Trapnell C. Roberts A. Goff L. Pertea G. Kim D. Kelley D. R. (2012). Differential gene and transcript expression analysis of RNA-seq experiments with TopHat and Cufflinks. *Nat. Protoc.* 7 562–578. 10.1038/nprot.2012.016 22383036 PMC3334321

[B75] Ulveling D. Francastel C. Hubé F. (2011). When one is better than two: RNA with dual functions. *Biochimie* 93 633–644. 10.1016/j.biochi.2010.11.004 21111023

[B76] Vaucheret H. (2006). Post-transcriptional small RNA pathways in plants: mechanisms and regulations. *Genes Dev.* 20 759–771. 10.1101/gad.1410506 16600909

[B77] Wang M. Thomas N. Jin H. (2017). Cross-kingdom RNA trafficking and environmental RNAi for powerful innovative pre-and post-harvest plant protection. *Curr. Opin. Plant Biol.* 38 133–141. 10.1016/j.pbi.2017.05.003 28570950 PMC5720367

[B78] Wheeler D. L. Church D. M. Federhen S. Lash A. E. Madden T. L. Pontius J. U. (2003). Database resources of the national center for biotechnology. *Nucleic Acids Res.* 31 28–33. 10.1093/nar/gkg033 12519941 PMC165480

[B79] Wu P. Wu Y. Liu C.-C. Liu L.-W. Ma F.-F. Wu X.-Y. (2016). Identification of arbuscular mycorrhiza (AM)-responsive microRNAs in tomato. *Front. Plant Sci.* 7:429. 10.3389/fpls.2016.00429 27066061 PMC4814767

[B80] Yang X. Li L. (2011). miRDeep-P: a computational tool for analyzing the microRNA transcriptome in plants. *Bioinformatics* 27 2614–2615. 10.1093/bioinformatics/btr430 21775303

[B81] Yang X. Tschaplinski T. J. Hurst G. B. Jawdy S. Abraham P. E. Lankford P. K. (2011). Discovery and annotation of small proteins using genomics, proteomics, and computational approaches. *Genome Res.* 21 634–641. 10.1101/gr.109280.110 21367939 PMC3065711

[B82] Yi X. Zhang Z. Ling Y. Xu W. Su Z. (2015). PNRD: a plant non-coding RNA database. *Nucleic Acids Res.* 43 D982–D989. 10.1093/nar/gku1162 25398903 PMC4383960

[B83] Yin H. Fan Z. Li X. Wang J. Liu W. Wu B. (2016). Phylogenetic tree-informed microRNAome analysis uncovers conserved and lineage-specific miRNAs in *Camellia* during floral organ development. *J. Exp. Bot.* 67 2641–2653. 10.1093/jxb/erw095 26951373

[B84] Zhang Y.-C. Chen Y.-Q. (2013). Long noncoding RNAs: new regulators in plant development. *Biochem. Biophys. Res. Commun.* 436 111–114. 10.1016/j.bbrc.2013.05.086 23726911

