# Supplementary material for: Identification of Populus Small RNAs Responsive to Mutualistic Interactions With Mycorrhizal Fungi, Laccaria bicolor and Rhizophagus irregularis
Source: Front Microbiol. 2019 Mar 18;10:515. doi: 10.3389/fmicb.2019.00515 (PMC6431645; doi:10.3389/fmicb.2019.00515)
Supplement: Supplementary file 1 [file Table_1.DOCX]

Sequences of differentially expressed *Populus deltoides* transcripts in fungal treatments relative to control.

>Podel.CUFF.1650.1

GACAGATTCAAAGGACTTAAATTTAACAGTCAACTAAGTCCAAGATCATATGGGTTTGACAAATATGTCAGATCTAAGCCGCTTAAACTTAGCAGTCAGCTACGTACAAAACACTTGGACTTAGCGCTCTACTAAGTCTAAGGTCACATGGGTCTTGCAAGCATGCCAGACCCAAAATACTTGAGC

>Podel.CUFF.1732.1

ACTGGAGGCTTACATGGTCGTTAACTTCAGAGTCCATGGAATTAGTCGAGGTGCACGCAAGCTGGACCGGACACCCACATTAA

>Podel.CUFF.1989.1

AAAAAAAACCGGGTCTCGTTCGGGTTCGCCCGGGTCACCCGAGTTCCGGGTCGACCCGCCGGGTTGCCCTGGTTTGGTCGGGCT

>Podel.CUFF.2393.1

TATGGTTCGCTGTGGCTGCTG

>Podel.CUFF.2863.1

AAATACAACTTTCAACAACGACATCT

>Podel.CUFF.2992.1

AGAATTACTAAACACCCTTAAGCCAGTGGACAGACACAAACGACAAAATACAAAGGAAAAGGGATAAAAGCTTATATTTGGGGATGGCAGAAGGGCATTGAGATCAAAGAGGATAAGTTCTCCAGTGATGGGATTGGATGGTGATGAAGGATTGTGTGGATAAGAGCATAGTAATTAATTATTTACTAAAAAGTTCTTCAGTGACCTAATTCCGTGGCCCCACAAAATTATCATTATCCAAAAAACTATTTGATTAATTCAGTGTTGCTTAATCCCAGTTTGCTATTTCGTT

>Podel.CUFF.3137.1

TTATGACTGTTTGAGGGTCGTG

>Podel.CUFF.610.1

TTAACTTCAGAGTCTCGAGGGATTAATCGAGATGTGTGTAAGCTGACCCGGACAGCCACGATTACGAAAAAAAGAGCTAAAAATCCTAATCACTCTAACACTGTTTGTCTTGTGGTCCAACCGTGT

>Podel.CUFF.621.1

TGAAAATCCAGGTGAAGAAAG

>Podel.CUFF.889.1

GATTTGGTGGTTCTCATTTTTCATTCATCTGTGTGTTCGTTATCTGGTCTTCTATGAGACAGACGAGTGATTGTTTTCCT

>Podel.CUFF.90.1

AGCAAGCTCTATCCCAACTCATCTCAATGA

>Podel.CUFF.92.1

TTTAAAGAATCGATGGTTCACGTAGCAATGCAGTGAC

>Podel.CUFF.1036.1

AGTTTCAAAGAAACGGTTAGTTATGATTGAGAAAAAAACATCACTCTTACCGTATCTAATGTAGTTGAGCTTGCGACAACGAGTGATTCTGAGCGGTTAGGTGTAAGTTTATCATAACTAGATGAGGATTTGCCCTAGATAGATTACGTGAATGACCTAGCAGATACATTTATGGATCAGTGAGACAAGTTTAAGCTCTTGCGCCAGATTAGAATAACAGCTCAAAGGGCCATAATTTTTTATTCGACTGTTGGATCATA

>Podel.CUFF.1038.1

AATTCTCTTTCCCAAGTTCTTTT

>Podel.CUFF.133.1

AATGGTCTATTTCAATGAGATAATAATCCCACTATTTTTTTAACTAGTCGGAGAAATCAGCAATAAGGAGTTCAAATATAGCTTTATCTAGGGTTCCTATTTGAGTTTTGTAGGCGTTAGCTATC

>Podel.CUFF.134.1

AACAACATTGCCGACTTGAGATCCAGGATCTAGAGTCTAAAGGTCTGACCCAAAAATCCAGATCACCATGGTCATGTGCAGCCTTTTTATGTGAATAAAAACTCTGAAATTGAAAGGCGCACACC

>Podel.CUFF.1370.1

AAGCTGATCTCGAGCGACGCGACCTCCAACTGACCGAGTGGCGATTCCACATCTACTCCAAGGAAAATGGCGTCCGAAAACTCCTGTAAAGATTTGAGGGCGATCCAACGGTCGGATCAAAAGTTATGGCCGTTTCAAACCCGTAAGCTGATCTCAAGCGACGCGACCTCCACCTGACCGAGTGGCGATTCCACATCTACTCCAAGGAAAA

>Podel.CUFF.139.1

ATCTTTTTTGTTTGGGATTGGATATCAAATCCATTTTAAAGCAGAATTTAAGGGTTTGTAGTTGATCACAAATCTTGGTGTGCCTCTTTCAATATCAGAGTTTTTATTCACAGAGAAGGCTGCACACGACCATGGTGATCTGGACTTTTGAATAAGACCTTTAGACTCCATATCTAGGATTTCAAGTCTGCAATGCTGTTTGAGATCAACCTTCATTTGAATAGGTCTTGCCTTTATAGGTATATGTTTTTTCGGAAAAAGTATGTTCATAAGCAAGATCAGCAACATGTTGTTTTCTTTTCCAGAAGGCATTGGGTAAGTCTGAACAGATTTGTTGTTCAATCTTACTTTGTAAATCAGTGATTCTCTTTTGTAAAACCGAGTTTTGTAATTATTTATTGATACGACAAAATGAAACATGGTTT

>Podel.CUFF.142.1

TAAGAGCAACAAGAATAGATCCTTCAAGCATATGGTAATTATGGGTCTTGATTTGTAATAACATGCTTTGTAAAATGTTTTTATCTTTGAGGGATATAGTAAGGTTTGAATAGCAGTCAAAAGATACTGGACCACTACAGAGGCTAGACTTAATGGAATTTAGGAGTGATTCTTGGAAGTTGTGGAATCTAGCATCTCGGAGTATATCTAGAATGGAGGTATTTAAGCCTTCTTTTGTAAGTAGTTTTATTTCGACTTGAACTAGACCGAAATGAATGTATTTGTAGTTTCTGGCTAGGTGTTTTTGTAGTGATTTTTTTGGAGAGGAGTTGGATAGTCTC

>Podel.CUFF.145.1

ATTCCACTTGAAGAGGCAGAACTGGTTACAACCACAACAAGTTTTTCTCCAGACGAATCATATTATTCCTCTGTACTCACAAACTGTATCATTTTATTCCCTGCTTTGTATTCAAAACCTGTATCAATTCCAAAACTGTAACCCATATCTCCTGTAAATTATCCTCTGCTT

>Podel.CUFF.1475.1

GGTGGCTGTTTTTTAGTGGTGAGAATTGCACC

>Podel.CUFF.149.1

AACCTAAGACCAAGAATTCATCCTTGAGGTCATCAAAAGACTTGATGATCCTCAGCTCCAAAAAACCTATTTAGATAAACTTTTGAAAGATTTCAACAAACCAGAACACCTCTATCTAGCCCTCCAAACCGCTTTGTATTACCCTCCATTAGTATCAATATCTACGACTTTACAAAGATCCTTAACAAGAAGAAATCCAATACCTCAGTTACCATCCCAGAATTACATTCTGAGATCAAAATCCTCAAATTTGAGTTACAAACCCTAAAACACGCTGAACAGAAAGACTTTGTCATCTTACAAGATCTTCTATCCAAAATTGCGACCCAGTCTGACACTGAGTCTTAATCGGAAGACCAAACTGATAAGTCTGCATTACGCCATGCACTTTTCAAACATTGAGCATATTCCTGATGATTTCTTGAATGTGTTGACCCAAATATCTTCCAAA

>Podel.CUFF.1615.1

AAACAGTACATGTTGCTGCCCAGGATCGAACTGGGGACCTTTAGTGTGTAAGACTAACGTGATAACCACTACACCACAACAAC

>Podel.CUFF.1658.1

AAGACTAAGTAACTTGTTCTCCCTGTAATCTTAGGTTCGAGCTCTATGGTTGTTAATATGATGGTCATTGAAGGCTTATATGGTCATTAACTTCAGAATTCATGAAATTAGTCGAGTTATGCCCAAACTAACCTAGACACC

>Podel.CUFF.1676.1

ATCACGCCCGGTGGGATTCGAACCCACAGTCGCCTGATTAGAAGTCAGACGCCTTATCCATTAGGCCACGGGCGCTTTTT

>Podel.CUFF.1692.1

AGGACATAATAAAACAATACGAGTCTACTTGGATTAACTT

>Podel.CUFF.1714.1

AACAAAGCTGGAATAGCTCAGTTGGCTAGAGCGTGTGGCTGTTAACCACAAGGTCGGAGGTTCAAGCCCTCCTTCTAGCGCCT

>Podel.CUFF.1757.1

GGTGAAATTCTAGGATTGACC

>Podel.CUFF.1796.1

TTTATCCCTCGAATATTGATCGAATATGTATGAAATATGATGGTTATATGTTTCATTCATATTCGGTCAACGTTCGAGTGA

>Podel.CUFF.1802.1

ATCTCTAACGATGATCAACTCGAAACTTTCGTTCTCCTGAGCAAATTCATGCTGAAAATTGTGGAGACCTGACCTGAAGAGCTGTAAAAATGATCTTCAATGTCCATTCTGTTTT

>Podel.CUFF.1930.1

CATGAAATACCTAACTGCAATGGAGGAATTGTCTATTTACCATTGTGACAAGCTTGATTTGATGATAATAGAAGAAGAGAAAGAGGAAAAAATTCAACCAATTTCCCTTCAGATTGTAACGTTTCCAAGGTTACCAGCAACACTTGCATTACCAAAACAGCTTCTTCA

>Podel.CUFF.196.1

CTAGTTTACATGTATCTCGACTAATTTCATGGATTTTGAAATTAACAACCATGTAAACTTCCAGTGACCCTAAAATTTATAAGACTCGAACCAGTAAT

>Podel.CUFF.1983.1

AAGATTTATATAGGTGTCTGGGCCAGTTTATACTGTATCTTTACTAATCTCACGAACTCTGAAGTTAACGATCATGTAAGCCTCAAGTGGCCCTGAAATTTGTGGAACTCAAACTGGTGACCTCTAGAGAGTAAATCTAGGACCTGACCAATTAAGTT

>Podel.CUFF.2062.1

TAGCCCCCGGCGAGGATCGAACTCGCGACCTTTCGCTTACGAAGCGAACGCACTACCACTATGCTACGGAGGC

>Podel.CUFF.2100.1

TCTGGTTCTTCCACCAATGGTCCTTGCACACTATTATTATGTTATATATGTATGTCAGGAAACTAATCATACAGAACCTAATATTATGGGATGATTATGTCAGGAGTAGGAGTTCTAATAGTTATGATGATAGAGCTAAATTGTTATGGAGATTAGATTGGGCTCAAAAGCTTGTACCTCTAGCACATTAAGTTGCAAGTTCTCAAGTTTTCTTGTATTAGGTGCAGTAGCAAAGAAACTTCATGCTAA

>Podel.CUFF.2102.1

AGAATCAACTTCAACATAGTCGTACCATAACTCCAAGTCAATCCATATAAGGTTATGCAAGAGCAATCACCTTCCTAAGCAAGTCTAGATGTCAGTAGCATTTGATTTAATTTTTAGCAAAATGATGTCATATTAGCCAAACTTCTTTCAGTACATTGCTTATGTATAGTAGCATGTGACTAATAATCAAATACATGAGGTTGAATTAACATAAGTGCTTAGTTTTTTGCTTGTACTAATCAATTGGGAGTTGTCATCAAGGTAGTTGAGACAACAAATTCTATTAACCAA

>Podel.CUFF.2104.1

AAGTGATAGCTTATGGATTGTATAATTGTGTAGTTGCCTATTAATTTGAAGGGACTGGGGGTATCCATAGAAATAATACAGTATTAACATCCATGAAAAACCTAGTTGGCCTTAAAAATTTATTTTGAATGCAGGTAAGATGCAAGTGGGGAATAACCTAGGTTGAAACATGTATTAGTTTAAAGTCATCACATCTACTAATTAGCATGTAATTTCTTTGCTACTGAACCCAATGCAATAACACTTAGGAACTTGCAACTTAATGTACTAGAGGCACAAGCTTTTGAGCCCAATCTAATTGGGCTTCTCCATAACAATTTAGCTCTCTCTTCCCTACAAGATAACTATTAGAACTCTTACTCCTGACTTAATCATCCCATAATATTAGGTTCTTTATAATTAGTTTCGTGACATGCATATATAGCATTATAATAGTGTGCAAGGACCATTAATGAAAGAACCAGA

>Podel.CUFF.2130.1

AAATATCAGTTCCATCATGAGGGCTCAGTAAACAGCA

>Podel.CUFF.2172.1

CTTGTAGCCTGGTGGTTCGAGTTACACACGCTCAATCCTAAAGGTTATGGGTTCGAGGCCGGCACAAAGAGAAGGAAAAGGAAACCATATATACTTCGAAAAGAGGTATTATAA

>Podel.CUFF.2184.1

ATGCCGACTTCCCTTTTCTACT

>Podel.CUFF.2231.1

TTCTTCACTCTCTTACATTCTCAGCTCTATATTGAGTCTATTTATAGACCAAGCTAGGAGTAATGACCATAAATCACGGTAGGATTTATGCCACCATTTATGCCTTAAATTGAGCCTCCACCTTGTGCTAAGTGGAGATGAGTTGTTCTCACTAACATATAGTGGAGGTAGGGCATTGTTTGTCTCCATTTTA

>Podel.CUFF.2373.1

GAGCTCTCCCCACTCCATGCCTGAAAGGAGTTCGATGGTAGACCATGGCTGCTAGTTCATGAATACCCTTGGGTGCGCAGAATTAGAAACGGTGCAGGCGAAGTTGCGCAGGCTAAGGGTCTGCATGACCTAGGAGACGTGGTTACCCTGACCCTTTTTGTATTGGAGTGAAGGGAGCTCGATGG

>Podel.CUFF.2447.1

TTTAGAAACCAACAAAATTGAACCAA

>Podel.CUFF.2657.1

CAAATTTTCTCCATCTTCAATAGCCACAGCGCACAACAATTGCACTGCCAACAAAATTATAAACAAGATAAGAGCCACTTTTGCTTCAACTGAACGAACCATTAAGGAACCCATCCAACCTCAATGGTCAGAGAAAGAAGCTT

>Podel.CUFF.2663.1

TGTTGGCTTCAGCGAGTTAGTTCTTCATGTGCCTGTCTTCCCCATCA

>Podel.CUFF.2671.1

ATGGCCCATGCAGGTTTCGAACCTGCGACTTCGCGTTATTAGCACGACGCTCTAACCAACTGAGCTAATAGGCCTTG

>Podel.CUFF.2685.1

ACAAGAGCCCTTATAGCTCAGTGGTAGAGCGTCAGTCTTGTAAACTGAAGGTCCGTAGTTCGATCCTGCGTGAGGGCATTT

>Podel.CUFF.2718.1

ATTGTCAAGGCTAGAGGATCCACTAATGGTATTTCAAACAAGTATAGTATAAACTCTTTTTATTACTCAACTGGAAACCACACATAAAGGAGGTTCCAATCGGATT

>Podel.CUFF.279.1

GAAATTGATGAATAAAGCAGGTACTTATGATAGCTTGTCATATCTAGATGTTCTCCTGGTGCAGCATATATTGATGTTGTGCTGAGCTTCTATGCTATGTCTACATGACTAAACCAACTATCTGCCAACTGATTCCA

>Podel.CUFF.2820.1

GTTCCTTTGTGTTCTGCATGGGATCGCATGAGCTTGCATGCGTTGATCAGTTCTTGGTATTTCTGTTTGGCCTTGCATGTTGAGGGCTGGACGTGCATGGTTCCTCGTTTCTGCATGGTCTTTCATTTAATTTGGCTGGGTGGGAGCTTCTGATTGGCTATACTTCGTTCTCTGGATGT

>Podel.CUFF.2882.1

TACATCTCAACTAATCTCACGGGTTTTGAAATTAACGACCATATAAGTCTCCAGTGATCCTGAGATTTATGAGACTCGAACTGGTGATCTCTAAA

>Podel.CUFF.2933.1

GAGACTCACGAAGAAGTAAGGCATGGTTCCTAGGGTGGAACAGAGGGCAGATGCAACTCCTGGATGAGTAGGCTCTTGGAATAGTGGTGAGCTCGTGATCATATTGAGATACT

>Podel.CUFF.3016.1

ACATTTAAACTATCCTTCATGTATCCTAAACAGAAATCCTACTTACGTTTTTTTTAGAAATCATTTTTATAATTTTAAAACATTTAAACTATCCTTCAAGTATC

>Podel.CUFF.3036.1

TCCAGCAATGGACAGTCTCTTTCCGAAATTAGCTTACTTCTAGTGATTCTTACCTGGGGGCTACATCCTTTTATCATTATAAAAATAAGAAAGTTCGATCCATTAGGTTCTTCGATTTGTCTTATACCTTTGGCACCCTTGGTTAAAAGGGGCAGATAAATAGTTTAGGCGAAATAGAATCAAGTCTCCTTACTAGGTGAAGCTACAACAGATAAATTAGAGGAAATCCATTCCATTCAAAA

>Podel.CUFF.3067.1

TTGCTGGTCCCAAGTCCTTTATATCAAACTCCCTAGCCAACTGTGCCTTCAATTCTTGGACTCGATCTTTGTTGGGGCCTATTACCAACATTTCA

>Podel.CUFF.3113.1

AGTCTGTTCCCTTTGATTGTTCCAGGGAGTCTGTTCCCTTTGCTTTTCTATTCAGCAGGCTTGTCTTGCTTATGGTTTGTACAAGGCAATCTGTTCCCTTTGTTTTTGTTCAAGGGAGTCTTTTCCCTTTGACTGTTCTATTTCAGCAAGCTTGTCTTGCTTATGGTTTGTTCAAGGGAGCATGTTCCCTCTGATATCTGTTCAGCAAGCTTGTCTTGTTTATGTTTTGTTCAAGGGACTTCGTTCCCTTTTTCTTGTATTGATGGTCTCCAGCGCATTTTTGATCTATAATATTCTTACATTTGATCAAAAAAAAGAGTTACTCCTTCTGTATGAAGCTTTTCCCTCGTGTTTTTGATCTCCTACCGTTGTTTGCTCTCTTCCTTCATTTGTTCTGTTGATTTTCTGTTTATGGCGAACAAAAAGAAGGCTGGGTCCCATAAATCTAGGCTTAAACAACAATCTTTGGCTAATTCTCAGCTTTTGAATGAATGCATTGTTCTTGCTTTAGCGGCCCCGTCTCAGTCTCCTACTGTTCATGCTACTGTTCCCTCAAATTCAAATTCAAATCCATGCCCGCCTGTCCCCTCCACTTCACATGGCTTTCTACCTCAGATCCCTGTTGATCCGTCCCTGGTATCAACCATGTGTTTGTGGAAGATTATTCTGATGATGATGCTCTTGATGACGAGGAAGTGGATTTTGACTCTTCTGGGGAGGACTATGCAGGAGGGTCTAAATTTTTCACCTCTTCAGTATTTGAGGCCCCTTCCGTCCCTGTTGCTTCCCCTATAGGTGCTCAGTCAGTCTCTGTTGCTTCCCCTGCATGCACCTTTCTAGATCTTGTTGCTTTCCCAGTACGTACCTTGTCAACTCCTGTTGCTCCTGCGGAGGAGAATGTCCCTTTGGCTGCTACAGTAAGTTAAACTCCTAATCCTTTTGAGCCTCCACCTAATGCTTGGCGTAATCTGTTCGCCCTTAATCGCATCAATAGTTTCCCCAAGCTCCTCATTACTCTGTTTTTACTGAAACTAGAGGTTGTAATTTGATTGATGATGATTTAGACAATAAATATGGCCTTTGGAAGCTGAGCCTTGTCGGCTACATTGATTTTCGTTCACCAGGCTTTAAAGCCTTGCAGAATCTCATTGTTAACTCCTGGCACTGTGAAGCATCCTTGACAATCCATGCGTCTGGTTGGTTGATTTTCCAATTTGCTAATGAAGAAGATAAATTGAATGTTTTATTTGGTGGCCCTTATCTTGTCTATGGCAGACCCCTAATTCTTAGGGCAATGCTAAAATACTTTGATTTCTCTTCTTTTGATATGCATACTGTTTCGGTCTGGGTTAAATTCCCTAATCTTCCTCTCAAGTGCTGGTCTCTCAAATGTTTATCTAAAATCACTAGTGTCCTTGGTAAACCTATTCAAAGTGACATGCTCACTTCTTCCATGTCCAAACTATCGTATGCCAGGGTGTTGGTGGAAGTTAACTTACTCTTTGATTTGCCTTACTCAATTGAGGCTACTCTGCCTAATGGCAGTATACTTCATCAGCAAGTTGTTTATGAGACCCTCCCACGCTTTTGCAAGCATTGCAGGACTCTTGGTCACATTACTTCAACTTGCACTCAATCTCAGCCTACCAATGTTTCTACCACACAACAGGCCCATGACTCTGTTGCTCCATCTCTAATGGCAGGACTAGTGTTTTCAACCGTTTGGGCCTCAAGGTGGCGCTGTTGTAGTTGAAAGTCCAGAGGCAAACCTGCCTGCTGACTGTGGACCTAATCCTCTGCCGGTTGAAGCCGGGCTTGTTTCGGCAAAATGGTGTTGTTGTGCCTACCTCTGGTGCTTGGGTAGTTGTTAGGAACAAAAATGTTAAGCGCAAGCCTCCCCCCTCAAGGACTGCTTCTGAATCTCCGTCTTGCTTGGCCCAAGAGAATCCTCATTTGGTTCACAAGGAATGTCAGCCTACTCCACCTCTCCCTGCTGCCCGCAACACCTCCGCTACAATTTTAGCTGGTCATAGGACTGACAAGGGCAAATCTGTGGTTTCAGGCGCATCAGGTCATTGCCCACCTAGTCCAACCGGTTGCCCATAAGGAGAAGGGCTCAGTAACACATGGGTGGTGTCCTTGGCAGGGGCGAAGGGTTACTTACCACCCCCTCCTCTTTATGTTAATTCGCAGTTGGAATATCAGAGGGTTAAACAGCCCTCTGAAGCAACATGAGGTTGGCAACCTCATGCGGAAGAATAAATTAGATGTTTGTGGTCTTTTGGAGACCAAATTGGTTTCGTTTAAAATGACTTCGATGCAAAAATTTCGATTGAAAAAATGGAAAGTTTTATCCAATGCAGCTGCGGCCTCAACAGCTAGAATAGTTGTTCTCTGGAACCCTGCTACGGTCAATGTTGACTTAATTGATTTTTCGGCTCAAGGCCTCCATGTTCTAATATATAGCTTGGTTCATCAGTTCAGATTTTATGCTTCTTTTGTTTATGGGCTTAACACTGTCACTGCTAAGAGATCTCTTTGGACTGACTTGAGGAACTGGAGTCCTAATTCCCCTTGGCTCATTTTAGGGGACTTCAATTCTCTTCTTTCTCAAGCAGACAAGCATCATTTTAGGGGACCTCAGGGCCGGAAATTAATGCCAGCAAATCCTTCATATTTTTTGGCAGCATATCAGCCAATATGAAGCATCTTCTTCTATCAAATACTAGGTTTGTTGAAGGGTCCTTTCCTTTTCGTTACCTTGGTGTACCCCTCAACCCTCACAGACTCCTTGCCAGTCAATACTCTCCTCTTCTCCATAAGCTTGAGTCGGCAATACAATGTTGGCTAGGGAAGCACCTCAGCTATGCTGGTAGAGTGGATCTGCTTAAGTCTGTTTTATATGGCATGGTTCAATTCTGGCTAAACATTTTTCCTGTTCCTGATATTGTGCTTAAACAAATCATTTGTATATGCCGTAATTTCTTGTGGACTGGTTCTGTCTCAAGGAACAAATCTGCATTGGTCGCTTGGAGCATGGTTTCTCCCTAAGAATGAAGGTGGATTGGGTCTTTTTGACATTAGAGCTCGTAATAACAGTTTTCTTGCTAAACTACTCTGAAATATTCATCTTAAAGCTGACTCCATTTGGATTTAATGGGTTCATCACTACTACTTACACGCTCAATCCATTTGGGACTCTACAGCTCACCCATGTCCATCTCCTTTGTGGAAATCCATTCTTAACTTTCGGGACAAACTTGTTGATAAGGGTGACAGCCAGTCTCAAACTCTTTCGTTGTTGGAAAGCTGGAGCTACTCTGTTGGCCCTTTCACAGCTCATGCTTATGATTTCTTGCGGTTTCGAAGCACTCCAGTGCTTTGGAAAAAGGTTGTTTGGGAGTCATGACCCATGCCTAGATACAGTTTTACTCTATGGTTGGCGGTTTTGGGAAGGCTGCGTACAAGAGATAGGCTCTGGTTCCTTCAAATAGACCCATCATGTGTTTTTTGCCAAGTGGATGAGTCGTCCCACAACCACCTGTTCTTTAGTTGCCATTGGACCTCTCTTCTCTAGCAGTTGATTAGTCCTGGCTGCGTATCACCAGGCGCATGTCAACCATAAGAAGTGCCATTCAGGGGCCGTGTCGGGGTGGAAACAATGCTGATGGAAGAATGAGGCGTGTCTCTCTTGGTATCCTTGTTTATATTATCTGGGAGGAGAGGAACAAAAGAATTTTTTACAGCACATGCAGCTCTATTGCTTCTCTCTTTCGCAAATTCCAGACTCTATTTTTCATGGTTTTCCATTTCCACGACAATGATCATTTTTCTCTCCATGTTGGCTGCTGACTAGTCTTTTGTCTTATCTTATTCGCTTGGGTTGTTCCGGTTGTGCTTTTCTGGTTGTGCGCTGATGGTTTGTTCGGTGGGTTCTTCTTTGTTTGTGCAAGTTGTTATTTATGGTCTCTTTGGCTAGAGCTATTTGCTTCTTTGTGTTCTGCATGGATCGCATGAGCTTGCATGCCTTTTCACATTCTTTTATTTTGATCCATTCTTGGTATTTCTGTTAGGCCTTGCATGTTGAGGGCTGGACGTGCATGGTTCCTCATTTCTGCATGGGTGAGCTGCTGTCCACTGTTCTTGGTTGTTAGTGCTTTAATTTGCTTTGGCTGTAATTTGGATGGTCCTCCTCTCTGCATGGTGCCTTCAACATCGCTTTGCTAAGCTGCTGGGTTGTAATACGTGGTTTAGGCATTCATGGGCTTCTGTAAGGTTTCTAGTCTCTGCATGGTCTTTCATTTAATTTGGATGGGTGGGAGCTTCTGATTGGCTATACTTCGTTCTCTGGATGTGATGATGAAAGGGCTCGGCATGCACTTGCATGTGGCCTTATTTGCTTGGTGCTCCAACTGCTGGTCGCTTTTGTTGACACTGGTCCTGTGCTTTTTGGCTGATCTTTAGACATCTGTCTACCTTCTCTCGTGTTCATTGTTCCTTGTATCTGCATGGATGAGCTGATGCCACTTGTTCTTGGCTGCTAGTGCATTATTGTGTTCTGGCTGGAACCTGGTTGGGCTGTCTCTGTCTCTGCAAGGATGGCTTTCTACGTCTCTGTATGGATGGGTATGCTTTGCATGGATGGCTGGCAATTGTCTCTACATGGGCGGCAAAAAGTTCTTCGTCCCTGCATGATAAGCCTCCAAGATTGCCTCTGTTATGGATGTTGTTGCATGTGGTTCCTTACCACATGGGGGGATCCTCATGGTGGCTTGGCTTTTCATTTATTGTTGTAGCCTCTCTTAAATGCTTTATACTTTGTCTATCGCTGTTATGTGCACTCCCCATTGGCTGGTTCCACTTATCTCCATTTGTTTGCTTGAGGTCCCTTTCCACGTGGGGGTATCTTTATGGTGGCTTGTTGATTCTTTATGGTTCCAATCGTCTGGCACCATTCTTTGTTTGTGTGGATTTGCTGCATGGCTTCCTCTCATGCTCATCGACTTGTCTGCTCTTCGCTCCAGCTTGTGGGTTCTTGGCTCTGGTCTCTCCTGCTTAGAGTTTCATTCATACTTTTGTCCTGTAGCTGTAGCTGATTCATCCTCGTCGGCTTGCTGCTTGATATCTTGGGTGTTCATCTCTACAGCTTAGCTCTTTGGTCTTTCATCACTGTTGTTTTGTTTTTCAGGATGTTCCTTTGTCCTGTCTTTGCTTTGTGTTAGGTTGGGAGCCTTGTAATTTTTGGTTCCTCTCATAACCATTGTCCATTTTTTCACTGTTATTATTTTTCAGTGGTTTCCTGTTTTGGTCTCTAAGGGAGCCTGTTCCTTTTCTGATCATCCTTGTATCTGTTCTTGCTGGCTCGCCAGCAATGTATATTTTTTT

>Podel.CUFF.358.1

CAGCATCAGAGTCATGGAAGTATGAGGTTAAACACTGCTCGCGTTAAAATAAACGCGTGCTTCTCATAATCCGGTATAATCCTCGTCATTTGC

>Podel.CUFF.387.1

TTGGTTCCAAATATTGTAATG

>Podel.CUFF.391.1

CTTTGGGCAAACAACAGTTTGTCTTTCATGATGAAAGATAATAATTTTACCTCATTGAGCAATCTTGCTCACTGATGGTGTGTAAGCTTTTTGTAATTAAAAGTATGTGAGGACTCTTATAATTAATTCTGACCTTGCAATCTTTATACAAGGTTCTTGTTTTCCTTAGAAATGTACCAAGGGTCTGAGCTCTAAATTCTGCTAGAACTGTGTAGAGATCCGGTGTGAGGCTGGATTTAACTTCTGTGCAGGGACGAACATCCTGTCTTCGAAACAATCCTAGCTTAATAAAATGGTAAGCAGGATAATTTTTGAGGCTGTCTGTTTCATCCTCTGTTTCTTC

>Podel.CUFF.478.1

CACCATCACCGGCCAGCTTGACAAGACCCGAATCAGACCCTCCAGCAGCAGCCTGAAC

>Podel.CUFF.490.1

CAAAAGTGGTTCCATGGTCTAGTGGTAAGGACATTGGACTCTGAATCCAGTAACCCGAGTTCAAATCTCGGTGGAACCTACTTTTTCTTCTAATGTTGCCT

>Podel.CUFF.646.1

CTAGATATGAACTCGGGTTACTGGATTCAAGTA

>Podel.CUFF.709.1

ATTTTGCAAAGAATGATTATTAGCTTAATCATAAATCTTCATTGTTCATGTTCCAGTACAAAACTTCTTTTCAATAATTTTTTCGAATATCCATCTATTTACAAACCATATTCATACATCAAAGAATTAAAATCTTTTAAGATTCTTACACAACACTATATTCCATTTGATTGCACAAAGAAACCAAATATTCGTAATGTT

>Podel.CUFF.830.1

AACAAATGGTCCCGTGGTCTAGTGGTGAGGACATTGGACTCTGAATCCAGTAACCCGAGTTCAAATCTCGGCGGGACCTCTT

>Podel.CUFF.897.1

ACTCGCACGTAGTTCTTCCTGGGAATCCATTTTGCAGTTTCCAGGATTTTATTTGTGCGTATAGCAAACTGAATACTATCCTTTCTGCTTGAGCCTTCTCCCTGATATTTTCATGGAGCTGTGCTCCAATGTTATTGGATTACAATTTT

>Podel.01G180400.1.v2.1

GCCTTTGTAGTAACTCGAAAAAACTTTACAGCAATCATGGAGGCCATGATCAGTACCCATAGTTCCTTTCTTTTATTTTTTTATTAATCACGAACTACGTACCCCTCGTTGTCTCGCACGAACAAAATATAAATAAGTGGTAGTTGAGTGCCTTCTCTCCCACTTGGAATGTTAGATTCACAGTAGTACTGACCGAAACAGCAACCCGGAGAAGTGAAAACCTTCCCCTTTTCTCCTCTTCGTCTCTCTTCTCTAACACACACTCGCAGAAAAAGACAAGCGTATTAATGGCTACTATGAAGAAAGAGTCGGTAAAGCCCGTGGAAACCATGCCCAACAAGGGAGCATGGACAGCTGAAGAAGATAGGAAACTGGCTGAGGTTATTGCAACTCATGGTGCCAAAAGATGGAGGACAATCGCATCCAAAGCAGCTCTCAATAGGTGTGGTAAGAGTTGCAGGCTAAGATGGTTGAATTATTTAAGACCAAACATCAAGAGAGGCAATATATCTGACCAAGAAGAGGACCTCATACTTAGGCTTCATAAACTACTAGGAAACAGGTGGTCACTGATTGCCGGAAGATTACCGGGTCGAACAGATAATGAGATCAAGAACTACTGGAATTCTCATTTGAGCAAGAAAATAAACAAAAAAGGGAAACAGATTGAAGCTTCAAATAGAGAATGCCAGACAATTGAGAAAAAGACCATGGAGATCAGTTTGGAGTTGAAAGAAGATAATAAGCCACATAATGGAAGTGAAGAAGGCTCAAATGTAAATTTTAATATAGACGGTCTCTTTGATTTCACCGATGAAGATACTTTGAACATGGAATGGATGAGCCGATTCCTTGAAATGGACGAGGTTTAAGTTCTGTTTTGTCATGAAAGTGTGTAAACTTGCTTCTCTCTTGATTTTAGTGCTGTAAATTATAGTGTAGCTG

>Podel.01G445900.1.v2.1

ATGAACGCACCTGAATGGGGACGTGCCTTGATTGGATGCTTGGCTGCGATAGGTGCTGTAGCTATTCAACCTATTAATGCTTACTGTGTGGGATCACTTGTATCAAAATACTTCTGCAGTGACAAATCTGCTGTAAAAAATAAGTCCAACATATCGGCATTGATTTCTGTAGTTATTGGTGCTCTCAACTTCATCACCAGCCTCCTTCAACATTATAATTTTGCAGTTATGGGTGAGAGGTTGACAAAAAGAGTACGCAAGAAACTTCAAGCGAAGCTGATGACTTATGAGATGGGTTGGTTTGATGATGATGAGAATACAAGTGCAGCAAGTTGTGCAAGGCTAGCCACTGAAGCCAGCATTGTTCGGTCCCTTGTTGGGGATAGGATGTCATTGCTAGAACAAACTTTCTTTGGTTCTGTCTTTGCATATGCACTAGGACTTGTTCTCACATGGAGGATAACCCTTGTTGTGATTGATTAA

>Podel.05G157600.1.v2.1

CCGCCAAATCGAAAGTAGACCCATCATGAAGAATTGGCTATCTGACCACAACCGTTAGATTGGAGATAGAATCCTGCCCAGCTGCATCTTTCAGCACCACCAAAGAAGAACCCAATAATCTTTTATCATAAAATATCCATACAGCACATATGGTCCTTATTATATCACCCGTCCCTCCTTTCATCCATGGTTGTATAAAACGACAAGCCTACTCTATCTTCACGCCATTAAGATACAAACCGATCCGACAAGAGTCCAAAGAAGTAGCTAGTTCCAAGGAAAAGAGACTAGATGGGTGTTGTAGAAGAAGCTCATAACGCGAAGATCTTGGGTTCAGGACAGCAAGTGATAGTTTTGGCTCATGGGTTTGGAACAGATCAGTCTGTCTGGAAGCACTTGGTTCCCCACCTTGTTGATGAGTACACTGTTATTTTGTATGATAACATGGGAGCTGGTACTACAAATCCAGATTACTTTGATTTCAGTAGGTACTCTACCCTCGAAGGTTTTGCTTATGATTTACTTGCCATTTTAGAGGAGCTGCATGTTGAGTCTTGTATTTTTGTTGGTCACTCCGTTTCTGGCATGGTTGGTGTTATTGCCTCCATTAGTCGCCCTGATCTCTTCTCTAAAATTGTCATGCTTTCTGCTTCTCCAAGGTACTTGAATGATGTTGATTATTATGGAGGATTCGAGCAGGAAGATTTAGACCAATTATTTGAAGCAATGCAAAACAATTACAAAGCATGGTGTTCTGGTTTTGCCCCACTAGCCGTGGGTGGAGACATGGATTCAGTAGCCGTGCAAGAATTCAGCCGCACACTCTTCAATATGAGACCAGACATAGCCCTTAGCGTGGCACAGACCATCTTCCACAGTGACATGAGGGCAATCCTACATATGGTCACAGTCCCCTGTCACATCCTGCAGAGCATGAAGGACTTGGCTGTGCCTGTGGTTGCCTCTGAACATTTGCACCAAAATCTTGGTGGTGAGTCCATTGTTGAAGTCATGTCATCTGATGGTCACCTGCCTCAGTTGAGCTCTCCCGACATTGTGATCCCTGTGCTTCTTAAGCACATTCGTTATAATATCGCTGCGTAATTTGTACTGGCAAACTCTTGTTTCCCTGCAGATTGTGTTTGCTTCTTCTTCTTTTCTTTTTTTTTTTATAGTTAATCTGGAAGTTAGTGAATGGTTGTACCATGACTGTAACGTCCCTTGTGGTTTGGCTGTGTCAAGTTGTGATTACTCATCATGTAATAAGATTCAAGAGAACATGGAGTTCGAATAGGCCTTTTTTC

>Podel.07G087100.1.v2.1

ATGGATGATGATGATAGATCCAAAGGCTTGTTTCTCAAGAACATTCGCAGTGGCCGCCTGAGAGCTTTCGCCTTTCGCCCAAGGCTTGAGAGCTTCTGCTGCATCTTTCCTTCCTTGGATAGTTTGATCCAGTTTTGCGATTATTTTGCAAATTTACACAATGACCCAGCTGCTTGA

>Podel.08G078000.1.v2.1

GTGGACCTTTGTGTATCTTCTCCATCTTGGGTCGTAGTGGTTGTTTTTCTCTCCTAGCCTAAGCAGCCATCACGGTGCCCATATTCAGGCTAGTTTCTGATCATGTTTGGGGTGCCCAGATTTACCACCACTTATCAAGTGAAATGAGAAGATGAAATCCGAACTTAGCCAACAGTTTGATGAAAATACCACGCTAATTACCAATGGGCCTATTGGTTCCAGTCGTAGGTTACTCTTGCAGGCGAGAGGTGCTGGGTTCAACTTGGGGGTGGAGTTGAACCTTACCTTCCCGACAACCCGAAATTGTTCTAGTTTCGGGTTGGAGAGCACTGAGAGTTCTTGAATCAGTAAATGATTGGTGGGATCTGGCCACCTGGGAAATGTACTTTGATTCAATTGGGCTTTTTTTTACTGAATTTTGATGAACTGTGGCTGGTTGTTTTAAAACATCGTCTCGTCCACCTCATGTTGTGTTCATACTTCAGAGTCCTCTCCTGACCTACGATTAAAGCACTATCACCGACACATCCTTGCATCTTGTTTACAGGAACCAGTCAACTTGCAGGCGTTACATCTTTGATTACTTGCTTGCATAGTGATTTGTTAAGAATAACAGATGCTGTACTTTACGTTTTCTTTTTAGTAATATACTCATTTCTTGC

>Podel.10G150900.1.v2.1

TACTACCACACTTGACAGCCTCTTTTGCCCCCACTCCCTCTCTCAAAACATGAGGGAAAGAGAGAAGGGTCTTTCAAAGGTGAAAGAAAGGCACAAGTTTCTGCAAGGTAACCTGTATAAGGGTATGAACAAAGCCATCATGTGTTACACTACAAGCCAGGAAGGAAGCCTAGTAGATGGATTCTTTGCTGGCTTTCAAAAGGCGGTTTCTTCATGTTAAGTGGCCGGTGCTATCCTACCTGAGCTTTTTCTATTATCTCTTCGTTTTTTTTTTTTGTTTTTTTTTCTTGACCTTGTAAGACCTTTTCTTGACCTTGTAAGACCCCATTTTTTGTTCTTTGTTTCTATTTTTTGTATTATCATCAATTTTACATCAAAATGTCTTGATGTAGTGTTTTGACTATTTTTTCCCCGTTTCCGTCCAACTCATCTTCTCCTTCCTTGTCTATCCCTCCTGAGCTATGAGCTGTTTGAATTAGACTAAATAACATTTCCCTTCAAAATCACCCAATCAAGTGCAAATCAAAAACCTCTCGAAGTTCATGCAAGCGATGGAGCCTTACTTGCCATGTGGGGTTTACGTTTTCTTACATGTGAACTACTGGAGGAAGAAATCAACAAGTTTGAGCTAAGTGTTCATTGAGGAGCTCGGTAAGAGCTTTAAAGAGTACTTGAATCGCTTGTAAAAGTCTTTGTCTTTCTTTCTCTGTAAAAAATAAAGAAAGACTCGTCTTTCTTATTCTCTGCACGTTAAGGCCATCAATTTAGATTGTTGTTAGTGTATTCGTGTGCAGAAATATGCTGTTGCAGACATCAGTTTCATTGTTTTATATGTTGTTGTCTCAGAAGTGAGTAAGTAAATATTCTCTCAGATCTGAGTGTGTAAGAGTTCTCCTCGTGACTGCTCTTATGTACAATACAAAATATTCATGTATCCACTGTGAAATTTGGATGT

>Podel.11G047000.2.v2.1

ACTGACACTCAAAATAAATAAAAAAATAAAAACGCTATTCAATCAACATTCATGTTTTTTCCATTTCTTTTATATATATATAAACCATACCCTGACCAGAACCTCTTGAGGCCAATAGCTTATCTCCCTCTACAAGTTCTCACTGCCAAAGCCTCTCTGTAGTGCATTGAAATGGAAGTATCTACTTCCAACATATTGAAATGTGATGATGAGGAGAAGGCATTGGAGGTCTTGCTTGAGGCATTTGGTTCTAAATTTTCTCTTGAACATATAGCATCTGCTTATTGCAAGGCAGGCCGAAATGCAGATTTGACTGTTCAAATTCTTCAAGATATGGAGGGAGGTGCCTCTACCTCTTCAAGTCATTCATCCAATGGAGAGGCTATGCTGAGTGAAGGATCTTCTGAGTCATCCAACGGTTATATTTTGAAGAAATGTGATGCAAATGGAAAGTTCAGAAATGTAAAGCAAAAATGGCGTCCAGTTTCAGGAGGCACTGTTTCAAGTGTTCTCGGAAAAAGTTACATTAGATCCATGCCAGTGGGCAATGGCTCTTGTGCCGCAACCAAACCATTGAAATTGGATGCGCAGGAGTTCCCAATGTCTGAACTTTGGGGAGAAGAACCCAAACAAACCCAATCAAAGCATGATCATATGCACAAGGATATGGAAGATTTTTATTCAAAATGCTAGGAGATGGCTTCCAGCTGGATAGGGAAATGATTCGACAAGCTCTGGATACATGTGGGTATGATATGCAGAAGAGCATGGAGAAACTACTTAATTTGTCAGCAGTGATTTTGGACAAAAGGAACAATTATGTTGGTAGATCAACTGGAAAGTTCACAGATGCACGGTCAAATAGTGGAGGACCTTCATGTCAAAAAAATTTACAGTTTATGAGTTCCTATGGAGGTGCAAATAGAATTTCAAATGCAAATGGGGGAGGATCACCTGGCCAGGGGAAAGAGAGAAACAACCTCCAGAAGGAAATTTTGGCCTCACTTTTTAATGGTGCTGAGAGATCTGGCGAGTTATCTGGAAGAATAACAAAGGCTGAGAGGAGGTCAATAGTATATGGAGAGCCAGTGGTTGAACCTCCTACTGACTTTACCTTAGAGAACAGGACTGATTTTATGGACTCTCTGCAAGATTATGACAATGTGTTATCAGTTGAAGATGTAGATGAAAATGATAGTTACCATCTTCTTCGTAAAGCTTGGAAGGAGTATCGGACCACAATGAATGAATTTTACAAAGCTGCTGGTGATGCGTTTGCCAAGGGAGATGATGAGCGAGCAAACAAACTAATGGACGAGGGAAATTTTTTTCGTGACAAGGCTTATGAGGTAGATGAGGAATCTACTCAGAAGATTTTTGGAACCAAAAATGTTGAGACTCAAGACCAGATGTTGCTTGATCTGCATGAACATGGTGCAAAAGATGCAATACGCTCCTTGAAGAGTAATTTTCTCTTACTCTCAGGCATCCCATCATTCAAGGACCTCAAAGTCATCATTGAGACAAATGAGGTGGATGCTACGAAAGGGGCTCGTAGAAGATTGATTATGAAGCTATTAGAGAAGGAATCGATAAACTGGACTGAAGGAGCGGATGTTGGAACAATACTAATTCAACTGGATAATATCAACCCTAAGCGCTTGAGTTTCGCCAAAAAATAGCATACAACATGGAATCAAGTGGGTTTCTTGAGAGGCAGGCAAGCAGC

>Podel.17G101000.1.v2.1

ATGGGTCCAGACTTTTGTCACTGGCCTGTGAACGTTGGACACCAAGATTTGTCCTTTCCTCTCTGTATAAAGAAGGATAAAGTGGAAACAGAGGCAGGCAACTTCCTCCGCCGTGTTGGAAAGGAAAGGGAGACTGCTTTACTGGAAGCTTCCGGTATGGGAGAGGCGGGAATGACTCTGAAAGAACTTAGAAGAGTCTTTCCGAGTCCTCCCATACCGCAGATTTCCAGTAGTTCCTCCCTTTACTTTTCTTCACTATTCGAGCAGCTCTGGATCACCAAGATCTATCATCCTGTGAGGTAGATCTTGTCAAGTCTGATGCTTTGCTCATATCGTACATGCCCAAGTCTCTTAGCTAAGGGATACGAGTAGTCGATTCTCTGCAGAAGTTCCCCAATTTGTTCTTGGCAACAGTAGCATGGAGGAATTGCTCGCTTCCTCATGGGAGAATCCCTTTGCTAGAGTTTTAGCTAAAATAACTAGAAGAAAGCTATTTTAGCCTGCTCGTGCTCTCTGAAACGGGGAGTGAGTTTGCAGAGAGTCTTGTATACTGAAAATTAATGATTTTAATTGCCTTTCTTCTTTCAATTCTTTGAAAAATTTTAAAAAAATTAATTTGATGCTGTGTTCTTCACATCTAATTCAAACATCAATCGTTTCAAGAACAAAAACAAGTAGATATAATATTAGTGTCTGAAGACAAGGAAGATTTGAGAAACTTCAGGTGAAGATTCACTAAGATGGTTTTCTAACAAATCAAGGCAGTTAATAAGAAATTTCGAACACCGAAATGCCTTCATGGCAAACTAGTAAAAGTCATCCAAGAGATTGTTTTCAAAATGTTTTGTTTTAATGCTGGTATATTAAAATCATTAAAAATATAAAAAAATATTAAACTAAAACTAAAAAAAAATTAAAAATTTTAAAAAAGCTTGGTTCCACAAGTTCATCATCTGATGATTCATGTTTATATCAAAGCCTTTAAGCATCACAATAGTAAAACATGAGTTCATGAACATGAATCATAAGTTATTCTTAAATTATAAATC

>Podel.T205100.1.v2.1

AAAATAATTTTGAAAAAAAAAATAAATGAAAAAAAAGAAAAAAAAAGGGAATGTTGGAAACAAAAATGAAAAAAAAAAATTGTATGTAGTTTATTTTTTATTCTATCAAACTTGCATGCCTTTTAATATTCTCATGAATTGAGACTATCTTTGTGGTGTTTTGCTTTTAAAAGTTGATGTGGTTTTCTCCATAAAGACTCAAAAGCCTTCTCTGTAGTCTTTTTCTCGTCTAATTGAAGTTGGTTCTGTTAGATTGTCACAAGAAGAAGGCGCCCCCCCCGGATGCCAAAGCTACGGGGGCGTGCTCCTAGTACTCAATTTCCTTGGCGCTTCTTGCGCCGGGGTTCGTTTGCAAGCCACGCGGGGCGCAGAGGCATCCCTCCATGGCCTGCGAGGATGCGAGGGGCGGGTGCCCCCCGAGCTCAGCTTGTCATGCCACGGGTTTGCGGGTCGTTCTTTTAGGCAAGTTTCGACAATGATCCTTCCGCAGGTTCATCAACGGGAACCTTGTTACGACTTCTCCTTCCTCTAAATGATAAGGTTCAGTGGACTTCTCGTGACTTTGCCGGCGGCGAACCGCCCACGTCGCCGCGATCCGAACACTTCACCGGACCATTCAATCGGTAGGAGCGACGGGCGGTGTGTACAAAGGGCAGGGACGTGGTCAATGCGAGCTGATGACTCGTGCTTACTAGGAATTCCTCGTTGAAGACCAACAATTGCAATGATCTATCCCCATCACGATGAAGTTTCAAAGATTACCCGGGCCTGTCGGCCAAGGTTATAGACTCTGTGAATACATCAGTGTAGCGCGCGTGTGGCCCATAACATCTAAGGGCATCATATACCTGTTATTGCCTCAAACTTCCTTGGCCTGGAAGGCCATAGTCCCTCTAAGAAGTTGGCCGCGGAGGGCCATCTTCGCATAGCTAGTTAGCAGGCTAAGGTCTCGTTCGTTAACGGAATTAACCAGACAAATCCCTCCACCAACTAAGAATGACCATGCACCACCACCTATAGAATCAAGAAAGAGCTCTCAGTTAGCCAGTCGAGATCCTAGACAGAGAAATTAGAGAGAGATGCTCTCCTCCCGTGAGAATTTCCCTTGTGCTTTGTGATCTCCTGCCACTGTTCACTTTCTTCGTTCCCTTGATCTGTTGTCTCTTGGCCTTCTCCTGGCTATGGCTAACAAAAAGAAGAAGGCTGGGTCCCGTAGATCTCAGTTAATGCAACACAAGTCGCTGGCTATTTCCCAGCGTAATGATTCTGCTGTCCTAGCTTTAGCAGCCCCGCCTCTTACTGTTCATGCTACAGTAACCTCAAATTCAAATTCCAAAAAGCGCCCGCCTGTAGCTTCTGCCGCTGTCTGCTCTCGACCTATCCACCATGTACTCTCCTCCCCACAAGGTTCTGAGTCCCATCATCCTGGTGATCCATCTCCCAGTATCAATCAAGTGTTTGTGGAGGATTGGTCTAGTGATGAGGATCTCGAAGAAGAGGAGGTGGATTTTGACTCTTCTGTGGACTTTGACTATGTTGGGGCACCTTCATCCTCCTCCCCAATAGTCGTGGCTTCCCCTGCTAGTTGTCAGTCCACCCCTCCTCCTCCGTCTCTTGCTGAGGAGATTCTTTCTCCCCCTGTTGCCTCCCCTGCAGGTGGCCAGTCCACCCTGCCTCCTCAACCCCTTGCTGCGAAGATTCTTCCTACCCCGCTGGAGACGACGGTGCTGCCTTCTTCTCTGCCCTCTGGCTGTCGTGAGTCTCCTCCCTCCACCGTAGCAGGTAACCCTGTCTCTATTCCTGGTAGTAGCAAATGGAGTGACCTTTTTCTCTCCAATAGAAATACTGCTTCTTCTACCAAACTACAACACTTCTCTCTTAATCACCTATCTAGAACTTGTGCCATCTCACCTGAGGATATTGCACCTGAATTTGATGTTTGGCAATATTGTGCTGTGGGATATGTATCTGGGAAAAGGCCAGGTTATGGAGCCCTAAACAGTATGATTTCCACTGTTTGGAAATGTGAAGCCTCCCTCTCCATCCATGACTCTGGGTGGCTGGTTTATCAATTTAAAACAGAGGAAGCCAAGAATTCGGTTCTTAGTGGCGGTCCTTACTTAATATATGGACGACCTCTCATTTTGCGGCCTATGACGAAATTCTTTGATTTTTCCTGTGAGGAAATGTCGAGGGTTCCTGTTTGGGTTAAATTCCCCAACCTTCCCCTGTGCTGTTGGTCACCTATTTGTCTATCAAAGATAGCCAGTGTGCTTGGGAAGCCTATTCAATGTGATCAGCCTACCTCCACTCTTTCACGTCTGTCTTATGCCCGGGTCCTGGTTGAAATTGATCTTCTTGAGGAAATCCAACACTCAGTTGAGATCTCTCTGCCTGAAGGTCCTGCCTTACACCAATCGGTTGTATATGAAACTCTGCCCAAGTATTGCACTTTCTGTCATGTTCTTGGTCATGCCCGGCTCCTTTGCCCTAAGGCTGCTGCTTCTAAAGCTAAGCCCTGCCATCAACCCCTGGCTCTGTCTCTTCAAGCTGCTAAAAGGGATGTCCTTAGTAGATTGGGTCCTCAACCTCCTCTCCATCCTCCTCTGCCACAGGTGCAAGTCCAACATGCAGATGCCATACCTGTGGTTTCCAAAGGGGATGTTGTGTCCGAAGTTGCTCTTGAACCTACTAATGGTTGGGTCACAGTGGAAGCAAGACGCAAACCAAGAAAACAGGTTAAGGGAAAGGCGGTAGTGGTATCTGAGCCAGTGTTGGAGGCTATCTCCCCTATCCCTTCCTCTTCCCCTGTTTGCACAGGACCAGTCCAGATTCCACTGGTGACAACCCCTTGTGCTGCAAGTGTTCAGGCTTCTCCCCCTCCTTGTCCTGACTCCTTAGCTCAGCCTCCTAGTGCTGACAAAGAACAGATTCAACCAAGTACTCCAGCCACCACTGCTGGTGATGATCATATGCCTGGCCCGCCTATCCCTTCTCCTATAGTGGGGAAGTCGGTCCTGAGTCGAGTTCTCACTCGAAACCAGAAGAAGCGAGGTGGTAGGGATAGGATTTCCCCTCCCATTACTTGTTGATGCTTACTTTCAGCTCGTCTTTAAGGGGTCCTCCTATCCCCCTGGTGCAGCATGCTTTTTTGGTTCTGTACCGGGTGCTGGGTGGTGCTGTTTTTGTTTCTTGCTGCAGGAGTTGTTTGCTGCTAGTTGATGTCTCTTAGTCAACTGTGTGGAAATGCTATCTGGGCATCGTCTCTATTGTTGTTTTGTCTGCAATGGTGCTTGCTGTCCTTTGTGTGCTTTTGTTCATGCGGGGTGCTGGGTGGTGCTGTTTTTGTTTCTTGCTGCAGGGGCCTTTTTCGCCTTGTGAATTGCCACTTTGCTATTGCATGGCTCATGCATATTGATTTCACGTGACAATTACATTTTGCTGATGGGGCCCCCTTCGTTTGGACGATTTATTGGCTCTTGCTGCTGTTGGTTGGCTGCTCTTCTGTGCCGCTTCCTATGTAATTGTATTGTAATCTCTTTTGTTCCCCTCGGTTATAATGGCTGGCCGAAGGTTTTGCTTGTCTACCCTGCATGAGGGTTGTTCTTGCTATTGTATATGGGTTTCTTTTCCTTTCTTGGTCTGGCTTGTTATGGGGAGTCTGTTCCCCTACCCTTGCCTTTTCTGTTTTCTTTGCTCCGATCAAGGGAGTTTGTTCCCTCTGAT
