# Supplementary material for: Identification of Populus Small RNAs Responsive to Mutualistic Interactions With Mycorrhizal Fungi, Laccaria bicolor and Rhizophagus irregularis
Source: Front Microbiol. 2019 Mar 18;10:515. doi: 10.3389/fmicb.2019.00515 (PMC6431645; doi:10.3389/fmicb.2019.00515)
Supplement: Supplementary file 2 [file Table_2.DOCX]

Sequences of differentially expressed *Populus trichocarpa* transcripts in fungal treatments relative to control.

>Potri.CUFF.100.1

GAAGGGGGAAAAAGTAAAAATA

>Potri.CUFF.1076.1

AGTTCTACCTCTAAGATACTCAA

>Potri.CUFF.1118.1

CAATTAAATTTAAATCAAGAATATCATT

>Potri.CUFF.1120.1

GTGAGACTAATCCCGTTCGGAGTTCGTGGCTGTCCTTCTCAATAAGTACGCTTCCTGGGGATCGAACTTGTATTCTCACCCTTACAGGAATATAGTAGTGCCTTAACCGTTAGACCAATACTTCATTGGTCTCAACCGATTAT

>Potri.CUFF.1124.1

AGGTGAGCGAGTAAAATCCCGGCTGTCCCAAACTCTTATAAGAGATGCACGTCCTGACTTGAACTCGAGACCTGCTGTGCAAATCTCAAATCCTTTGCCATCATGCCACGCC

>Potri.CUFF.1177.1

TTTATGGAGAATAGTGTTTAAGACGTCAAGTGTAACTAAGCTTATATAAACTGGGTTCTTCCCCGAGGTTAGACTAAAGGGAGGTTGGTCACTCTCCTGGATATGTCAAGAGAGGATGATAATTATCTTGGACTTGGTTGATTGTCAATCCAAACATATTGGGTCTGACATG

>Potri.CUFF.1300.1

AACCAAGCCCAACAACAAAGA

>Potri.CUFF.1343.1

TTTGTTAAATCATGTTAGTTCTCAACATTATAATCATTGATTCTTTGAATTAAATTTTGAAACTCTTCTCAAGACTTATTCTACATTGA

>Potri.CUFF.1416.1

CTTGGACTAATTTAGATTTTAAATCAGTCGGTTTCAAGTTAATCCTCTGATCGAAGTTTT

>Potri.CUFF.1436.1

AATGAGATAAGTGTCCCTCTGACTATTTAGCT

>Potri.CUFF.172.1

TCAGATTCGAGTCCTGTGATTGTTAATATGATAACCATTGAAGATTTATATGGTCGTTAACTTCAGAACTCATAAGATTAGTTAAGGTGCGTGTAAGTTGACCCGGACATC

>Potri.CUFF.180.1

AAGGCATCATACAAAGTCAAGAACCCAAAACAGTGTTGTCTAAATCCAACTCCTCCGGTTCATCATCTCCAGCATATTCCCTTGGAAATGATAACGAAGATGACTGCTTTGGCATCCTGCCACCCA

>Potri.CUFF.184.1

TTTGACAGTGGAGTAGTTCTTCTGACAATCATTCCAATGAGCAGATGTATATTGGAGGATTTGTTCTTTGTTGTTTCGGACTTGTTTAAGAATCCCTCGGTAACCCAAGTCTGAGGTATCAGTCGCGACTACCTTAGGAGCTAGTGGAGAAGCAGAATAGTTTGAACCTGCTGTTTGATATGTCGGACTAT

>Potri.CUFF.225.1

CAGCTCTATATTGACACAACTTTT

>Potri.CUFF.2346.1

GCTTCAAAAACAAATGTAGATACTAGGATGGCT

>Potri.CUFF.2450.1

TTTTTTTTTAGCTATTGAACT

>Potri.CUFF.2719.1

AGCTCATGAGACTCAGATGAATGACATCCCTGCTCACCACACTTGTTCCATCAAACTTTGGAATCAAAGC

>Potri.CUFF.2730.1

TTAGCCCCCGGCGAGGATCGAACTCGCGACCTTTCGCTTACGAAGCGAACGCACTACCACTATGCTACGGAGGC

>Potri.CUFF.2778.1

TGTTCTGAACCCCTCACCTCGACCGCTACACAGAAACAATTGACAAGGTGCCAACCTCTAGAGTGGCGGTTGAGGGAGAGAGTTCAGAA

>Potri.CUFF.2779.1

AGCGGTTGAGGGAGAGAGTTCAGAA

>Potri.CUFF.2945.1

ACGTCGACAGCAGGGTTCGAACCTGCGCGGGCGAAGCCCAATAGATTTCAAGTCTATCTCCTTAACCACTCGGACATATCGACGCTCAT

>Potri.CUFF.3156.1

AATCTTCCTCTTACTTCTGTCTCGGTCAATTTGCATCCCTAGAATCTTATTTGTTGGTCCCAAGTCCTTCATATCAAACTCGCTAGCCAACTATGCCTTCAATTCTTGGACTCGATCTTTGTTGGGGCCTA

>Potri.CUFF.3241.1

AGATACTGGCTCGGTTCAACCTCATCGTAGTCTTGATGAATGATTCAAATCTATGGTTGGATTGAGCCGCGCCAATATCGCATG

>Potri.CUFF.3413.1

GTCTCTTTTGAGTTCGAGTCAGGACATACTTTCTTGTAAGAGCCTGAAATAACTAGGATTCTACTCGTTCATCTG

>Potri.CUFF.3562.1

CATCAAACTTTCGAATTTAGGC

>Potri.CUFF.387.1

TTCTGATCAAATGACCCGAGTC

>Potri.CUFF.3925.1

CTCACGACCCTCAAACAGTCATAA

>Potri.CUFF.699.1

TGTCTTGTTCCAGTCATCATTA

>Potri.CUFF.73.1

TTACATCACCGATTGAATGACTGACAGA

>Potri.CUFF.827.1

TGAAAATCCAGGTGAAGAAAG

>Potri.007G077200.1.v3.1

TCTTTCAATTTTCTATGTAATTGTTTCTTGGTTGTTTTATGGATATACTAATGAATGGATGATGATGTTAGAGAGGTCAATGATGATAGATCCAAAGGCTTGTTTCTCAAGAACATTCGCAGTGGCCGCCTAAGAGCTTTCGCCTTTCGCCCAAGGCTTGAGAGCTTCTGCTGCATCTTTCCTTCCTTGGATAGTTTGATCCAGTTTTGCGATTATTTTGCAAATTTACACAATGACCCAGCTGCTTGA

>Potri.013G036500.1.v3.1

ATCCTTGACGAGGTAGATCATTAGTGGCTACCAGTACTCGAGAAATGACACCTACATGGACTGATTAACTGATTCCATTCCATGTCTGTTACAAAAATGGCGGGAATATGCTCTCTGAATCCAACAACCTTAATGACTTTTAAGACAATTCATGGAATTAGATGCTGCTCAGGAGCAACAGATAATGAAAAGAAGAGCCAAACTAGAACTAAAACCCCACAGATTTTGAAATTGGCTGTAAGTGGGGTCACAGAGCTTTTAAGGGTTTTCTCCTTCTCGGGCAAAGAGAGATTAGAGAAAGTGAACAATAAAGATAGAGATGAGATATCTGTTTCTGGTATTGATGATGTTATAATGATCCTCAAGTCTGATTATGAGAATGCTTATTTCGTCACAGGTACTAAGTTGTATTCACGCAATTTGAAATTGCTGGTTCCTTTCTTCGATTGTCCATCAATTGGATTACAAGACATTGAGAAGGGTGTCAATTCCGAAACATATTTTGTGCTGGCAAGATGGAAACTAAGAACATACCTGAAATTCCCATGGAGGCCTCTCATTTCAATCGACGGAAGCACAGTCTATGAATTAGATAACAAGCTTAAAATTGTTAGGCATGCTGAGAGCTGGAATGTTTCTGCACTTGAAGCTATTGGTCAGATATTTACCCCAAGCTTTGATAGACCTGGTGAATGAATTTGAGAAAACGTCTGGAAATCTAAAGCTGATGTAATCAGATCTCTGCAATTTGATTAAAAAGATCTTGTGGTTTTAAAGACAATGTAGACTTTCGTATGGATTAAGTTAGCAACCATCATCCTGTCTGCTCAAAGCAACTCTATAGTTGGATGGAAAAACAAAATGTTCCGTTGCCGGGACTTGAACCCGGGTCTCTCGGGTGAGAGCCGAGTATCCTAACCAACTAGACTACAACGGATTCTGCTTATTTACCTGTGGCAACTAATATACAACTCTTATTAAAATTGCATGGAATAAAAAACACAAAAATCACCGCATGTTCTCTTGCTGATAGAGCATGAGATATGACAGGCGGCTGGAGCACAAGGCAAAGAGAGAGGCTGAGGCTTGTTGAGAGCGAGATATATAGCGCTTCTATTTCCATGGATTGGAGTAGTGCTGCACCCCAGGCTTGTTAAAAACAGAATATTTGCTGCTTGTATGTTTTCTTTCGAGGTTGGCTAGATTATTATAAGGGACAAAAGGTATACTATTGCAATGATGGTAGGAGCATGGCCACCAAGCACAAGAAGAATATTTGCGTTGTGCGTAAAAGTAGTAATGAGAGCTACTCATGTTTTAGCGCAGAAAGTCTTTCATCACCTTGATAACAAAAAGGAACCAACCTCCTTGAAATTTTCAGTTCTTGTTTGGAGTGAGTGTCTGGGAATCCTAGGTGCTGTGGTGGTGGAGATGGCGGTGGTGCATGCGCGCATGGTTTCAATATTTCCTTGCTATTCAGTTGCTGGTTCATGTGGGGCTCGTGAATAGCAGGGTTTTTTGAACTTGAGGTCTTGGTGCCCTGTTTCTTCCTACTTAACGACGGGATTGAAAGAAGAAAGTTATGATTTCCGGTGAAAAGGGAGTGATGATTGCTTCCCTCTAGTCTCTGTCGTCCTTAAAAAAATACTCCAGAAAGATTTTTAATCAGTTAGTTATGCCAGGGTCCATCAACTGGAAGCCTTTAGGCGAAGGTCCTTGTGTAACCTTTTTG

>Potri.CUFF.1088.1

TCCATTCCTTCTTTTCCCACGT

>Potri.CUFF.1277.1

TAAGATGATGAACTATGCTTGAACGGATGAAG

>Potri.CUFF.1386.1

TTGCGGGTCCCAACAAATGCGCTCT

>Potri.CUFF.160.1

TCCATCTAAACCTAAATATTA

>Potri.CUFF.1625.1

TGAAAATGTTGGTGATATTTA

>Potri.CUFF.167.1

CACCCTTTTCAAAGTGCTTTTCATCTA

>Potri.CUFF.1994.1

TGAAATGTGATATTTGGTGTGTGTGGAAAAATAAGTGTTGGGAAGAAATGAAAAGGGTTAGTTGGTGGTGAAATACGGGTCTAATTTAAAA

>Potri.CUFF.2551.1

AAAACACCAAAAGTTGCAAATAA

>Potri.CUFF.262.1

TAGAGGGTCTCCATGGCCCATAAA

>Potri.CUFF.2745.1

GTATCTACCATCACAATTTCTGTGCAAGGAACAACAGTTGCATTCCTTGTAT

>Potri.CUFF.2746.1

TGAGGCGGAATCAAACTCTCCGTAA

>Potri.CUFF.2885.1

TCAAGAGAATTATGGTAGACTAATTGGTTATTTATA

>Potri.CUFF.3145.1

GACTTAGACCAAAGAGTGGTCTTTCAGAATTGGTC

>Potri.CUFF.347.1

AGGGGAAAAAACTAATCGAACCATCTTGTAGTTGGTTTCCTCTAAAGTGTTCCTCATGATAACTAGAGCTCAATGTGAGTTCTATCGGGTAAAGACAATGATTAAAGGCATCGAGGGCATAACTCCCTTAACCTATTCTCAAACTTTAAATATGTAGGGAAGACGTGGCTACTTCATTGAGTCGTGTCACATAATCAAGGGCTCTAAGTGGGCCATTTTTGGTAAG

>Potri.CUFF.3734.1

CGGTCAGGCCGATCCGGGTTGAAAAAA

>Potri.CUFF.3792.1

TAATCTATAGGTATATGATCCC

>Potri.CUFF.540.1

ACTTGATGTTTTTGATTATTT

>Potri.CUFF.68.1

GGGATTCGAACCTGCACCCTC

>Potri.CUFF.690.1

ATCGCAATGATAAGGGCTATGATGTAAAATTAAGTCGGCCATAATGATCAGGGT

>Potri.001G111100.1.v3.1

TCATCACCATTAACCAAAAGCCCCAAAAAGAAAATTGAAAAATAATTCAAAAGCAAGAAAAACCCTATTTTGGTTCCCTTTCTCTCCTTGCAGCTTTTCAAGAAAAGAAGAAATCATGAAAGGAGCGAAACGATTTGCCGCTTCAGACCCAGTCCCTGACTCGAATGACACGGCATTAAGGAATAAGAGAATAATGGAAGGATCGCTATTTGATATTCATAGACCTGAACAATCTCAGCAACAATCGACTCCATTGCCACCGTTGGATGCAAAACGGGCTGCTTCATCACAGCAGTATGTGAGAGCTCTCAATAACCAATTTGCCAGTTGGGTTCAAACGCAACTGAAGAACCATCCTGATGAACTCTGGGAAGATGGGATTCGAGATTACCTTGCTCATGCTTCAAACATTATGGAGAAGTTTAGTGATGTTGTCAACTGGCTTAAAGCAAATGCTGTAAAAGGAGGGCCTGTTGCTGATTCTCTTCGAGCTGAAAAGAAATTAGTGCCTGAAATAAAGAGTAATGAGAGCAAATTACTTCAAGAAAAAACCGGGTTTGCTTTACCGAGTACCAGTACAAGCTTTACAAGTTCCTGGAGCTCTGGTGTCTTTTCTGCCAACCAAAGTTCTGGAGGAGTATCATCTAGTAGCCAAAGCTCTAGTTTATTCTCCAATGGTCAAAGTTCTGGTTCATTATTATCGAACAATCCAAGTTCTGTTTTGTCCTCAAACAACCAAAGCTCTGGATTTTTCTCGAACATTCAAAGCCCTGGCCTCTTGTCCAACAACCAAAGCTCTGGATTTTTCTCGAACATTCAAAGCCCTGGCCTCTTGTCCAACAATCAAAGCTCTGGAGCATTCTCCAACAGCCAGAGTTTAGGAGCACTCTCCAACAGTCAAACACCTTTCTCCTTTAATCAAAGCTCTGGAACATTCTCCAACAGCCAAAGTTTAGGAGCACTCCCCAACACTCAAACATCTTTCTTGTTTGGAGGCCAAAGCTCCATCCCCGCAAACCATAACACTGCAGATGATGCGGATGATGAAAATGAATTACAGCAACCTGGCAGTCCATCTGTGAAGAAGTCTGAAGAGAAGGGTATTGTTACAGTCCATGAAGTCAAGTGCAAGCTCTATGTGAAGTCAAGTGATCCGGCAGATAAGGACACATGGAAAGATAAGGGCCCAGGGCAGCTTTCCATCAAATGCAGAGAGGGGATTGGCAAGTCCACGAAAGAATCTAAACCAACCATTGTTGTTCGAAATGATGTGGGGAAAGTGTTGCTTAATGCTTTGCTCTATCCAGGAATCAAGACAAATCCACAGAAGAATTCCCTTGTTGCAATATTTCACACTGCAGGTGATGATAGCGGCAATAATGATAGTGTTGTGGCACGTACTTTCTTAATTAGGACAAAAACAGAGGAGGATCGGAATAAGCTAGCGACAGCAATCCAAGAATACGCTCCCACTTCATGAAAGTCATTATAGGAGCACGATTCTTAAAATTTTGCAGCAATTGGCTTGTTAGGAACTGAGCCAGCTTGTCTTGGGGCTCCAAAATAACAGGGGTCAATTTTTAGAACTCGGACCCCATGAAATCAAATGTATTTCTATTACTTGTATTATAATAGGAGGAAAAGGAATGCTATGTTTTGTTTCAATCTTAAACCGTTATTATCCTTTTCTAGTTAACTTGTTTATTTGATGAATGAGCACGTCATCATGTGACTGGCATCATTCACGGTGTCCAAACGCCTTAATTTATGCAATGCTATCAAAATTTTCTTCTCTCTCTAAAGTGTAATTGAAGGCAAATAGAATCAAGAGATTGCAGGTTCTCTTGCACAAGTTGCCCACAGCACAGCC

>Potri.003G048200.1.v3.1

CTGGTCTTTAGCTTATCGTTTCTTTATCATTTTCATTTCCTCTGAAGAAGAGCGGGGATGAAACACAGGTTGGAGAAAAATGGAGAAGGATGCCGCATCGGAGATAAGAGAATACTGCCGAGAGAGGGTAGGTGGTGCTGTTGGCAGTGGTGGTTCTTCTCCTTTTTCTAACACAAACTGATAGACGAAAGCCGAAAGAGGAGGCACCATACAAGAAAAAAAGGGGGGCAAGGAAGCCATTACGAAAAAGAGAAATCTACAGAGCGATGGCCTTGCTCTCTCGTCACTGATGATGAGGATGGTTTTTAAGGTTGGGGAACATCAGGAGGATTTTGTTGTGGGAGATAAGAACTGAAAGATGAGGTTCGAATTTATACATGGATGATGTTGCTCTTCGCGGGTAATTGATATGGTACAAAGGAAACATGTATATGATATTAAGCAATTGATTTTAACCAGAGATGGTGGAAGTTGTTCTCAACATGTATTCATGACATGACTCATCAGCATGAGATGTTTGATCAAGATCATGACCAAGATTGGAATAACAGTAGGAAGCTGCAGCCACAGGCACCAAGATCACCTTATGCCTGGCTCAAATCAACAACCGATGATTTGGATATTAAGGATAAGTATCTTGGGTTGATTGGAAAGAGAGGAAAGAACATGAAACATCTTTGTCCAGAGGATTTTGGGTATGATCCTCGAAACTATTCTCATAACTTTGAGGATGATTTTCGCAGAGAAGATGAATTGTGCTCAATCATAATTGTAGGGGAAAATTTCCTACCACACGAGAGATTGGTGGTGCTGCTGCAGATAAGAAGATAAGGATTGTTACCAAGTCAAGAGGTGACCACCTACTCAACAATTAGTCATCAAATCAATAGTTCCGAATTTTGGCCTATAAAAGGAGGCATTTGCCATGCATTTAGGCATCTTGGTTTTCAGATCAAGATCATATTCTTGTTTCTTTCTTTGTATTTTTATAATGTTTAAGTTTTGCTTTAATTAATCTCTTGTTTATGCTTTTCATCATAACTATGTTCTTATCTAGTTTATTTATGTTTCTCTTATTCACAATGTTTAGCTGAGTTTATTATGTCAAGGTGAAAATGTTTCACTAATGGTGTCAGAATAGGTATAATATAAACTCGACATGGACTTCAATGTTTATATTCAAGAAAGTTTGTTATTA

>Potri.005G145000.1.v3.1

TCATCACCTAATAAATAAAACAGATACACTTCTCGTATCTCAACAGTTACCGTGCAACTTGCTTCGGTACAGTTAGTTTCCTTTTAATGGAATTGTAAGGTTGGTATATCACATTCAGAGCAGGTGTCTTTCGCATCACAGCTTCATCACCGCCAAATCGAAATTAGACCCCTCATGAAGAATTGGCTATCTGACCACAACCGTTAGATTGGAGATAGAATCCTGCCCAGCTGCATCTTTCAGCACCACCAAAGAAGAACCCAATAATCTTTTATCATAAAATATCCATACAGCACATATGGTCCTTATTATATCACCCGTCCCTCCTTTCATTCATGGTTGTATAAAACGACAAACGACAAGCCTACTCTATCTTCACGCCATTAAGAAACAAACCGATCCGACAAGAGTCCAAAGAAGTAGCTAGTTCCAAGGAAAAGAGACTAGATGGGTGTTGTAGAAGAAGCTCATAACGCGAAGATCTTGGGTTCAGGACAGCAAGTGATAGTTTTGGCTCATGGGTTTGGGACAGATCAGTCTGTCTGGAAGCACTTAGTTCCCCACCTTGTTGATGAGTACACTGTTATTTTGTATGATAACATGGGAGCTGGTACTACAAATCCAGATTACTTTGATTTCAGTAGGTACTCTACCCTCGAAGGTTTTGCTTATGATTTACTTGCCATTTTAGAGGAGCTGCATGTTGAGTCTTGTATTTTTGTTGGTCACTCCGTTTCTGGCATGGTTGGTGTTATTGCCTCCATTAGTCGCCCTGATCTCTTCTCTAAAATTGTCATGCTTTCTGCTTCTCCAAGGTACTTGAATGATGTTGATTATTATGGAGGATTCGAGCAGGAAGATTTAGACCAATTATTTGAAGCAATGCAAAACAATTACAAAGCATGGTGTTCTGGTTTTGCCCCACTAGCCGTGGGTGGAGACATGGATTCAATAGCCGTGCAAGAATTCAGCCGCACACTCTTCAATATGAGACCAGACATAGCCCTTAGCGTGGCACAGACCATCTTCCACAGTGACATGAGGGCAATCCTACATATGGTCACAGTCCCCTGTCACATCCTGCAGAGCATGAAGGACTTGGCTGTGCCTGTGGTTGCCTCTGAACATTTGCACCAAAATCTTGGTGGTGAGTCCATTGTTGAAGTCATGTCATCTGATGGTCACCTGCCTCAGTTGAGCTCTCCCGACATTGTGATCCCTGTGCTTCTTAAGCACATTCGTTTCAATATAGCTGCGTAATTTGTACTGTCAAACTCTTGTTTCCCTGCAGATTGTGTTTGCTTCTTCTTTTTTTTTGTTTTGATAGTTAATCCGGAAGTGAGTGAATGGTTGTACCATGACTGTAACGTCCATTGTGGTTTGGCTGTGTCAAGTTGTGATTACTCATCATGTAATAAGATTCAAGAGAACATGGAGTTCGAATAGGCCTTTTTTCAAACAAAGAAAGCGAAAGAAGTTCGGTTCTTGCTTGCAAACTACATAATAAACACGCTTTCA

>Potri.011G051300.1.v3.1

TTTATATATATATATATGTAAACCATACCCTGACCAGAACCTCTTGAGGCCAATAGCTTATCTCCCCCTACAAGTTCTCACCGCCAAAGCCTCTCTGTAGTGCATTGAAATGGAAGTATCTACTTCCAACATATTGAAATGTGATGATGAGGAGAAGGCATTGGAGGTCTTGCTTGAGGCATTTGGTTCTAAATTTTCTCTTGAACAAATAGCATCTGCTTATTGCAAGGCAGGCCGAAATGCAGATTTGACTGTTCAAATTCTTCAAGATATGGAGGGAGGTGCCTCTACCTCTTCAAGTCATTCATCCAATGGAGAGGCTATGCTGAGTGAAGGATCTTCTGAGTCATCCAATGGTTATATTTTGAAGAAATGTGATGCAAATGGAAAGTTCAGAAATGTAAAGCAAAAATGGCGTCCAGTTTCAGGAGGCACCGTTTCAAGTGTTCTCGGAAAAAGTTACATTAGATCCATGCCAGTGGGCAATGGCTCTTGTGCCGCAACCAAACCATTGAAATTGGACGCGCAGGAGTTCCCAATGTCTGAACTTTGGGGAGAAGAACCCAAACAAACCCAATCAAAGCATGATCGTATGCATGAGGATATGGAAGATTTTTTATTCAAAATGCTAGGAGATGGCTTCCAGCTGGATAGGGAAATGATTCGACAAGCTCTAGATACATGTGGGTATGATATGCAAAAGAGCATGGAGAAACTACTTAATTTGTCAGCAGTGATTTTGGACAAAAGGAACAATTATGTTGGTAGATCCACTGGAAAGTTCACAGATGCACGGTCAAATAGTGGAGGACCTTCATGTCAAAAAAATTTACAGTTTATGAGTTCCTATGGAGGCAGTGCAAATAGAATTTCAAATGCAAATGGGGGAGGATCACCTGGCCAGGGGAAAGAGAAAAACAACCTCCAGAAGGAAATTTTGGCCTCACTTTTTAATGGTGCTGAGAGATCTGGGGAATTATCTGGAAGAATAACAAAGGCTGAGAGGAGGTCAATAGTATATGGAGAGCCAGTGGTTGAACCTCCTACTGACTTTACCTTAGAGAACAGGACTGATTTTATGGACTCTCTGCAAGATTATGACAATGTGTTATCAGTTGAAGATGTAGATGAAGATGATAGTTACCATCTTCTTCGTAAAGCTTGGAAGGAGTATCGGACCACAATGAATGAATTTTACAAAGCTGCTGGTGATGCGTTTGCCAAGGGAGATGATGAGCGAGCAAACAAACTAATGGACGAGGGAAATTTTTTTCGTGACAAGGCTTATGAGGTAGATGAGGAATCTACTCAGAAGATTTTTGGAATAAATGTTGAGACTCAAGACCAGATGTTGCTTGATCTGCATGAACATGGTGCAAAAGATGCAATACGCTCCTTGAAGAGTAATTTTCTCTTACTCTCAGGCATCCCATCATTCAAGGACCTCAAAGTCATCATTGAGACAAATGAGGTGGATGTCACGAAAGGGGCTCGTAGAAGATTGATTATGAAGCTATTAGAGAAGGAATCGATAAACTGGACTGAAGGAGCGGATGTTGGAACAATACTAATTCAATTGGATAATATCAACCCTAAGCGCTTGAGTTTCGCCAAAAAATAGCATACAACATGGAATCAAGTGGGTTTCTTGAGAGGCAGGCAAGCAGCCACCATTTTTTTTTTGAGAATCATTGTCATGATCATTGTCATGATCATTTGTTGACAAGTTACTATTTATCAAAATGTTGACCGTGTAAACACTATAAAGAAGCAACGAAGAATACTTTGATGTATTCTCAACTCGTCTTCTGTAATCCAGTAATATGTTTCAGCTTTCTTGA

>Potri.019G023600.1.v3.1

ATGCTTTCAAGTACAGTTGCAGGATCTGTTTTAGATCTCAACTCAAATAACTTATCCAAAGACGATAATATTGTCTTAAAACCTTCCCTTGTCTCTGCAGGATCTCTCACTGCTTGTCAGGAAAGAAACAGATTAATACTTAAAAATCAAGTAATTAAGTGCCGTACTCTCCTTTCATCCATAACCTTTGGGCACATCTGTCTCTCCCTCTTTCTCTCTCTCTCTCTCTCCACTTCCACCATGGCCTGCTCAGCCTCTGACCTAGCTCCTCTCCTCTCCACCACTGTAAACTCCACAGAAGCTGCCACCTACTTATGCTCCCAGTTCACCAGCATATCCAGCCAGCTTTCAGACACAAGCTATGCAATCAACAACACTTACCTTCTTTTCTCTGCCTACCTAGTCTTTGCTATGCAACTTGGCTTTGCCATGCTTTGTGCAGGCTCTGTGAGGGCCAAGAACACCATGAACATAATGCTTACTAATGTTCTTGATGCTGCTGCTGGTGGCCTTTCTTACTATCTCTTTGGCTACGCCTTTGCCTTCGGCTCTCCCGGCAATGGCTTCATTGGCCGCCATTTATTTGGTTTGAGTGACTTTCCTACTATTCAAGCTGATTATAGTTTCTTCCTCTATCAATGGGCTTTTGCTATAGCTGCTGCTGGCATCACTAGTGGCTCCATTGCTGAGAGAACCCAATTCGTTGCTTACCTCATATACTCCTCCTTCTTGACCGGCTTTGTTTACCCTGTTGTTTCACATTGGCTTTGGTCCGGTGATGGTTGGGCCAATCCCGCTAAAACCGATAACAAGCTCTTATTCGGTTCGGGTGCAATCGACTTTGCTGGTTCAGGTGTGGTTCACATGGTTGGAGGTATTGCGGGTTTGTGGGGCGCTCTCATTGAAGGCCCACGAATTGGCCGGTTCGACCAGAACGGTCGGTCCGTGGCCTTACGTGGTCATAGCGCTTCGTTAGTGGTGCTCGGTTCATTCTTGTTGTGGTTCGGGTGGTATGGGTTCAACCCCGGTTCGTTCTTGACAATCTTGAAGAGTTATGGTGGTAACAGAGTGTTTTATGGTCAATGGAGTGCTGTGGGAAGGACAGCTGTCACTACAACATTGGCTGGGAGCACAGCTGCCCTTACTACCCTGTTTGGGAAAAGATTGTTATCTGGTCATTGGAATGTGATTGATGTATGTAATGGCTTATTAGGGGGTTTTGCTGCGATCACCGCAGGGTGTTCGGTGGTGGAACCGTGGGCTGCAATCATATGTGGCTTTGTTGCAGCTTGGGTTTTAATTGGGTGTAATAAGCTCGCAGATAAACTACAATATGATGACCCATTGGAGGCAGCCCAATTGCACGGCGGATGCGGCATGTGGGGGTTGCTGTTCACCGGGCTGTTTGCGAAGGAGACGTACGTGAATGAAGTGTATTCAAACAAGCCGGGGCGGCCGTATGGGCTGTTCATGGGTGGTGGTGGGAAGCTGTTGGCGGCCCAGATAATTGAAATCTTGGTGATAGTAGGGTGGGTCTCGGCGACAATGGGCCCACTGTTCTATGGGCTCCACAAGTTAAAGTTGCTGAGGATCTCAGCTGAGGATGAGATGGCAGGCATGGATTTGACAAGGCACGGAGGGTTTGCTTATGCATACGATGAGGAGGATGATGTGTCAGGAAAGCCTTCTTTTATGATGAAGAAAGTGGAGCCTGCGAAGAACACCTCACCTAATGGAAACTCACCAGCTATCAATGTGTGATCAAGTTTGATGCTTTACAATGCCATCAAGTAAATAAATAACCAAAAAAAAAAAAAAAACTTGGATAAATAGTATTAAAAAAAAGAGAGAGAAGAAGCCAGGCATAATTGGCATATCCAAGCTTGAGTCGTTAAGTCGTTGTATCAACTCGTAATTTTACGAGATCATCTTAAGTTTGTTATTTTTGTTTAAATCAA
