# Supplementary material for: Identification of Populus Small RNAs Responsive to Mutualistic Interactions With Mycorrhizal Fungi, Laccaria bicolor and Rhizophagus irregularis
Source: Front Microbiol. 2019 Mar 18;10:515. doi: 10.3389/fmicb.2019.00515 (PMC6431645; doi:10.3389/fmicb.2019.00515)
Supplement: Supplementary file 3 [file Table_3.DOCX]

*Populus deltoides* miRNA precursor sequences

>Pde_miRNA_1

AAAAATTCCGTCGGTAAAACTGTTATCTGTCGGTATATTTTAGAGAGTTGGAAAAACATTACTGTATATGCCACTATCACCGATGGAAATATACTAACGGAATTTTTCCAGGGAATTTTTTT

>Pde_miRNA_2

AAAAGTAAGTAGGATTTCTGTTTATGAATTGAAGGATAGTTTAAATGTTTAAAATTATAAAAATAATTAAAAAATAAGTAGGATTTCTGTTTATGATACT

>Pde_miRNA_3

AAAAGTTGGTCAACGGAAAACACTTTCTGGTCAACGAAAAATACTTTCGAGTCAAAGAAAAATTTGACTTGGTTTCCAGGAAAGTGTTTTTCCTTTTGGTTGTGTTTGTTTTCCGAAAATGGTTTA

>Pde_miRNA_4

AAATCACCGACGGATTGAAAAGTCGTTGGTGATATTTAGAGGGTTTTTAATTTTTTTAATTAAATTAAAAATTTAAATTAAATATTACAGATGAAACACCGACGGAATGGTTTAA

>Pde_miRNA_5

AAATTAATTACCGATGGAATATGTGTCTTACGCAAATAAAAAAATTCCGTCGGTAAAACTGTTAA

>Pde_miRNA_6

AACGGTCGGATCAAAAGTTATGGCCCTTTCAACCGTTACTATGGTCTCGAGCGAAGA

>Pde_miRNA_7

AACGGTCGGATCAAAAGTTATGGCCCTTTCGAACCCATACTCTAGTCTCGAGCGACGA

>Pde_miRNA_8

AACGGTCGGATCAAAAGTTATGGCCCTTTCGAACCGGTACTCTAGTCTCGAGCGACGA

>Pde_miRNA_9

AACGGTCGGATCAAAAGTTATGGCCCTTTCGAACGAGTACTCTGGTCTCGAGCGACGA

>Pde_miRNA_10

AACGGTCGGATCAAAAGTTATGGCTCTTTTCAGCTGTTATTTGGGTCCGACGCAACA

>Pde_miRNA_11

AACTCGAGATATGGGCTGAACACTGAACAATGTCTGGGTTGTAGGACAGATTCTGACTTCTCTATTGTTTCTACAATTTGAATTTGAAAACAGCCTTTTTGAATCTTAGACTCTTCATGAAAGTATTAGGTCTATGTCTTAGCTTT

>Pde_miRNA_12

AAGACCACTACGCACGAAGGACCAAGGTGGATTCAGGAGGATAAGGGTAAGAAGCGGGGTAGAGTAATGGTTAACT

>Pde_miRNA_13

AAGAGAACGTGGATCTGACGATCTGTCAGACTCAACATTTTATGGTTCAACTACATATTGACCCAAGCTAACA

>Pde_miRNA_14

AAGATCTGTGCACTGTGAAGAGAAAACTGAATGTGAAAAAGAAAGCCTTTTTAGCCGAACAAGTAAGTGCCATTCTTCAAAACAATAATGCTTTGAAATATAAAGACCCTGGTTGTCCTACAATTTCTTGCTTTATTGGAGAACATAAAATTGAAAGAGCTTTACTTGATCTTGGAGCTAGTGTGAATTTACTTCCATATTCAGTCTTTCAGAGTCTCAATCTAGG

>Pde_miRNA_15

AAGATCTGTGCACTGTGAAGAGAAAACTGAATGTGAAAAAGAAAGCCTTTTTAGCCGAACAAGTAAGTGCCATTCTTCAGAACAATAATGCTTTGAAATATAAAGACCCTGGTTGTCCTACAATTTCTTGCTTTATTGGAGAACATAAAATTGAAAGAGCTTTACTTGATCTTGGAGCTAGTGTGAATTTACTTCCATATTCAGTCTTTCAGAGTCTCAATCTAGG

>Pde_miRNA_16

AAGCTCAGGAGGGATAGCGCCATGAGCATGACAAAGTCTATGTTTGAGTTAATCTCAACAAAATCAATCCAGTCATCAGTGGCGCTATCTATCCTGAGTTCTA

>Pde_miRNA_17

AAGCTCAGGAGGGATAGCGCCATGAGCTGATGATAAGTTGATGTTTGATGGGTTAATCTCAACATAATCAATCCAGTCATTAGTGGCGCTATCTATCCTGAGTTCTA

>Pde_miRNA_18

AAGCTCAGGAGGGATAGCGCCCTAAGGATAACCACGGGCTCTTTTTATTTGGTTTTTGACTATCAGTGGCGCTATCCATCCTGAGTTTTA

>Pde_miRNA_19

AAGCTCAGGAGGGATAGCGCCCTAAGGATAATCATGGGCTCTTCTTATGTGGTTTTTGATTCTCAGTGGCGCTATCCATCCTGAGTTTCA

>Pde_miRNA_20

AAGGACTAACTTGTAAAAGGCGCCGAAATGCAGGGCCAATTACAGTTTAAACCAGGG

>Pde_miRNA_21

AAGGCAACAAATTAGAGTCTCTGAAGTGCTTGAAATTTCAATAGCGTTTTCAGTGCTGTACGTTGCCGACACCAATTTTGGGTGCTGAGATTCTGGCATTTGCTTCCC

>Pde_miRNA_22

AAGGCTCTGATACCATGTTAAAAAATCATCTTAACCTAAAATCAATTAAGCTTTTAAGTTTTTAAGTTGAGATGGTTTTTTGACATGGTATTAAAGTTTGTT

>Pde_miRNA_23

AAGTAAGTAGACATATTGATAAAGTGTTGAATGCACAAACTGTTGAAGAAGTTCAGAAAAATCGGTTAAGACTTATGACAACAATTGAAAGTGTTCGATGGCTTAGCTTACAAGCATGTGCATTTAGAGGT

>Pde_miRNA_24

AATCACTACTTCTGAATTGGGACCTGATAGGGATACTATAAAAGTCCTGACGATTCTTCCACTGATTCT

>Pde_miRNA_25

AATGCTGTCTGGTTCGAGACCATTCACCTGAAGCGCACGCATTCATCTTTTGAGTGATCTCGGACCAGGCTTCATTCC

>Pde_miRNA_26

AATGGCCATTGTAAGAGTAGAAGGATCCATGAAGCAAAACATTGACTCCTGATATTCCACTCTTACAGAGTCCATTGA

>Pde_miRNA_27

AATGTAATTCGGTTCGGTTGGTTTTTTGAGTTTGAACCGAACCGAACCGTGAACACCCC

>Pde_miRNA_28

AATGTTGACCGAATATGGATGAAAAGTTGTTTTCTGTTTCCTTTCCTCCACATTCGGTCAATGTTCC

>Pde_miRNA_29

ACCAAATGCCTCGTCATCTAATAGTGACGCGCATGAATGGATT

>Pde_miRNA_30

ACCCAAGTTTAAGTGAGTCTGGCTGTAATACCGGACCCAAGAATACTGGATGTGGGTCT

>Pde_miRNA_31

ACGTACTAATTTTGATTTTTGAAGTTTTCCCATGCATGGAGGTTGTGGAAAGAGGAAAAGGTACTGTA

>Pde_miRNA_32

ACTCATCATCTTTCTCACAAGGTTCAAGATGAGCTTTTCAATAACCTTACCACTACGAAGAGTGATGATTG

>Pde_miRNA_33

ACTCATCATCTTTCTCACAAGGTTTAAGAATGGGTTTTTCAATAACCTTACCACTGCGAAGAATGATGACTAATTTGGCTTGACCCATGTGTTGGCTTCTAGAACTACTTGCATTTGCATTGTATTGCCCCTTTGGATTTTGCTGTGGTTGAGATGGAAACTTACCTTTCTCTTAAAAACTGAGAGCAGATGTCA

>Pde_miRNA_34

ACTCCCCCTCAAGGGCTTCCTGTTTGCCCAATCTGGTAATGATGGTTTTGTGTTTTAAGACAAAACATTTGCTACCAAGTATCGAGGCAGGCTGGGGGCTCTTTTGGGGGTTTTA

>Pde_miRNA_35

ACTCTCCCTCAAGGCTTCCAACGCAATAACCAGCTACGTACATGTAACTGTGTTACGGCTGCAGGCTTGAGGCCTTTGGGGGAGAGTGG

>Pde_miRNA_37

ACTTGGTGGTTGCTTATATGATGGTCATTAGAGGTTTACGTAGTTGTTAATTTCAGGGTCCATAAGATTAGTCGAGATACACGCAAGCTG

>Pde_miRNA_38

AGAATCTTGATGATGCTGCATCAGCCATAAATGACTGTATATACACACCTGAAATAGTTGGATCTAAGATTTTCT

>Pde_miRNA_39

AGCATTTGTGCGTGCATGGACTCGCGGTGCGATCATACCAGCACTAATGCACC

>Pde_miRNA_40

AGCCATTTTATCAACGGACAACTTCAGATTTTTTTTTTTTTTTTGTCTTCCCTAACTGTTTCTTTCAAGGTAAAAAGACAATGGACGGACGTAGTAATAGTGGTCCCACCATTATCAGAGTTATCGTAGTTCATTGACACCCAGTGGTCGAAGGTTTTAAGGCAGTTTTTGAAAAATTATCTTTTAAATTTTTTTTGTATTTGTTTGTTATTAGAAAAGTTGGTTAA

>Pde_miRNA_41

AGCTATTGTCTTCCATGGAATAGGCAGTGATGGCGTTTGTTTTATTTTCCTGAGGTGGAGAAAAACAAGTAGACCATAACACTGTTATTCCATGGAAGATAATGACTCG

>Pde_miRNA_42

AGCTCCTTGAAGTCCAATAGAAGCTCCTGCTGGGTAGATCGAGCTGCTGAGCTATGAATCCCACAGCCCTATCACCATCAGTCATTTTGATGGGCCTGCGGCTTGCATATCTCAGGAGCTTTATTACCTAATGTTAGATCTTTTTTTGGATTGAAGGGAGCTCT

>Pde_miRNA_43

AGCTCCTTGAAGTCCAATAGAGGTTCTTGCTGGGTAGATTAAGCTGCTAAGCTATGGATCCACAGCCCTATCTATCAACTGAAGGATAGGTTTGCGGCTTGCATATCTCAGGAGCTTTATTGCCTAATGTTAGATCCCTTTTTGGATTGAAGGGAGCTCT

>Pde_miRNA_44

AGGCTTCTCTTCTTTGGCAGGTGATGGTATAGATTACAACTTATAGCTTTAATCTAGTATTCAGTTACCCGCCAAAGGAGAGTTGCCCTC

>Pde_miRNA_45

AGTAAGTAGACATATTGATAAAGTGTTGAATGCACAAACTGTTGAAGAAGTTCAGAAAAATCGGTTAAGACTTATGACAACAATTGAAAGTGTTCGATGGCTTAGCTTACAAGCATGTGCATTTAGAGG

>Pde_miRNA_46

AGTAGGATTTTCGGAAGGTTATCGGGTGGGCGAGCGGGGAAGATAACTTTGGTTTTTGAGATATTACTTGTTATTTTCCCTACTCCACCCATCCCATAGGTTTCCGATCATTCCTCC

>Pde_miRNA_47

AGTGTCTGGGCTGCAGGACAGATTTGGACTTCTCCGTTGTTGCTACAATTTGGACTTGAAAACGGCCTTTTTAAATCTTGGACTCCCATGAAAGTTTTAGGCCTATGTCTTAGCTTCCCATCCATATAAAACAGACCGAAATCCAAGATCTACAGCTCCAGATATGAC

>Pde_miRNA_48

AGTTCATTTTTTTATTGACGTTAGAACGATTAGATTTTTACCCGATAAGATAAGGACCTC

>Pde_miRNA_49

AGTTTGTTCGTGGATCTGTCGCCATCACAACCGTTCATTTCACGACCATCCAATGGCGTTAGATTCACGCACAAACTCG

>Pde_miRNA_50

ATAATATTCCTGACTCTGGCGCCAGTGAATATTCGAACTCGAATAATTCCAACTCTGGAGTTGGTGAAAATTCATA

>Pde_miRNA_51

ATAATATTCCTGACTCTGGCGCCAGTGAATATTTAACTACAGATAATTTCAACTCTGGTGTTGGTAAATATTACGT

>Pde_miRNA_52

ATAGTGTCAGAATCAGGAGCTGGAGAGACTTGGTGGAGGCCTTCCTTACGCAGTACAAGTTTAACCTGGAAATCGCTCCTGATCGAACAAGTCT

>Pde_miRNA_53

ATCAATAGTTCTGAATTTTGGCCTATAAAAGAAGGCATTTGCCATGTATTTAGGCGTCTTGGTGTTCAGATCAAGATCAAGATCATGCTCTTGCTCTTTCTTTATATTTTATAATGCTTAAGTTTTGCTTATATTAATTT

>Pde_miRNA_54

ATCACGAGCCATCATAACTGTAGGAATCATCGTGATCATCCCTAACAAAACATGATGATCATGATGATTCCTACAGTTATGATGGCCCATGATCT

>Pde_miRNA_55

ATCTGCCAGACCCAAGAAGCTCGAGTTCGGCAAGACAGCCAGACCCAAGGCGCCATGGGTCTGGGAGGTGC

>Pde_miRNA_56

ATGAAAAGGACTTTGAAAAGTAATTTTTATTATATGATAATAGTTATTTTTTAAAATGTTTTTATTT

>Pde_miRNA_58

ATGGTGGGAGAGAGCTTCCTTCAGCCCACTCATGGATAGGAGAAAGGGGTTGAATTAGCTGCCGACTCATTCATTC

>Pde_miRNA_59

ATTATGTGCAGGGAGTTGACCTGATCTGTTTTCGTGTGGTTAAAAGAAGTTCACAAGGGAAAAGGTGAAGCTGCCAGCATGATCT

>Pde_miRNA_60

ATTCTATGGTACGAGACTGGTTACGCAAAAAAAAAAAATACTATCATCTCTTAAGCATCCTTACCTAAGGTATGCTGCATTGCTGGTTTTGTCTTAAATTG

>Pde_miRNA_61

ATTTGAGTTTTGTAGGCGTTAGCTATCATTATCATTTGTTAGAGGGTATTTAGGATGTTACCTTTCAACAAAAGACAATGACAGCTAACACCTACAAAACCCAAACAG

>Pde_miRNA_62

ATTTGCACGTCAGTCTGATCCTGAAATTACACTGATTATGACTATTTTTCTACTTTTCATTCTCATTGGGATTTCTACGTGTGTTTTGA

>Pde_miRNA_63

ATTTGGACTTCTAGAACTCGAGATATAGGTTGAACAGTGTCTGGGCTGCAGGATAGATTCTGACTTCTCCATTGTTGCTAAGATTTGGACTTCCAAACAATAGAATTGAGTCTTGCACTTCCATGAATGTTTTAGGCCTATGTCTTAGCTTTCTAGCCATATAAACCA

>Pde_miRNA_64

ATTTGGACTTCTAGAACTCGAGATATGACTTGAATACTAAACAATGTCTGGGCTGC

>Pde_miRNA_65

ATTTGGACTTCTAGAACTCGAGATATGGGTTGAACACCAACAGTGTCTGAGCTATAGGACAGATTC

>Pde_miRNA_66

ATTTGGACTTCTAGAACTCGAGATATGGGTTGAACACTAAACAGTATCTAGTCTGCAGGACAGATTC

>Pde_miRNA_67

ATTTGGACTTCTAGAACTCGAGATATGGGTTGGACACTGAACAATGTCTGGGTTGTAGGACAGATTC

>Pde_miRNA_68

ATTTTAGGAAGGGAATGAATACGGTGTCGGTGTTATAAGAGATAAAATTATAAAATTTTGGCATCAATGTGTCTGTTTCATCAGGTGAATAA

>Pde_miRNA_69

ATTTTATTTTTTATTAGGCGTTTGTAGTCCAACGGTTAGGATAATTGCCTTCCAAGCAATAGACCCG

>Pde_miRNA_70

ATTTTTATTCCTGACTCTGGCGTCAGTGAATAAACCAATAAATTTACATTATTTTTATTCGTGCATACACATTTTTTATTTATTATTTCTGCTAAATAAAATAAAATACAACAAATAAAAATAATATAAATTTAAACTGGTATTTTTATTCCCGACTCTGGCGTCAGTGAATAAACCAAT

>Pde_miRNA_71

CAAAGAATCATCTCAACCCAATAGTTTAAGCTATTAGGTGAGATCCCAAGCAGTCATGTTAGAGAAGACCTCGTTTAATAGCTTAAGCTATTGGGTTGAGATGGTTCTTTGAC

>Pde_miRNA_72

CAAATTCTGATGTTAGCAATGATGTTTTGGCCATCCTGTCAAAAATCTGCCAACAATTTCAAGAACCAGCA

>Pde_miRNA_73

CAAATTCTGATGTTAGCAATGATGTTTTTTTCAGCCAACAATATCAAGAACCAGTA

>Pde_miRNA_75

CAATCATGGCTGAAGTGACACTTATGCCCCTTCTTTAAAATTCCTAGAATTCTAACCTTTATTTATATATATATATATATATATATATATATATATGAGAGAAAGTTCCAAACAATTAGGATTTTTTTTTTGAAGATTGGATACAGTTACTAATTCATGATCTGG

>Pde_miRNA_76

CACATTCGGTCAACGTTCGAGTGATGACAGAAAAAGATTGGATCTCTAGCAGCTGAGTAGAAATTGCCCATGTCTTCAAAGTTTGGTTGAGTGTTGT

>Pde_miRNA_77

CATGAAAGTTCTAGAGAATTGTCTCATCTTTCCAGGGTTAAAAAGCTGAGATCATTTGGACTTCTAGAACTCGAGAT

>Pde_miRNA_78

CATGAAAGTTCTAGGGAATTGTCTCATCTTTCCAGGGTAAAAATTTGAGATCATTTGGACTTCTAGAACTCGAGAT

>Pde_miRNA_81

CCACACATGTGATTGCTGGCTCCATTGTTAAGATATCACGTGTTTTTTT

>Pde_miRNA_82

CCATGAAGGTTCTAGAAAATTGTCTCAGCTTTTCAGGAAAAAAAAGATGAGGTCATTTAGACTTCTAGAACTCGAGATATGGGC

>Pde_miRNA_83

CCATGACATGAGTCCAAGTTCAGACCGTCTCATGACGACAGGCGGAGACACCTCAGGAGAAGGTGTGAGGACCATGATATGAGT

>Pde_miRNA_84

CCTAATGACGAACTATTTTGACAAGCTATGGACCTATGTCCAAATGAAGCTTCTGTCATAGTTACTCTGACAGGGA

>Pde_miRNA_85

CCTTATGAGAAATCAAAGTTTTTGGGCTATGGGCGAGAATGGTCGCAAGGATGAAACTTAAAGGAA

>Pde_miRNA_86

CGATGTTGGTGAGGTTCAATCCGAAGACAGATTTACACGAGTGAAAGTAAAATCCGATCTCAGATTGAGCCGCGCCAATATCACT

>Pde_miRNA_87

CGATGTTGGTGAGGTTCAATCCGAAGACGGATTTACACGTGAAAGTAATTGTAAAATACGATCTCAGATTGAGCCGCGCCAATATCACT

>Pde_miRNA_88

CGGGGAACAGGCAGAGCATGGATGGAGCTACTAACAGAAGTACTTGTTTTGGCTCTACCCATGCACTGCCTCTTCCCTGGC

>Pde_miRNA_89

CGTACTCTGGTCCGAGCGAGATACCTCCGCATCACGAGTAGCGATTCCACATCTACTCCTAGGAAAACGGCGTCGAAACTCCTGTAAAATTTGAGGGAGATCCAACGGTCGGATCAAAAGTTATGGC

>Pde_miRNA_90

CGTCCATCAGCAGCACTACTCAGTATTTTAGTCATATCTTGAGCTCTAGTGATCCAAATGCTCTGAAATGTT

>Pde_miRNA_91

CTCATCTTTCTAGGGTAAACATTTGAGATCATTTGGACTTCTAGAACTCGAGAT

>Pde_miRNA_92

CTCTAGCGTCAGTGAATAAATCAATAAATTTATTTTATTTTTATGTTATTTTTTTTCATGCACATTCTTTATTTTTCTAGCTAAATAAAATAAAATACAATGAATAAAAATAATATAAATTTAAACCGATATTTTTATTCCTGACTCTGGCGTC

>Pde_miRNA_95

CTGCTCGCAAGTCGGAGGCCTGGCTGACGTGGCTGGTGCGACCGCCGAGCTTGGGATTGCGAGGAGAGCTCTACGCTGGCGTGGGCGTGGCATTAAATTGCCGTGCGCGCGCCCATGCGTTGGCTCTCTTGCAATCCCGAGCTCGTGCGTATGTGCCCGCTGCCGAGCCTGGCCTCCGTCTTCCGAGCCGGGG

>Pde_miRNA_96

CTTCATAAAAGTTCTAGGCGTTTGTCTCAGCTTTCCAACAAAATAAGAATGAGCTCATTTGGACTTCTAGAACTCGAGAT

>Pde_miRNA_97

CTTCGCGTCCAGCGGTGCGGGCACGATCTCGCTCGGGTGTTTCCACTTGCTCTCGTGGCCGTGGTTCGCTCGTCGGG

>Pde_miRNA_98

CTTGAATCATGAAAGTTCTAGGAAATTGTCTGAGCTTTCCAGGAAAAAAAGTTGAGGTCATTTGAACTTCTAGAACTCGAGATATGGGC

>Pde_miRNA_99

CTTTGTGTATGGTCTTTGGTTCCCTTGTTGGTTTCTGGTTTGATATCTCCTTCCTGCTGACATGATCAAAGAGTTGATGTTTTATCCCAAGTGCAGGAGTGTCACATTAATAAATAACCCGGCAAGACCGGGGTCGAACCACAGAGAG

>Pde_miRNA_100

CTTTTTGGATCGTTTTGATGTGCTGATATCAAAAATAATTTTTAAAAATAAAAAATATCATTAACATGTATTTCAACAAAAAACTATTTAAAAAGCA

>Pde_miRNA_101

GACAAAAATGGCATAAGAGAGGTACAGTGGGAGGCAGGAGAAAGACCAATATCTCTGCTACCCTTCTTTTTCTCTTATACGTTTTTGTCTC

>Pde_miRNA_102

GACAAAAATGGCATAAGAGAGGTACAGTGGGAGGCAGGAGAAAGACCAATATCTGCCACCCTTCTTTTCTCTTATGCGTTTTTGTCTC

>Pde_miRNA_103

GACATTCCCACAGCAAATATGTACAGGCTCTCAGGTGTTTCCACTTGATCTCGTGGTCGTGGTTCGCTCGTCGGGATTGTTGT

>Pde_miRNA_104

GAGATTCCAAATCGACTCAAAATATTTTCTTTTAAAAACTTTATTACTGTTTTGTGATCATTGTTTCGACTTGGAATTGCCT

>Pde_miRNA_105

GAGCTCTCCCCACTCCATGCCTGAAAGGAGTTCGATGGTAGACCATGGCTGCTAGTTCATGAATACCCTTGGGTGCGCAGAATTAGAAACGGTGCAGGCGAAGTTGCGCAGGCTAAGGGTCTGCATGACCTAGGAGACGTGGTTACCCTGACCCTTTTTGTATTGGAGTGAAGGGAGCTCGA

>Pde_miRNA_106

GAGCTTCCTTCAGTCCACTCATGGACGGGCGAAGGGTTTGGATTAGCTGCCGACTCATTCATTCAAACACAGTAGACAAGGAGTGGCAGCGGCTGCTATTGTGAATGTGTGAATGACGCGGGAGATTAATTTCATCCTTTTCTTCTCTGTGCTTGGACTGAAGGGAGCTCCC

>Pde_miRNA_107

GATGCTGTCTGATTCGAGACCATTCACTTTAAGCACACATTCATCTTTCGAATGATCTCGGACCAGGCTTCATTCC

>Pde_miRNA_108

GATGGGTGAGTGGGGAAGATAACTAAGCTGTGTTTGTTATTTTCCCAACTCCACCCATCCC

>Pde_miRNA_109

GCAGCATCATCAAGATTCACATTCAAACAGATGTACGGCAGCTAGCTAGCTAGATAGATAGACAGCAATGTATTTCTTTGAAGGTGAGAATCTTGATGATGCTGCAT

>Pde_miRNA_110

GCCACCCATTCATCAATTAGTCAACAAATCAATAGTTCTGAATTTTGGCCT

>Pde_miRNA_111

GCCACTCACTCATGAATTAGTCAACAAATCAATAGTTCTGAATTTTGGCCT

>Pde_miRNA_112

GCGGCAGCATCAAGATTCACAAACTTTAAGGCTTGAGTTGGGGTGGTACACGGTCACCTCCTTTACTCGAAAGGTTCCTTAATTTCTGGTGGGAATCTTGATGATGCTGCAT

>Pde_miRNA_113

GCTATGATGAGTATGAAAAGGTTATGAGAGTTTTGATGAAATTCATTCCTGAGCGGTTATCTGAGCAA

>Pde_miRNA_114

GCTCAAGCACAAATTCGATCTTGCTTAACAAGGTACCGGCAAAGTCAGATCGAATTTGGGCTTGAGATT

>Pde_miRNA_115

GCTTGATGGGTTGTTTTAAGCTTTGATGAAAGGCTTGATAAATCATTCCGGACC

>Pde_miRNA_116

GCTTGCTTGTTTTCTTTCCCCTTTTTTCCAGCTTCTGGGACATTAGAAATTGACAGAAGAGAGTGAGCAC

>Pde_miRNA_117

GCTTGCTTGTTTTCTTTCCCGTTTTTTCCAGCTTCTGGGACATTAGAAATTGACAGAAGAGAGTGAGCAC

>Pde_miRNA_118

GCTTGCTTGTTTTCTTTCCCTTTTTTCCAGCTTCTGGGACATTAGAAATTGACAGAAGAGAGTGAGCAC

>Pde_miRNA_119

GCTTGGGCGAGAGTAGTACTAGGATGGGTGACCTCCTGGAAGTCCTCGTGTTGCAC

>Pde_miRNA_120

GCTTGGGCGAGAGTAGTACTAGGATGGGTGACCTCTTGGGAAGTCCTCGTGTTGCAC

>Pde_miRNA_121

GGAATGTTGGCTGGCTCGAAGCTTAAGCAAAGAGTATCCTAACATGAAACAACTGTTAAGGCTTCGGACCAGGCTTCATTCCCC

>Pde_miRNA_122

GGAATGTTGTCTGGCTCGAGGACTTTTTCTTGATCAATCTAATCGAACTTTCTACCTGTAGATCTAGTATCTTATTTAAGATTGATCACGTATTAGGGTTGTCGGACCAGGCTTCATTCCCC

>Pde_miRNA_123

GGAATGTTGTCTGGCTCGAGGTCACTAATGGGATCTATGATTTTATCTCAATTGATTGATTTTCTTTCAAATTCTAGTAAATTGAATTGAGAGATATCATGATCAACTTATATTTAATGATGTCGGACCAGGCTTCATTCCCC

>Pde_miRNA_124

GGAGCACCATCAAGATTCACAAACTTTATTAGGGCTAATAAGTGGTGATAATGGTGGCTTTTGGTGGTCCCTTCGTTTCAACCCAATAGCCATTTGAATTGGGAATCTTGATGATGCTGCAG

>Pde_miRNA_125

GGAGCATCATCAAGATTCACAAGCTTTTTTAGGGCTAGTGTGTGGTGATGATGGTGGCTTCTGGTGGTCCCTTTTTTTAATCCAATAGCCCTTTGAATTGGGAATCTTGATGATGCTGCAG

>Pde_miRNA_126

GGAGCATCATCAAGATTCACATGCAAATGCACGGCCGGTGATGTTAGAGTTAAATCTCTCTTTGTTTCTGTTCATCTGCCAAAGTTCTTTGGAAGTGAGAATCTTGATGATGCTGCAT

>Pde_miRNA_127

GGAGCGGCCTCGGGTCACATGTGGCGGCATCCAATGTACTTCATGTGTTCTCAGGTCGCCCCTG

>Pde_miRNA_128

GGAGCTATCGATGCTGAATAATCATTTGCACGTCAGTCTGATCCTG

>Pde_miRNA_129

GGAGGCAGCGGTTCATCGATCTCTTCCTGGCCAATTTTTTGTTTAGCACGAAAAACATGAACCGATCGATAAACCTCTGCATCCAG

>Pde_miRNA_130

GGAGGCAGCGGTTCATCGATCTTTTCCTGAAGATTTTTTTGTTTTACACGAACAACACGAACCGATCGATAAACCTCTGCATCCAG

>Pde_miRNA_131

GGAGTGGCTCCTGAGAACACAGGGGGTGTTGGTTTTCTAGCTGCAAGCTACAAGATGGACAAAGCACTCTGTGTTCTCAGGTCACCCCTT

>Pde_miRNA_132

GGATTCCAAACCGACTCAAAATATTTTCTTTAAAAAATTTGATCACTGTTTTGTGATTATTGTTTCGACTTGGAATTGCCT

>Pde_miRNA_133

GGATTCCAAATCGACTCAAAATATTTTCTTTCAAAAACTTGATCACTATTTTATGATCATTGTTTCGACTTGGAATTGCCT

>Pde_miRNA_134

GGATTCCAAATCGACTCAAAATATTTTCTTTCAAAAACTTTATCACTGTTTTGTGATGATTGTTTCGACTTGGAATTGCCT

>Pde_miRNA_135

GGATTCCAAATTGACTCAAAATATTTTCTTTTAAAACCTTGATCACTATTTTGTGATCATTGTTTCGACTTGGAATTGCCT

>Pde_miRNA_136

GGATTCCAACCGACTCAAAATATTTTCTTTCAAAAACTTGATCACTGTTTTATGATCATTGTTTCGACTTGGAATTGCCT

>Pde_miRNA_137

GGATTCCATATCGACTCAAAATATTTTCTTTCAAAAACTTTATCACTATTTTGTGATCATTGTTTCGACTTGGAATTGCCT

>Pde_miRNA_138

GGATTCTAAACCGACTCAAAATATTTTCTTTCAAAAACTTGATCACTGTTTTATGATCATTGTTTCGACTTGGAATTGCCT

>Pde_miRNA_139

GGATTGCTGTCTGGTTCGATGTCATTCATGTGAAGCTTTAACATTAATGTAGTATTGAGTGATTTCGGACCAGGCTTCATTCCCC

>Pde_miRNA_140

GGATTGTCGTCTGGTTCGATGTCATTCATGAGAAGCTCAAACATAAACGTAATATTGAATGATTTCGGACCAGGCTTCATTCCCC

>Pde_miRNA_141

GGCATGAGGTGTTTGGCAAGAAAATGGATCTTTTCCTTATGATGATTTCTTACCAATACCTCTCATGCCAA

>Pde_miRNA_143

GGTGGGCGAGCGGGGAAGATAACTTTGGTTTTTGAGATATTACTTGTTATTTTCCCTACTCCACCCATCCC

>Pde_miRNA_144

GGTTTGTGCATGAATCTAATATAGCTAAAAAAATCCCATCAAACACAAATTAAAATACTAGATAGCAATCAGCACTAGTTTAGTATAGTATTAGATTCACGCACAAACTCG

>Pde_miRNA_145

GGTTTGTGCGTGGATCTGAGGCCATCACAACCGTCCACTGCACGACCACCCAATGGCTTTAGATTCACGCACAAACTCG

>Pde_miRNA_146

GTATTAGATTTTCCTCTGGTATCCATGTTGTAACCAGGCTCTGATACCA

>Pde_miRNA_147

GTCTCAGCTTTCATTGTTTTTTTTTAATTACTGTGCTGCCAGCCAATGATGCAAACCCTGAGTTTCACTGAAAGCAGGGCTATCGATCTGCCTAAACTGTTGTTAATTCATAAACTAGGACGGTCTGAGGCTT

>Pde_miRNA_148

GTCTCAGCTTTCATTGTTTTTTTTTAATTACTGTGCTGCCAGCCAATGATGCAAACCCTGAGTTTCACTGAAAGCAGGGCTATCGGTCTGCCTAAACTGTTGTTAATTCATAAACTAGGACGGTCTGAGGCTT

>Pde_miRNA_149

GTGCAATGATATTTTAGAGAAATAGATTTTAAAAATATTTATACTAAAACTATTAAGAATGAAGAGATTGTGAACCT

>Pde_miRNA_150

GTGCTTATCGACGGTCTTGTGCTGGCTAGTTGTCTTCCACCAGCACACAACATATTCGTAAGCTACAT

>Pde_miRNA_151

GTGGTATTGATCCGGCTCTTCTTCTGTATTTGCATCAGAAGTTCTTCGCTGAAGACGAGCCGAATCAATATCACTC

>Pde_miRNA_152

GTGTATTATCATGTTGCGTGTTTCCAGTAGATGCGATGATGATAGAACAGTA

>Pde_miRNA_153

GTGTGTTGCGCAGCGAAATTATGTTTCGCTGCCGCAACTGAACCT

>Pde_miRNA_154

GTTAAATAATATTTATATAGTCTTATTTATTAATGATAGAATAGTACTTTCAGTTTAACCTATATATACTTTATTTGTATTTAGGTTAAGTTTAAGCACTCATAATATACAGATTATTCAGAATTCTAGCCT

>Pde_miRNA_155

GTTAAATAATATTTATGTAGTCTTATTTATTAATGATAGAATAGTACTTTCAGTTTAACCTATATATACTTTATTTGTATTTAGGTTAAGTTTAAGCACTCATAATATACAGATTATTCAGAATTCTAGCCT

>Pde_miRNA_156

GTTTAATATCTGATACGTGGGCAATGGCCACACGATATTAAATTT

>Pde_miRNA_157

TAAGAATGAAGAGATTGTGAACCTTGAATTTAAAAGATTTATAGGGTGTTTATAACTCATGAGTCTTGTC

>Pde_miRNA_158

TAATCTGCATCCTGAGGTTTGGATCACCACATGTTTTGATCTAGTCCTTGGGTTGCAGATTACC

>Pde_miRNA_159

TAATCTGCATCCTGAGGTTTGGATCATCATGTAGTTTGATCTAGTCCTTGGGTTGCAGATTACC

>Pde_miRNA_160

TAATGGTGGTCGGTCGTCTCTGATTGAGCCGTGCCAATATC

>Pde_miRNA_161

TAATTGATTATTGATGTAGTGGCCAACTATTATTTAGTGGACTTGGTGGACAGCAGTACTCAAGCATT

>Pde_miRNA_163

TACTTCTATTTCAGCTTCTTCTATATCTGAAACCTCAGAGAACAAGAGGATTGTACAAAGTGAAGAAAT

>Pde_miRNA_164

TAGAGAGAGATGCTCTCGCCGACTAACTGAGAGCTCTTTCTTGATT

>Pde_miRNA_165

TAGCCAAGGACGACTTGCCTATTTCCTCCATGGGGTTCTGAAGAGAATGATATATTGTCGTTCAGAGCTCATTGGTAGGGTTCATAGGCAGTCTCCTTTGGCTATC

>Pde_miRNA_166

TAGGCTGGAAACCTGTGACCCGATCTTCTAGATCATTCTCAGGTATAACAACACTACG

>Pde_miRNA_167

TAGTATTTTGGTCACTAGGAACCCTAACTAGTCTTAGAGATCGGTACAGG

>Pde_miRNA_168

TAGTATTTTGGTCACTAGGAACCCTAACTGGTCTCACAAATCGGGTACAGGGATTGGTTGCGTAAATAAAAGGTACTAGC

>Pde_miRNA_169

TAGTATTTTGGTCACTAGGAACCCTAACTTGTCTTAAAGTCTGGGTAAGGGGACTAATTGTGCAAGG

>Pde_miRNA_170

TATATTAAGGATGGTCTGGGTAAGGAGTCTTTAGATAATCTTCTATTAACTCTTGAGACGCATCCTAGTGGAGA

>Pde_miRNA_171

TATGGGAGAGGCGGGAATGACTCTGAAAGAACTTAGAAGAGTCTTTCCGAGTCCTCCCATACC

>Pde_miRNA_172

TATGGGAGGATTGGACAGTGCTGCTTGGTTTTAATTAACCAAAGTCTGTTCTTTCCAACGCCTCCCATACC

>Pde_miRNA_173

TATTCCGTCGGTAATTAATTTACCGATGACATCACCGATGGAAATGATCCGTCGGTGAATCCTTCATCGGTAATTGTTTATCTGTCGGAAATAA

>Pde_miRNA_174

TATTGGTACGGTTCAATCAGAAAGTAATGCTCCCAAGAGTATAGAGTACTATTGTTTGATTGAGCCGTGCCAATATC

>Pde_miRNA_175

TATTGGTGCGGTTCAATCAGATAGTAATGCTCCATAAGCATAGAGCTCTATTGTTTGATTGAGCCGTGCCAATATC

>Pde_miRNA_176

TATTTTTTTACCGATAAAATTAGCCATAAATTATGGAATTACCGATGAACGGTGTACTGATGGACGAATTTCATTAGTGAATTCGTCGCTACAATTTTCACCGATAGAATGGTAATCTCATGCTAACAGAAAAATTCCGTCGGTAAAACTGTT

>Pde_miRNA_177

TCAACTAGGTTACTTGTGTAATCTGACCTGAGCCATTGAAGACAATCATTTGTTGGTGTCTCATTGGATTTGTTACCTGACAGTGTTTATCCAACAAATGATTCTCTTCAATGGCTCGGTCAGGTTACACAAGCGATCTAGTTGGCT

>Pde_miRNA_178

TCAAGTGATGAATTTGGATTACAAATTTCTCACGACGGTCGTCTGAGACACATCGTGTCAATTTTGATGTAATGATCCAATGTTTTCTGATGATT

>Pde_miRNA_179

TCATGGGATGAAGAAAAAAACTGCAACAAAATGATCGATTAATTTCGTGATTACTTGTATTTAAGTCTCTGATGATT

>Pde_miRNA_181

TCCAAAGGGATCGCATTGATCTAATGACTCTCGATGTCTAAATCATGTTAATGTTTAGTTTTTTCGTTGGATCATGCGATCCCTTAGGAAT

>Pde_miRNA_182

TCCAAAGGGATCGCATTGATCTAATGACTTTTGATGTCTATATGATGTTAATGTTTAGTCATTTCATTGGATCATGCGATCCCTTAGGAAT

>Pde_miRNA_183

TCGCTTGGTGCAGGTCGGGAACTGATTCAGCGATTTGATTGCCAGATGGCTAAACACGATTGGCTGTGAGGCAAATTATAAAAAGAAAGAGAATTGGATCCCGCCTTGCATCAACTGAAT

>Pde_miRNA_184

TCGTAATGCTTCATTCTCACAACGGCAATGTGTTATTGCATCATCCTAATGATTATCATCAGCCGTTGTGGGAATGAACATTATGAGA

>Pde_miRNA_185

TCTAATGATGATCAAAACTTTCGTTCTTCTGACAATTTTAAACCATGTGGAAGATTTTATGGAGACCTGAACTGAAGAGA

>Pde_miRNA_186

TCTGTCGCTGGAAAGATGGTACCTAGGGCTGCATATGAATGTGTATTGCAAGCAATAGGCCCCATCTTCCGCGACAGAAC

>Pde_miRNA_187

TCTTTTTCCCCATAAAATTTCAGAGCATTTGGATCACTAGAGCTCGAGATATGGCTAAAATACTG

>Pde_miRNA_188

TGAAAGTTCTAGGAAATTGTCTCATCTTTCTAAGGTAAAAATTTGAGATCATTTGGACTTCTAGAACTCGAGAT

>Pde_miRNA_189

TGAACAATGGCCATTGTAAGAGTAAGTGGTCCGTGAGTCAACAACTCTCTTTGCAGAAATGCATGACAAAGCATTGACTCCTATTTCACCCTTACAATGTCCATTGATTAAG

>Pde_miRNA_190

TGAAGCTGCCAGCATGATCTAAATTAACTTCCTTCTCTATCAAGGATGGATTAGATCATGTGGTAGTTTCACC

>Pde_miRNA_191

TGAAGCTGCCAGCATGATCTAACTTCCTTGCTTCTTTATCAAGGATGGATTTAGATCATGTGGTGGTTTCACC

>Pde_miRNA_192

TGAAGCTGCCAGCATGATCTATCTTTGGTTAGAGAAAGAAAGGACTAACCCTAGCTAGGTCATGCTGTGACAGCCTCACT

>Pde_miRNA_193

TGAAGCTGCCAGCATGATCTATCTTTGGTTAGAGAAGGATAGAAGCGAAAGAACTAACCCTAGCTAGGTCATGCTCTGACAGCCTCACT

>Pde_miRNA_194

TGAAGCTGCCAGCATGATCTGAGCTTTCCTTAATTTTCCTATACGGGAAAGACTAGATCATGTGGTAGTTTCATC

>Pde_miRNA_195

TGAAGCTGCCAGCATGATCTGAGCTTTCCTTAATTTTTCTATACGGGAAAGACTAGATCATGTGGCAGTTTCACC

>Pde_miRNA_196

TGAAGCTGCCAGCATGATCTTAACCTCCCTCCTTTGTCGAGGAAAGAACAGATCATGTGGCAGTTTCACC

>Pde_miRNA_197

TGAAGCTGCCAGCATGATCTTAGCCTTCCTCCTTTGTTGAGGAAAGAAACAGATCATGTGGCAGTTTCACC

>Pde_miRNA_198

TGAATGTTGTCTGGTTCAAGGCCTGGCCACCACATCTCTTGGTGAATATGTCCTCGGACCAGGCTTCATTCCCC

>Pde_miRNA_199

TGACAGAAGAGAGTGAGCACACACGAAAGCTAATTGTATGAAAGCATACCATTGCAGGGTGTGTGCTCACTTCTCTTCTGTCAGC

>Pde_miRNA_200

TGACAGAAGAGAGTGAGCACACACGAAAGTATATGGTATGAAGGCATTCCATTGCAGGGTGTGTGCTCACATCTCTTCTGTCAGC

>Pde_miRNA_201

TGACAGAAGAGAGTGAGCACACAGAGGCATATTTGTATAAAATATACCATTGCTTTTGCGTGCTCATTTCTCTTTCTGTCACT

>Pde_miRNA_202

TGACAGAAGAGAGTGAGCACACAGAGGCATATTTGTATAAAATTATACCATTGCTTTTGCGTGCTCATTTCTCTTTCTGTCACT

>Pde_miRNA_203

TGACAGAAGAGAGTGAGCACACAGAGGCATATTTGTATAAATCTATACCATTGCTTTTGCGTGCTCACTTCTCATTCTGTCAGC

>Pde_miRNA_204

TGACAGAAGAGAGTGAGCACACAGGGTACTTTCTTGCATGACATCCATGCTTGAAACTTTGCGTGCTCACTCTCTATCTGTCACC

>Pde_miRNA_205

TGACAGAAGAGAGTGAGCACACAGGGTACTTTCTTGCATGACGTTCATGCTTGAAGCTTTGCGTGCCCACCCTCTATCTGTCACC

>Pde_miRNA_206

TGATGATTAATTGACTGCAAAAGCAACTCTCTTCTTTTTTTAGGAGAGGAGTTGCCTTTGCAGTCAAGTAATCATCAAG

>Pde_miRNA_207

TGATTATTCATTTTACTTTGATTAGGTTTTTGTAAGGATTGTTTGATGCGGAGATGATGAATATGCACT

>Pde_miRNA_208

TGCAATCAGAATTTGGCAAAGTAGTCTTCATGAAAGTTGTTGGCAATC

>Pde_miRNA_209

TGCATTTGCACCTGCACCTTACTTGTTTCTTTCTTTTGTTTTTGACTCCAAAACCAAAAGTAAGTTTGAAACATTTTATAAAACAAGCTTGAGGTGCGGGTGCAGGTGCAGG

>Pde_miRNA_210

TGCCTGGCTCCCTGAATGCCATCTAGGAAGCTTGTCAAAGAGTGTTGGCAACCTTTCTATTTGGCATGAGGGGAGTCGAGCAGG

>Pde_miRNA_211

TGCCTGGCTCCCTGTATGCCACAAGCAAAGACCAATCTCTTCTGTCTTAGATTGGCTGCTGCTGGTGGCGTGCGAGGAGCCAAGCATA

>Pde_miRNA_212

TGCCTGGCTCCCTGTATGCCACAAGCAAAGACCAATCTTTTGTTTTAGATTGGCTGTTGCCGGTGGCGTGCGAGGAGCCAAGCATA

>Pde_miRNA_213

TGCCTGGCTCCCTGTATGCCACAAGCAAAGACCAATCTTTTGTTTTAGATTGGCTGTTGCCGGTGGCGTGCGAGGAGCCAAGGCATA

>Pde_miRNA_214

TGCCTGGCTCCCTGTATGCCATTTGCAGAGCCCAACGGATCTTCGATGGCCTCCGTGGATGGCGTATGAGGAGCCATGCATA

>Pde_miRNA_215

TGCCTGGCTCCCTGTATGCCATTTGCAGAGCCCACCGGTTCTTCGATGGCCTCCGTGGATGGCGTATGAGGAGCCATGCATA

>Pde_miRNA_216

TGCTGAAATCTTGAGACACAGTCACCCACAAAACATGATCACAAATATCTGGTCTTCGTAGAAGTTCATTATG

>Pde_miRNA_217

TGCTTGGTTCGTTGGAGTTGCCACCTAGTATTATGGTCACTAGAAACCTATGGTCTGCGAGAGTCTGAGTAAG

>Pde_miRNA_218

TGGAAGTTTTGGGAAATTGTCCAGCTTTCCAACAAAACAAGAATCAATGCATTTGGACTTCTAGAACTCGAGAT

>Pde_miRNA_219

TGGAGAAGCAGGGCACGTGCAAAATCCTGATGAAGTGCTTACACTTTGCACGCCTCTTCTTCTCCAAC

>Pde_miRNA_220

TGGAGAAGCAGGGCACGTGCAAAATCCTGATGAAGTGCTTACACTTTGCACGCGCTCTTCTTCTCCAAC

>Pde_miRNA_221

TGGAGAAGCAGGGCACGTGCAAAATCCTTCTCGGCTTCCAGATGCTGATGAAGCACTCTTTGCACGTGCTCCCCTCCTCCAAC

>Pde_miRNA_222

TGGAGAAGCAGGGCACGTGCAAGCTCTCTCTTCAAGCTTTCCTTGCACGTGCTCCCCTTCTCCAAC

>Pde_miRNA_223

TGTGCTATATCAAAAATGATTTTTAAAAAATAAAAAACATCATTGGCATGTATTTTGCATGAAAAGTTATTTGAAAAGCACCCG

>Pde_miRNA_224

TGTGTTCTCAGGTCACCCCTTTGGGGCACCCTGTGGACATGAC

>Pde_miRNA_225

TGTTATCAATTTCTGTCTGAGCCTATAATGTTGTTCAAGGAAGATATGCAATATTTTGTTTTTTGCTCATACATAATTTAGTAATATT

>Pde_miRNA_226

TGTTGATGTCAAAAATAATTTTTAAAAAATGAAAAAACATCATTGCATGTATTTTGACACGAAAAGTTATTTGAAAAGCACC

>Pde_miRNA_227

TGTTGGCAGAGCTCAATCAAATCAAAGCACTCAATGGCTGGGTTCTTTCATCTGATTGAGCCGTGCCAATATC

>Pde_miRNA_228

TGTTGGCCCGGTTCACTCAGAGAAAAGACAACATTTTTTTTCAAGAAGATCATCAAAGCTTGAAAGAATAATGGTGGTCGGTCGTCTCTGATTGAGCCGTGCCAATATC

>Pde_miRNA_229

TGTTGGGATGGCTCAATCATATCAAATCTCCCAAACTATGATGTTGGGTCGTTTAATCTGATTGAGCCGTGCCAATATC

>Pde_miRNA_230

TGTTGGGATGGCTCAATCATGTCAAATCTCCCAAATTATGATGTTGGGTCTTTTAATCTGATTGAGCCGTGCCAATATC

>Pde_miRNA_231

TTAAAACTCGAAATCGGTCAAACTCGGTTAAAATCAGTCAAATTCGTAAAATCGGTCTGATTTTACTGATTTTGACCGAGTTTGACCAATTTCAAATTTTAACC

>Pde_miRNA_232

TTAAAACTCGAAATCGGTCAAACTCGGTTAAAATCGATAAAATCAAACCGATTTTACGAGTTTGATCGAGTTTGACCGATTTCGAGTTTTAACC

>Pde_miRNA_233

TTAAAACTCGAAATCGGTCAAACTCGGTTAAAATCGGTAAAATCGGACCGATTTTACGAGTTTGACCGATTTTGATCGAGTTTGATCGATTTTAAGTTTTAACC

>Pde_miRNA_234

TTAAATCATGAAAGTTCTAGGAAATCGTCTCATCTTTCCAGAAAAAAAAAAGATGAGGTTATTTAAACTTCTAGAACTCGAGATATAGGC

>Pde_miRNA_235

TTAAGGCTCTGATACCATGTTAAAAAATCATCTTAACCTAAAATCAATTAAGCTTTTAAGTTTTTAAGTTGAGATGGTTTTTTGACATGGTATTAAAGTTTGTTGA

>Pde_miRNA_236

TTACCGACGGATAATTGAATATTAATATTTTTTAATTATTCCATCGGTAATT

>Pde_miRNA_237

TTACCGACGGATAATTGAATATTAATATTTTTTAATTATTCCGTCGGTATTT

>Pde_miRNA_238

TTACCGACGGATAATTGAATATTAATATTTTTTTAATTATTCCGTTGGTAATT

>Pde_miRNA_239

TTACCGACGGATAATTGAATATTAATTTTTTTAATTATTCCATCGGTATTC

>Pde_miRNA_240

TTATAAAAATGAGGACCAAAGTTAATAAAAAAATTAAATTTTAAGAGATGAAATTGAAAAATAAATATTCAAAACAAAATTTATATAGCAATCAAAAATTTGAGGACCAAATTTGATATAATC

>Pde_miRNA_241

TTCATTCCTCTTCCTAAAATGGCTTCTCCTTCAATTTGAAAAAAATAAAAATTTAAGGAGAATCCAATATGAAATGAGAAGCTATTTTAGGAAGGGAATGAATA

>Pde_miRNA_242

TTCATTCCTCTTCCTAAAATGGCTTCTTCTCCTTTAGTTGTTGCAATTATAGGAGAGGAGAATCCATTTTAGGAAGGGAATGAATA

>Pde_miRNA_243

TTCCACAGCTTTCTTGAACTGCACCTATTAGATTAATGTTGATGTTGTTGTGCGATATGCCATGACCATATGACATTGTATTCATTTTTGCTGCGGTTCAATAAAGCTGTGGGAAG

>Pde_miRNA_244

TTCCACAGCTTTCTTGAACTGCACCTATTTGAATATTGTTGTTGATGTTGCCGTGCATGTACATATGACATTGTATTTTTGTTGCGGTTCAATAAAGCTGTGGGAAG

>Pde_miRNA_245

TTCCACAGCTTTCTTGAACTGCACCTATTTGAATATTGTTGTTGATGTTGCCGTGCATGTACATATGACATTGTGTTTTTGTTGCGGTTCAATAAAGCTGTGGGAAG

>Pde_miRNA_246

TTCCACAGCTTTCTTGAACTTCCTAGAGGTGCTGCTATACATATAACATAAGAAGTTCAAGAAAGCCGTGGAATA

>Pde_miRNA_247

TTCCACAGCTTTCTTGAACTTCCTTGCCATGCTTAACTTGTGTGTGTCTGTATATAGATCACTACATATCTGTATAGTTATACACATTTAGCTAGCTAGCACCATGGAAGCTCAAGAAAGCCGTGGGAGA

>Pde_miRNA_248

TTCCGAGATTCTGGACAGAATTCAATATGAATTATGGGCAGAATTATTGTATA

>Pde_miRNA_249

TTCGACCGTTGATTGATGAGTTGACGTAGTTGTGGTCCTCCGGAGCTTTGACTTATGATATATCGAGAAAACAAAATTTCCTTATGAGGGCGGCTTTGATGTGGACTATCAATGATTTTTCAGCTTATGGAATGCTTTCTGGTTGGAGC

>Pde_miRNA_250

TTCGACCGTTGATTGATGAGTTGGTGTAGTTGTGGTCCTCTAGAGCTCTGACTTATGATATATCGAGGAAACAAAATTTCCTTATAAAGGCGGCTTTGATGTGGACTATCAATGATTTTCCAGCTTATGGAATGCTTTCTGGTTGGAGC

>Pde_miRNA_251

TTCGGCAGCGAATCACAGTTTCGCTGCACTACACACAACTCGGCAGCGAATGCTAATTCGCTGCAGAAGAC

>Pde_miRNA_252

TTCTGAACTCTCTCCCTCAACCGCCACTCTAGAGGTTGGCACCTTGTCAATTGTTTCTGTGTAGCGGTCGAGGTGAGGGGTTCAGAACA

>Pde_miRNA_253

TTCTGAACTCTCTCCCTCAACTGCTACTCTAGATCTTGCCAATCCCTGTCAAGTTTTTCTGTGTAGCAGTCGAGGTGAGAGGTTCAGGAAA

>Pde_miRNA_254

TTCTGAACTCTCTCCCTCAACTGCTATTCTAGAGCTTCCTGAGCTAACCCTTGTCAGGTGTTTCTGTGTAGCAGTCAAGGTGGGAGGCTCAGAAAA

>Pde_miRNA_255

TTCTGAACTCTCTCCCTCAACTGCTATTCTAGAGCTTGTTAATCCTTGTCAAGTGTTTCCCTGTAGCAGTCGAGGCGAGGGGGTTTCGGAAAA

>Pde_miRNA_257

TTGACAGAAGATAGAGAGCACAGATGATGAAATGCATGGAGCTTAATCGCATCTCACTCCTTTGTGCTCTCTAGTCTTCTGTCATCA

>Pde_miRNA_258

TTGACAGAAGATAGAGAGCACAGATGATGATATGCAATGGACTCTGCATCCCACTCCTTTGTGCTCTCTATGCTTCTGTCATCA

>Pde_miRNA_259

TTGACAGAAGATAGAGAGCACAGATGATGTTTTGCAGTAGACTCTGCATCTCACTCCTTTGTGCTCTCTATGCTTCTGCCATC

>Pde_miRNA_260

TTGACAGAAGATAGAGAGCACAGCTACATGTAATCGAGAATTAGTCCTGTAGAAGTGCAGTTTGTGCTCTCTTTTCTTCTGTCAACA

>Pde_miRNA_261

TTGACAGAAGATAGAGAGCACTGATGATGAAATGCATGGAGCTTAATTGCATCTCACTCCTTTGTGCTCTCTAGGCTTCTGTCATCA

>Pde_miRNA_262

TTGAGGACCAAATTTGATATAATCAGCAAACAATATGACATTTCTAATTTTTTCACAA

>Pde_miRNA_263

TTGAGGACCAAATTTGATATAATCAGCAAATAATATGATATTTTTAAATTTTTCACAAC

>Pde_miRNA_264

TTGAGGACCAAATTTGATATAATCAGTAAATAATATGACATTTTTAAATTTTTCATAAT

>Pde_miRNA_265

TTGATTGATTGATTGATTGATTTTGCGCTTTCTTGCAGGTAAACATTGATTTTAATTGAATTCAAGAT

>Pde_miRNA_266

TTGCGGTTGCCTTTCAAAGTACTTTTCATTTTGAAATGCATCAAAATGATGTTTTTTCATTTTAAAAAAATTATTTTTGAGATCAGCGCATC

>Pde_miRNA_267

TTGCTTGCTTGTTTTCTTTCCCCTTTTTTCCAGCTTCTGGGACATTAGAAATTGACAGAAGAGAGTGAGCACAC

>Pde_miRNA_268

TTGGATTCCTGACTTAAAGACTTATCAACGCTTCAAATTTTAGCCAAAA

>Pde_miRNA_269

TTGGCATTCTGTCCACCTCCCATCTGTAGAAACTACAAGTTGTTCTACTTTCTGGAGGTGGGCATACTGCCAACT

>Pde_miRNA_270

TTGGGGTTGGTTTAGATGTGTTCTTATCCTTATTTTGAGATCATTTGGACTTCTAGAACTCGAGAT

>Pde_miRNA_271

TTTACATGGTTGTTAATTTCAAAATCCATGAAATTAGTCGAGATACATGTAAACT

>Pde_miRNA_272

TTTACCCGATAAGATAAGGACCTCCTCACCGAGGAGAACTTTTCTTAAACCGTAGATA

>Pde_miRNA_273

TTTACCCGATAAGATAAGGACCTCCTTACGAGGAGGACTTTTCTTAAATCATAGACAGA

>Pde_miRNA_274

TTTACCCGATAAGATAAGGACCTCCTTACTGAGGAGGACTTTTCTTAAACCGTACACAGA

>Pde_miRNA_275

TTTACCGACGGATAATTGAATATTAATATTTTTTAAGTATTCCGTCGGTAATTT

>Pde_miRNA_276

TTTACCGACGGATAATTGAATATTAATATTTTTTAATTATTCCATCAGTAATTCCGTCGGTAATATT

>Pde_miRNA_277

TTTACCGACGGATAATTGAATATTAATATTTTTTAATTATTCCATCGGTAATTC

>Pde_miRNA_278

TTTACCGACGGATAATTGAATATTAATATTTTTTAATTATTCCGTCGGTATTTC

>Pde_miRNA_279

TTTACCGACGGATAATTGAATATTAATATTTTTTAATTATTTCGTCGGTAATGTT

>Pde_miRNA_280

TTTACCGACGGATAATTGAATATTAATATTTTTTAATTATTTCGTTGGTAATTC

>Pde_miRNA_281

TTTACCGACGGATAATTGAATATTAATATTTTTTTAATTATTCCGTTGGTAATTC

>Pde_miRNA_283

TTTCCAAAATTATCTTCGTCGATTTTATTTCTTTTAATATTGAGTTGGTTAGAATTTAACTTTGTAATAA

>Pde_miRNA_284

TTTCCACGGCTTTCTTGAACTGTACGTATTATCAATGGCTTTTTACAAGACTGGAAGATGGTTTCCATGAAGAAGAATTGTCACAAAAACAGTTCAAGAAAGCCCTGAAAAAT

>Pde_miRNA_285

TTTCGCTGCCGCAACTGAACCTCCTGCAGCGAATATCAATTCGCTGCTGATTTGTGTGTTGCGCAGCGAAATTCTGTTTTGCTGCCACAACTGAACCTGCTGCAGCGAATTGATATTCGCTGCCGAGTTGTGTGTTGCACAGAGAAATTCTGTTTCGCTTCCGCAACTGAACCCCCTGCAGCGATTTGATATTCGCTGCCGATTTGTGTGTTGCGCAGCGAAATT

>Pde_miRNA_286

TTTCGCTGCCGCAACTGAACCTCCTGCAGCGAATTTGATATTCGCTGCCGATTTGTGTGTTGCGCAGCGAAATT

>Pde_miRNA_287

TTTCTTCACGAAACCTGTTTGCTGGTAGAATTCAGATGTCATCTTTGAAAAATCATATCTCCCTCATATGGCATCTTTTTTTGGCTGAAATATGGAGCGTTTATAGAACTT

>Pde_miRNA_288

TTTGCACGTCAGTCTGATCCTGAAATTACACTGATTATGACTATTTTTCTACCTTTTCATTCTCATTGGGATTTCTACGTGTGTTTT

>Pde_miRNA_289

TTTGCACGTCAGTCTGATCCTGAAATTACACTGATTATGACTATTTTTCTACTTTTCATTCTCATTGGGATTTCTACGTGTGTTTT

>Pde_miRNA_290

TTTGCTCATTCTTCTCAACCTTTGATGAAAGACACTCTAATGCTCCACCATGCCTTCATCCCTTTGATGAACCCTGATGTTTCACCCTATAGACCTAAAAAACATAACTTCCTATTAGATGGACAGCTGGAGCAGTAAGACACCATTGATCAAAGGTTGAGGAGAATGAGCAAGGG

>Pde_miRNA_291

TTTGTTGATAGTCATCTAGTGAACATTGAAAGTCATATTAGATGACCATCAACGAAAA

>Pde_miRNA_292

TTTTGCTCATTCTTCTCAACCTTTGATGAAAGACACTCTAATGCTCCACCATGCCTTCATCCCTTTGATGAACCCTGATGTTTCACCCTATAGACCTAAAAAACATAACTTCCTATTAGATGGACAGCTGGAGCAGTAAGACACCATTGATCAAAGGTTGAGGAGAATGAGCAAGGGG

>Pde_miRNA_293

TTTTTTAGTGGAAAAATGAAATGGAAGTCGTCACCTAGTATTTTGGTCACTAGGAACCC

>Pde_miRNA_294

aagatctgtgcactgtgaagagaaaactgaatgtgaaaaagaaagcctttttagccgaacaagtaagtgccattcttcagaacaataatgctttgaaatataaagaccctggttgtcctacaatttcttgctttattggagaacataaaattgaaagagctttacttgatcttggagctagtgtgaatttacttccatattcagtctttcagagtctcaatctagg

>Pde_miRNA_295

aagtaagtagacatattgataaagtgttgaatgcacaaactgttgaagaagttcagaaaaatcggttaagacttatgacaacaattgaaagtgttcgatggcttagcttacaagcatgtgcatttagaggt

>Pde_miRNA_296

ctctagagtcagtgaataaacaaataaaataaatttatttttacacatgcacatattttttatttttctagctaaataaaataaatataatgaataaaaataatataaatttaaatcggtatttttattcctgactctggcgtc

>Pde_miRNA_297

taggctggaaacctgtgacccgatcttttagatcattctcaggtataacaacgtcacgt

>Pde_miRNA_298

tagtattttggtcactaggaaccctaattggtctcagagatcgggtacggggactggttgccgtaaaggaagctatt

>Pde_miRNA_299

ttaccgacggataattgaatattaatattttttaattattccgtcggtaata

*Populus trichocarpa* miRNA precursor sequences

>Ptr_miRNA_1

TTCATTAAGGATGGCCTGAGTAAGGAGTCTTTAGATAATCTTCTAGTAACTCTTGAGACGCATCCTAGTGGAGA

>Ptr_miRNA_2

CTAACATCAGAATTTGAACAAGTGGTATACATAAAAGTTGTGGGAATCTGTCTCAGCTTTCCACCATAAGACAAACGAGCTCATTTGGACTTCTAGAACTCGAGAT

>Ptr_miRNA_3

TATGTTTTGAATTGTTTTGATGTGCTAATGTCAAAAATAATTTTTAAAAAATGAAAAAACATCATTGACATGCATTTTGGCACGAAAAGTTATTTAAAAAGCACC

>Ptr_miRNA_4

TTAAAACTCGAAATCGGTCAAACTCGGTTAAAATCGGTAAAATCAGACCAATTTTACGAGTTTGATTGATTTCGAGTTTTAACC

>Ptr_miRNA_5

GGAATGTTGTCTGGCTCGAGGTCACTAATGGGATCTATGATTTTATCTCAATTGATTGATTTTCTTTTAAATTCTAGTAAATTGAATTGAGAGATATCATGATCAACTTATATTTAATGATGTCGGACCAGGCTTCATTCCCC

>Ptr_miRNA_7

GGCGACATGGCCGAGTGGTAAGGCGGGGGACTGCAAATCCTTTTTTCCTAGTTCAAATCCGGGTGTCGCCTG

>Ptr_miRNA_8

TTAAAACTCGAAATCGGTCAAACTCGGTTAAAATCGGACCAATTTTACGAGTTTGACCTATTTCAAGTTTTAACC

>Ptr_miRNA_9

TTTCTTCACGAAACCTGTTTGCTGGTAGAATTCAGTTGTCATCTTTGAAAAATCATATCTCTCTCATATGACATTTTTTTTGCTGAAATTTAAAGCGTTGATAGGTCTTTGAGTTAGGAATC

>Ptr_miRNA_10

TTTGCTTCCCATCTAGTTTTCTTAAAAACTGGCAAACAAGGTCTTTTTTAGTCAGATT

>Ptr_miRNA_11

ACTTCTAGAACTCGAGATATGGGCTGAACACTAAATAGTGTCCAGGTTATAGGACAGATTCTGACTTCTTC

>Ptr_miRNA_12

TTTACCCGATAAGATAAGGACCTCCTTACCGAGGAGGACTTTTCTTGAGGCATAGAT

>Ptr_miRNA_13

AAGCTCAGGAGGGATAGCGCCATGAGCATGACAAAGTCTATGTTTGAGTTAATCTCAACAAAATCAATCCAGTCATCAGTGGCGCTATCTATCCTGAGTTCTA

>Ptr_miRNA_15

TGCTGATGTCAAAAATAATTTTTAAAAAATAAAAAAAAATCATTGATATGTATTTTGACACGAAAAGTTATTTGAAAAGCACC

>Ptr_miRNA_16

CTTCATGAAAGTTCTAGGAAATTGTCTCAGCTTTTCAACATAACATGAATTGGTTCATTTAGACTTCTAGAACTCGAGATATGGGC

>Ptr_miRNA_17

AAAGAGAGAGAGAGAGAGAGAAAGGATACCTGAGATTGGACAGCATTTCCATCCCTTGAGGCAGTTCTTCCAGTGCAGTGTAGCGGAGATCCAATTTCCTCAACTCCCTAAGCTTTGC

>Ptr_miRNA_18

AACGATTCACCGACGGATCATTTCCGTCTGTGATTCCGTCGGTAATATGATTACCGATGGAATATGTTTCTTACACCGACGGAAAAATTCCGTCGGTAAAACTGTTAA

>Ptr_miRNA_19

TGCCTGGCTCCCTGTATGCCACAAGCAAAGACCAATCTTTTGTTTTAGATTGGCTGTTGCCGGTGGCGTGCGAGGAGCCAAGCATA

>Ptr_miRNA_21

TCTTCTCTGATAATATGCTTCAATTCACCGCAACTGAGTATCAGTAATTTTTCTAGATGAAAAAGACTCTGAGCGAGGGACGGTGTGAAAATAAATGTCAGTTTGTGAAG

>Ptr_miRNA_22

ATTCATGGCACAACTGATATTGGTTTGAAGTTTGAAAGGGATGATAACCTCGGATAAAATTTAGTTGGTTATGTGGAC

>Ptr_miRNA_23

TTACCGACGGATAATTGAATATTAATATTTTTTAATTATTCCGTCAGTAATT

>Ptr_miRNA_25

TGATGATTAATTGACTGCAAAAGCAACTCTCTTCTTTTTTTAGGAGAGGAGTTGCCTTTGCAGTCAAGTAATCATCAAG

>Ptr_miRNA_27

TGTTGGCAGAGCTCAATCAAATCAAAGCACTCAATGGTTGGGTTCTTTCATCTGATTGAGCCGTGCCAATATC

>Ptr_miRNA_28

GGAGCGGCCTTGGCTCACATGTGGCGGCATCCAATGTACTTCATGTGTTCTCAGGTCGCCCCTG

>Ptr_miRNA_29

TCGTAATGCTTCATTCTCACAACGGCAAAGTGTTATTGCATCATCCTAATGATTATCATCAGCCGTCGTGGGAATGAACATTATGAGA

>Ptr_miRNA_30

TGATTCCTTCCAATTCCATTTGCTTAGTTTATTTTCATCGTCCCTTAAAGAAATTAAGAACAAGTGGCATTGGAGGTATCCCA

>Ptr_miRNA_32

TTTCTGTGGTGGTTTGGAAGCTTTACACGGCCGGAAATGGGAGATCGGATGAGAGAGAGAGAATCGCCGGCGAGAGG

>Ptr_miRNA_33

AGCTATTGTCTTCCATGGAATAGGCAGTGATGGCATTTGTTTTATTTTCCTGAGGTGGAGAAAAACAAGGAGACCATAACACTGTTATTCCATGGAAGATAATGACTCG

>Ptr_miRNA_41

AGCTATTGTCTTCCATGGAATAGGCAGTGATGGCATTTGTTTTATTTTCCTGAGGTGGAGAAAAACAAATAGACCATAACACTGTTATTCCATGGAAGATAATGACTCG

>Ptr_miRNA_43

TTTCCAAAATTATCTTCGTCGATTTGATTTTTTTTTAATATTGAATTGGTTAGAATTTAACTTTGTAATAA

>Ptr_miRNA_44

CTCAGGTTTCCATCAAAAAATGAATCGGTGCATTTGGACTTCTAGAACTCGAGAT

>Ptr_miRNA_45

TGGAGAAGCAGGGCACGTGCAAAATCCTGATGAAGTGCTTACACTTTGCACGCGCTCTTCTTCTCCAAC

>Ptr_miRNA_46

TTTCTTCACGAAACCTGTTTGCTGGCAAAATTCAGATGTCATCTTTGATAAATCATATCTCCCTCATATGAAATCTTTTGTGGCTGAAATTTGGAGTGTTTATAGGTCTTTGAGTTAGG

>Ptr_miRNA_47

CCGGACGGGATCTCAGGTTAGCCCAACCACACACAAGATTTTGGTTCTGGTTAAAGGGGATCGCCAGATGGGA

>Ptr_miRNA_48

GGATTGCTGTCTGGTTCGATGTCATTCATGTGAAGCTTTAACATTAATGTAGTATTGAGTGATTTCGGACCAGGCTTCATTCCCC

>Ptr_miRNA_49

CCCTATGAGGAAGCAATCCCAAAAGATAGGAAGTGCCGTATGACACTTTAATCAGCAGCGGTCACACTCATGTACCCACGTTTCCCATCCACCAATCACAGCCCAAGAGAGGCGTTTCCTCTCCCGGATTCGTCTTCCTTGGGTCGCTGATTGATCGGGATCACGTGGGCTACTGTGTGCCGCTGTGTGTGTGATTTGGAACGGGTCTCTGAGGGTTTTATTTCTTAAATCATA

>Ptr_miRNA_50

TTGCGGTTGCTTTTGAAAGTGTTTTTGTCGTGCCGAAATGTATCAAAATGATATATTTTTTATTTTTTAAAAATTATTTTTGAGATCAGCGCATC

>Ptr_miRNA_51

TCTTCACAGTGGTAAGGTTATTGAAACACCCATTCTTGAACCTTATGAGAAAGATGATGAGTCAATATCTGAGGGTAAGAAAGGGGTTAAACCTGAACATTGCAAAGAAA

>Ptr_miRNA_52

TCATGCGATGAAGAAAAAAACTGCAACAAAATGATCGATTAATTTCGTGATTACTTGTATTTAAGTCTCTGATGATT

>Ptr_miRNA_53

TGAAGCTGCCAGCATGATCTATCTTTGGTTAGAGAAAGAAAGGACTAACCCTAGCTAGGTCATGCTGTGACAGCCTCACT

>Ptr_miRNA_55

TGAAGCTGCCAGCATGATCTAACTTCCTTGCTTCTTTATCAAGGATGGATTTAGATCATGTGGTGGTTTCACC

>Ptr_miRNA_56

TTGGCATTCTGTCCACCTCCATCTGTAGAAACTACAAGTTGTTCTACTTTCTGGAGGTGGGCATACTGCCAACT

>Ptr_miRNA_57

TGTTGGCCCGGTTCACTCAGAGAAAAGACACCATTTTTTTTCAAGAAGATCATCAAAGCTTGAAAGAATAATGGTGGTCGGTCGTCTCTGATTGAGCCGTGCCAATATC

>Ptr_miRNA_58

TGTCAAAAATGATTTTTAAAAAATGAAAAAACATCATTGGTATGTATTTTGACACGAAAAGTTATTTGAAAAGCACC

>Ptr_miRNA_59

ACTCCCCCTCAAGGGCTTCCTGTTTGCCCAATCTGGTAATGATGGTTTTGTGTTTTAAGACAAAACATATACTACCAAGTATCGAGGCAGGCTGGGGGCTCTTTTGGGGGTTTAA

>Ptr_miRNA_60

CGGGGAACAGGCAGAGCATGGATGGAGCTACTAACAGAAGTACTTGTTTTGGCTCTACCCATGCACTGCCTCTTCCCTGGC

>Ptr_miRNA_61

GGTTTGTGCATGAATCTAATATAGCTAAAAAAATCCCATCAAACACAAATTAAAATACTAGATAGCAATCAGCACTAGTTTAGTATAGTATTAGATTCACGCACAAACTCG

>Ptr_miRNA_62

ACTTCTAGAACTCGAGATATGGGCTGAACACTAAACAGTGTCTGGGCTACATGATAGATTCTGACTTCT

>Ptr_miRNA_63

TGGAGAAGCAGGGCACATGCTAAATCTATCAGCTTGAAAGTCTGATAGTTTTGCATGTGCTCTATCTCTCCAGC

>Ptr_miRNA_65

GCTCAAGCACAAATTCGATCTTGCTTAACAAGGTACCGGCAAAGTCAGATCGAATTTGGGCTTGAGATT

>Ptr_miRNA_66

TTCCACAGCTTTCTTGAACTTCCTAGAGCCTAGAGGTGCTGCTAGCTATACATATAACTTAAGAAGTTCAAGAAAGCCGTGGAATA

>Ptr_miRNA_67

TTGGTTAATTCTGCGACTCGGATCATAAGACGGATATAATTCTATAAAAAAATTTAAAATAAATTATGAAAACCAATTTATACAGAGTCATGCCGGATTAACCTGTT

>Ptr_miRNA_68

TGGCTTCTCTTCTTTGGCAGGTGATGGTATAGATTACAACTTATAGCTTTAATCTAGTATTCAGTTACCCGCCAAAGGAGAGTTGCCCTC

>Ptr_miRNA_69

TGGTTTTCGGAAAACCACTTTTCAGACTTTTATATGTTTGTTTGCTATTAAAAAAGTTGGTCAACGGAAAACACT

>Ptr_miRNA_70

CATTTTCAACGTCATCTCAGAAGAATTTAAATTTTTAAAGGTATTTTGAACGCGTTCAACTTTTAACGCGTTAATTTTACATTCACTGATTTTCCTTGTTGAGTCGACGTTGATATTGCT

>Ptr_miRNA_71

TTAACCGAGAAAACCGAACCGAGATAAAAAACCTATTAAATCGATTACCAAAACCAAAACTCTTTCCGGTTCGGTTCGGTTTCGG

>Ptr_miRNA_72

AAATCGGTCAAACTCGGTCAAACTCGGTTAAAATTGGTAAAATCGGACCGATTTTACGAGTTTAACCGAGTTTGACTGATTTTG

>Ptr_miRNA_74

TAATTGATTATTGATGTAGTGGCCACCTATTGATTTAGTGGACTTGGTGGACAGCAGCACTCAAGCATTTTGGCT

>Ptr_miRNA_75

TGACGCATGGATTCGACAGCCCAAACAGCTCCTAGGGACTCATAAAACATTAACGAACTGCTGTGATGGGTGACACATGGTTTCGACATCCCAAACAGCTCCTAGGGACTCAAAAATAATCAACGAACTGCTGTAACGGGTGACGCATGGATTCGAGAGCC

>Ptr_miRNA_76

AGGGACTCAGAAATCATCAACGAACTTCTGTAACGGGTGACGAATGGGTTCAACGGCCCAAACAGCCCCTTGGGACTCATAAAACACTAACGAACTGCTGTTACGGGTGACGCATGGTTTCGACAGCCCAAA

>Ptr_miRNA_77

ATTTGGACTTCTAGAACTCGAGATATAGGTTGAACACTGAATAGTGTCCGGGTTGTAGGGCAAATTC

>Ptr_miRNA_78

GCTGCATCACCAAGATTCACATGCAAATGCATGGCCGATGATATATGTTAGAAATTTTTTTTATATAAAAAAGATATCTTTCTTTCTTTCTGATCATCTGCCAAAGTTCTTTGGAAGTGAGAATCTTGATGATGCTGCAT

>Ptr_miRNA_79

TCGCTTGGTGCAGGTCGGGAACTGATTCGGCGATTTGATTGCCAGATGGCTAAACACGATTGGCTGTGAGGCAAATTATAAAAAGAAAGAGAATTGGATCCCGCCTTGCATCAACTGAAT

>Ptr_miRNA_80

GAAAGCTTCACCGGCGGGGCATTTCCGTCGGTGATTTTGTCGGTAAATTAATTACCGACAGAATATGTCTTACACCAACGGAAAAATTCCGTCGGTAAAACTGTTAA

>Ptr_miRNA_81

ATTTGGACTTCTAGAACTCGAGATATGGGTTGAACACTGAATAGTATTTGGGTTGCAGGACAGATTC

>Ptr_miRNA_84

TTGAATCATGAAAGTTTTAGGAAATTGTCTCAGCTTTCCAGAAAAAAAAAGTTGAGGTCATTTAGACTTCTAGAACTCGAGATATGGGC

>Ptr_miRNA_85

AAACGGAAATACCGAGAGAATATTTTCGTGGGAAAAAAAATTACTGACGGACAAAAAATTACCGACGAAAGATTCACCAACGGAGAATTTCCGTTGGTGATTCCGTCGGTAAATTAATTACCGACAGAATATATGTCTTACGCTGATAGAAAAATTCCATCGGTAAAACTGTTAAA

>Ptr_miRNA_86

TATTGGTACGGTTCAATCAGAAAGTAATGCTCCCAAAAGTATAGAGTACTATTGTTTGATTGAGCCGTGCCAATAT

>Ptr_miRNA_88

GGATTCCAAACTGACTCAAAATATTATCTTAAAAAACTTGATCACTATTTTGTGATCATTGTTTCGACTTGGAATTGCCT

>Ptr_miRNA_89

AAAGTGGGCACCTCGAACTGCCAAGTTTGCAGACTGCCCGAGGTGCACGATTTGG

>Ptr_miRNA_90

TATATATATTTTGTGGGGACCCAAAAATGGGTTACAACAACATAAATAGG

>Ptr_miRNA_91

GCTATGATGAGTATGAAAAGGTTATGAGAGTTTTGATGAAATTCATTCCTGAGCGGTTATCTGAGCAA

>Ptr_miRNA_92

GGATTCCAAACCGACTCAAAATATTTTCTTTCAAAAACTTGATCACTATTTTATGATCATTGTTTCGACTTGGAATTGCCT

>Ptr_miRNA_93

TGAAGCTGCCAGCATGATCTAAATTAACCTCCTTCTTTATCAAGGATGGATTAGATCATGTGGTAGTTTCACC

>Ptr_miRNA_94

TGGTTTGATCTTGCCGCAATGATGGCATCATTGTATTAGCTCTATCTGATCATT

>Ptr_miRNA_95

TCAAGTGATGAATTTGGATTACAAATTTCTCACGACGGTCGTCTGAGACACATCGTGTCAATTTTGATGTAATGATCCAATGTTTTCTGATGATT

>Ptr_miRNA_96

GACAAAAATGGCATAAGAGAGGTACAGTGGGAGGCAGGAGAAAGACCAATATCTGCCACCCTTCTTTCTCTCTTATGCGTTTTTGTCTC

>Ptr_miRNA_100

GACAAAAATGGCATAAGAGAGGTACAGTGGGAGGCAGGAGAAAGACCAATATCTCTGCTACCCTTCTTTTTCTCTTATACGTTTTTGTCTC

>Ptr_miRNA_102

TTGGCATTCTGTCCACCTCCTATCTTTAGAAATTAGAATATCTCTTTCATATGGAGGTGGGCATACTGCCAACC

>Ptr_miRNA_103

GGATTGTCGTCTGGTTCGATGTCATTCATGAGAAGCTCAAACATAAACGTAATATTGAATGATTTCGGACCAGGCTTCATTCCCC

>Ptr_miRNA_105

GGTGGGTGAGCGGGGAAGATAACTTTGGTTTTTGAGATAGTACTTGTTATTTTCCCTACTCCACCCATCCC

>Ptr_miRNA_106

AGTAGGATTTTCGGAAGGTTATCGGGTGGGTGAGCGGGGAAGATAACTTTGGTTTTTGAGATAGTACTTGTTATTTTCCCTACTCCACCCATCCCATAGGTTTCCGATCATTCCTCC

>Ptr_miRNA_107

AATCACTACTTCTGAATTGGGACCTAATAGGGATACTATAAGAGTCCTGACGATTCTTCCACTGATTCT

>Ptr_miRNA_108

AGGGATATAACTCAGCGGTAGAGTGTCACCTTGTATTAGAAGGAGATCGATTTATGACGTAGCATTTGCAGAATTTGATCGCAATCCTAATTTTATTTCGAATGATATTTTACCTGATGTTGTTGCATGCT

>Ptr_miRNA_109

TGAAGCTGCCAGCATGATCTGAACTTTCCTTAATTTTCCTATACGGGAAAGACTAGATCATGTGGTAGTTTCATC

>Ptr_miRNA_110

ACTTCTAGAACTCGAGATATGGGCTGAACACTGAATAGTGTCTGGGCTGCAGGACAGATTC

>Ptr_miRNA_112

TCGGACCAGGCTTCATTCCCCTCAACCAATAAACTTATGTATGCATTTTATGCGCAAATTCAGTTCTTCTTTTGGATCTTTCTTTCTTTTTTTCACAGAAGAGATGAAGCATGAGTTCATCTCTGAGCGTAAGGTTGAGAGGAACGCTGTCTGGGTCGAGG

>Ptr_miRNA_113

GGAATGTTGGCTGGCTCGAAGCTTAAGCAAAGAGTTTTCTCTCAAGAAACAACTGTTAAGGCTTCGGACCAGGCTTCATTCCCC

>Ptr_miRNA_116

GTGCTTATCGACGGTCTTGTGCTGGCTAGTTGTCTTCCACCAGCACACAACATATTCGTAAGCTACAT

>Ptr_miRNA_117

TTTACCGACGGATAATTGAATATTAATATTTTTTAATTATTCCATCGGTAATTC

>Ptr_miRNA_118

ATTTGGACTTCTAGAACTCGAGATATTGGTTGAACACTAAACAGTGTCTGGCTGGAGGACAGATTC

>Ptr_miRNA_120

CTCAGAATAGCTAGGATTTCCAACAGCCCAGAATCGGCGCATTTGGACTTCTAGAACTCGAGAT

>Ptr_miRNA_122

TGCCTGGCTCCCTGAATGCCATCTAGGAAGCTTGTCAAAGAGTGTTGGCAACCTTTCTATTTGGCATGAGGGGAGTCGAGCAGG

>Ptr_miRNA_124

GCAGTGATGTCATTGATAATCCCAGCTCCATGAGATCTTCAATTTGAAGGTCTAGGAATAGATGTTATCAATTTCTGTCTGAGCCT

>Ptr_miRNA_125

TTATCAATTTCTGTCTGAGCCTATAATGTTGTTCAAGGAAGATATGCAATATTTTGTTTTTTGTTCACACATAATTTAGTAATA

>Ptr_miRNA_126

AGGTTCCTTCCAGCCTCAAGCATCTTTAAGATGGTGGCGATGTCTATTTTGGGCTGAAGGGAGCTCCC

>Ptr_miRNA_127

TGACAGAAGAGAGTGAGCACACAGAGGCATATTTGTATAAATCTATACCATTGCTTTTGCGTGCTCACTTCTCATTCTGTCAGC

>Ptr_miRNA_128

GACTTGAAATCTGTTGGGCTTCGCCCGTGCAGGTTCGAACCCTGCTG

>Ptr_miRNA_129

TTTCCACGGCTTTCTTGAACTGTATATATTATCAATGGCTTTTTACAAGACTGGAAGATGGTTTCCATGGAGAAGAATTGTCACAAAAACAGTTCAAGAAAGCCCTGAAAAAT

>Ptr_miRNA_130

TGACAGAAGAGAGTGAGCACACAGGGTACTTTCTTGCATGACATCCATGCTTGAAACTTTGCGTGCTCACTCTCTATCTGTCACC

>Ptr_miRNA_131

TGACAGAAGAGAGTGAGCACACACGAAAGTATATGGTATGAAGGCATTCCATTGCAGGGTGTGTGCTCACATCTCTTCTGTCAGC

>Ptr_miRNA_133

TTCCACAGCTTTCTTGAACTGCACCTATTTGAATATTGTTGTTGATGTTGCCGTGCATGTACATATGACATTGTATTTTTGTTGCGGTTCAATAAAGCTGTGGGAAG

>Ptr_miRNA_134

ATTTGGACTTCTAGAACTCGAGATATTGGTTGAATACTAAACAATGTCTAGGCTGGAGGACAGATTC

>Ptr_miRNA_135

AACTTGATGTGAAAACTGCCTTTCTCCATGGTGATTTAGATGAAGAAATCTACATGGAGCAGCCTGAAGGGTTTGAAGCAAAAGGTAAA

>Ptr_miRNA_136

GTTTGTTCTCTCTGTGATCTCAAGTTCGAGCTCTGTGGTTGCTAATATGATGATCATTGTAGGTTTACATGGTTGTTAATTTTAGAGCTCGTGAGATTAATCGAGGTACGCACAAACTG

>Ptr_miRNA_137

TAATCTGCATCCTGAGGTTTGGATCACCACATGTTTTGATCTAGTCCTTGGGTTGCAGATTACC

>Ptr_miRNA_138

ATCACGAGCCATCATAACTGTAGGAGTCATCGTGATCATCCCTAACAAAACATGATGATCATGATGATTCCTACAGTTATGATGGCCCATGATCT

>Ptr_miRNA_140

CGATGTTGGTGAGGTTCAATCCGAAGACGGATTTACACGTGAAAGTAATTGTAAAATACGATCTCAGATTGAGCCGCGCCAATATCACT

>Ptr_miRNA_142

TTCCACAGCTTTCTTGAACTTCCTTGCCATGCTTAACTTGTGTGTGTGTATAGATCACTATATATCTGTATAGTTATACACATTTAGCTAGCTAGCACCATTGAAGCTCAAGAAAGCCGTGGGAGA

>Ptr_miRNA_143

AACGGACCGAGATTTAAAATGAGATGAAAATTATTTCGTTTTGTTCTGTTTT

>Ptr_miRNA_144

AAGCTCAGGAGGGATAGCGCCCTAAGGATAATCATGGGCTCTTTTTATGTGGTTTTTGATTCTCAGTGGCGCTATCCATCCTGAGTTTCA

>Ptr_miRNA_146

GTTTATTGAACATAACTAATTTTATTACACTTTCATTAAGTTGATTTGCATGTAATCCCGAGAGGTAGCTCAATTAGTCAGATATTAGATTTGTTTTTTAATGATTACGAGTTCGAGCCCTCTCAGGGTCATTAGAGGCTTACATGGCAGGACCCATGAAATTAGTCGAGATGCACATAAACTG

>Ptr_miRNA_147

TAGTATTTTGGTCACTAGGAACCCTAACTAGTCAACAAAGATTATATGGTACGGGACTGGTTACGCAAATGGA

>Ptr_miRNA_148

GGAATGTTGGCTGGCTCGAAGCTTAAGCAAAGAGTTTCCTAACATGAAACAACTGTTAAGGCTTCGGACCAGGCTTCATTCCCC

>Ptr_miRNA_150

TTTGTTGATAGTCATCTAGTGAACATTGAAAGTCATATTAGATGACCATCAACGAAAA

>Ptr_miRNA_151

CTAGTTCACATGTTGCTATCAACTGACCGGTTAGTCTAAACTGGCCGACCGATTGGTTTGTGTAGCGATTGATTAGTT

>Ptr_miRNA_152

GCCTATGATGATTAAATTTGCCTACATTGTCTGATCATCTAATGAATGACAGAGACATATAATTAGTTCTGAGGCTT

>Ptr_miRNA_154

ACTTCTAGAACTCGAGATATGGGCTAAATACTGAACAATGTCTGGGCTGCAGGACAGATTC

>Ptr_miRNA_155

TTTGAACAGGTAGTCTTCATGACAGTTCTGGGAATTTGTCTCAGATTTCCAACAAAATAAGAATGAGCTCATTTGGACTTCTAGAACTCGAGAT

>Ptr_miRNA_156

ATTTGGACTTCTAGAACTCGAGATATGGACTGAATACTATGTTCTCGACAATTATGTTGTCTAGATTA

>Ptr_miRNA_157

AGGGATGTAGCGCAGCTTGGTAGCGTGTTTGTTTTGGGTACAAAATGTCACGGGTTCAAATCCTGTCATCCCTAC

>Ptr_miRNA_159

ACAGATTATTCAGAATTGTAGCCTCCTTTTTGTATTTGATCTCAATTACTTTAATAGTGAGGTTTACCAAGAATGGAAGTTGACAA

>Ptr_miRNA_160

TATTCCGAGATTCTGAACAGAATAAAAGATGAATTATGGGCAGAATTCTTGTATAGT

>Ptr_miRNA_161

TGTGCTCGTAAAGTCGCACTAACCTCAGGGTCACTGGAAGGTTATATGGTCATTAACTTCAGGGTCCATGTGATTAGTCGAGATGCGCGCAAGCT

>Ptr_miRNA_162

TTCATTAAGGATGGCCTGAGTAAGAAGTTTTTAGATAATCTTCTATTAACTCTTGAGACGCATCCTAGTGGAGA

>Ptr_miRNA_163

CAGAATTTGAGCACATAGTCCTTATGAAAGTTGTGGGCAATTGTCTCAGCTTGTCAACAAAAAAAAAAAAAAAAACCAGAGCTCATTTGGACTTCTAGAACTCGAGAT

>Ptr_miRNA_164

TCGCAAGTCGGAGGCCTGGCTGACGCGGCTGGTGCGGACCGCCGAGCTGGGGGATTGCGAGGAGAGCTATACGCTGGCGTGGGCGTGGCATTAAATTGCCGTGCGCGTGCCCATGCGTTGGCTCTCCTTGCAATCCCCGACCTCGTGGCGTGATGTGCCCGCTGCCGAGGCCTGGCCTCCGTCTTCCAAGC

>Ptr_miRNA_165

GGAGCACCATCAAGATTCACAAACTTTATTAGGGCTAATAAGTGGTGATGATGGTGGCTTTTGGTGGTCCCTTCGTTTCAACCCAATAGCCATTTGAATTGGGAATCTTGATGATGCTGCAG

>Ptr_miRNA_167

ACCAGTTCCTTGATGATTTCGGAGTCCCTAGGGGTTGTTTGGGCTGTCGAATCCGTGCGTCACCCGTGACACCACTTCCTTGATGATTTCGGAGTCCCTAGGGGCTGTTTGGGCT

>Ptr_miRNA_168

TGTGAGGACCATGATATGAGTCCAAGATCAGATCGTCTCATGATGACAGGGGGAGACACCTCAGGAGAAGGTGTGAGGACCATGATATAAGTCCAAGATCAGACT

>Ptr_miRNA_169

AATGCTGTCTGGTTCGAGACCATTCACCTGAAGAGCACGCATTCATCTTTTGAGTGATCTCGGACCAGGCTTCATTCC

>Ptr_miRNA_170

AATGTTGACCGAATATGGATGAAAAGTTGTTTTCTGTTTCCTTTCCTCCACATTCGGTCAATGTTCC

>Ptr_miRNA_171

AATGGCCATTGTAAGAGTAGAAGGATCCATGAAGCAAAACATTGACTCCTGATATTCCACTCTTACAGAGTCCATTGA

>Ptr_miRNA_172

GGTCCGAGGGAAAGTTTTGTTGAACAAGTCCCGGTACATGGATCAGACACTTGCTCAACAAATCTCTCCCTCTCACCCC

>Ptr_miRNA_174

TGCCTGGCTCCCTGTATGCCATTTGCAGAGCCCAACGGATCCTCGATGGCCTCCGTGGATGGCGTATGAGGAGCCATGCATA

>Ptr_miRNA_176

GGTTTGTGCGTGGATCTGAGGCCATCACAACCGTCCACTACACGACCACCCAATGGCTTTAGATTCACGCACAAACTCG

>Ptr_miRNA_178

TATGGGAGGATTGGACAGTACTGCTTGGTTTTAATTAACCAAAGTCTGTTCTTTCCAACGCCTCCCATACC

>Ptr_miRNA_179

TGAAGCTGCCAGCATGATCTTAACCTCCCTCCTTTGTCGAGGAAAGAACAGATCATGTGGCAGTTTCACC

>Ptr_miRNA_180

GGTTGTTTACTTCAAGATCCGGAAGATTAGTCGAGGTACGCGCAAGCTG

>Ptr_miRNA_181

CGGTTCGGTTCGGTTTCGGTTTTTTAAGCCTGGAACCGGAAAAACCAAACAAAAAAAAAAACTGGAAAAAACCGAGCCAAACCGAGCCAAAACCGAGCCAAACCGAA

>Ptr_miRNA_182

GGAGCGACCTGAAATCACATGTGGGCTGCACCCTCCTGGGTTATCTTGAGCAACATGTGTTCTCAGGTCGCCCCTG

>Ptr_miRNA_183

GGAGCATCATCAAGATTCACATGCAAATGCACGGCCGGTGATGTTAAGAGTTAAATCTTTCTTTGTTTCTGTTCATCTGCCAAAGTTCTTTGGAAGTGAGAATCTTGATGATGCTGCAT

>Ptr_miRNA_185

TGCATTTGCACCTGCACCTTACTTGTTTCTTTCTTTTGTTTTTGACTCCAAAACCAAAAGTAAGTTTGAAACATTAAAACAAGCTTGAGGTGCAGGTGCAGGTGCAGG

>Ptr_miRNA_186

CCGACCTTAGCTCAGTTGGTGGAGCGGAGGGCTGTAGGTCGCTG

>Ptr_miRNA_187

GCGGCAGCATCAAGATTCACAAACTTTAAGGCTTGAGTTGGGGTGGTACACGGTCCCCTCCTTTACTCGAAAGGTTCCTTAATTTCTGATGGGAATCTTGATGATGCTGCAT

>Ptr_miRNA_188

AAGCTCAGGAGGGATAGCGCCATGAGCTGATGATAAGTTGATGTTTGATGGGTTAATCTCAACATAATCAATCTAGTCATTAGTGGCGCTATCTATCCTGAGTTCTA

>Ptr_miRNA_190

AATGCTGTCTGATTCGAGACCATTCACTTTAAGCACACATTCATCTTTCGAATGATCTCGGACCAGGCTTCATTCC

>Ptr_miRNA_191

TGAACAATGGCCATTGTAAGAGTAAGTGGTCCATGAGTCAATAACTCTCTTTGCAGAAATGCATGACAAAGCATTGACTCCTATTTCACCCTTACAATGTCCATTGATTAAG

>Ptr_miRNA_193

TTTCGGTTCGGTTCGGTTTTTATCAAAAAAAATAATCAAACTGAATTTTTTTAAAAAAAAAACTGAAACCGAACCGAAACC

>Ptr_miRNA_194

TTCGGTTCGGTTCGGTTTTTATCAAAAAAAATAATCAAACTGAATTTTTTTAAAAAAAAAACTGAAACCGAACCGAAAC

>Ptr_miRNA_195

GGAGTGGCTCCAGAGAACACAGGGGGTTGGTTTTCTAGCTGTAAGCTACAAGATGGACAAAGCACTCTGTGTTCTCAGGTCACCCCTT

>Ptr_miRNA_196

CCATGGGTTTGCTCGTGCCGACTTGGTCTTTATTACTCGGGTTACAAGTTTTTCATGCCAACTCGGATAGACTTAGGCTCGGTC

>Ptr_miRNA_197

CATTTTCAATGTCGTCTCAAAAGAATTTAAATTTTCAAAGGTATTTTAAACACGTTTAACTTTTAACGCGTTATTTTTACAATCATTGATTTTAATCGTTGAGTCGACGTTGATATTGCT

>Ptr_miRNA_198

TGCCTGGCTCCCTGTATGCCATTTGCAGAGCCCACCGGTTCTTCGATGGCCTCCGTGGATGGCGTATGAGGAGCCATGCATA

>Ptr_miRNA_200

AGAATCTTGATGATGCTGCATCAGCCATAAATGACTGTATAAACACACCTGAAATAGTTGGATCTAAGATTTTCT

>Ptr_miRNA_201

GCAGCATCATCAAGATTCACATTCAAACAGATGTACGGCAGCTAGCTAGCTAGATAGATAGACAGCAATGTATTTCTTTGAAGGTGAGAATCTTGATGATGCTGCAT

>Ptr_miRNA_202

GGAGCATCATCAAGATTCACAAGCTTTATTAGGGCTAGTGTGTGGTGATGATGGTGGCTTTTGGTGGTCCCTTTTTTTCAATCCAATAGCCCTTTGAATTGGGAATCTTGATGATGCTGCAG

>Ptr_miRNA_204

AGTTTGTTCGTGGATCTGACGCCATCACAACCGTTCATTTCACGACCATCCAATGGCGTTAGATTCACGCACAAACTCG

>Ptr_miRNA_206

TGAAGCTGCCAGCATGATCTTAGCCTTCCTCCTTTGTTGAGGAAAGAAACAGATCATGTGGCAGTTTCACC

>Ptr_miRNA_208

CATGAAAGTTCTAGGGAATTGTCTCATCTTTCCAGGGTAAAAATTTGAGATCATTTGGACTTCTAGAACTCGAGAT

>Ptr_miRNA_209

GTCTCAGCTTTCATTGTTTTTGTTTAATTACTGTGCTGCCAGCCAATGATGCAAACCCTGAGTTTCACTGAAAGCAGGGCTATCGATCTGCCTAAACTGTTGTTAATTCATAAACTAGGACGGTCTGAGGCTT

>Ptr_miRNA_210

TGAAAGTTCTAGGAAATTGTCTCAGCTTTCCAACAAAAAAAGAATCGGTGCATTTGGACTTCTAGAACTCGAGAT

>Ptr_miRNA_211

CAATATCAAAATTTGTTGAGTCGACGTTGAACCCACTCAAGTCATTGTTGAGTCGACGTTGATATTGCT

>Ptr_miRNA_213

CAATATCAAAATTTTTTGAGTCGACGTTGAACCCACTCAAGTCATTGTTGAGTCGACGTTGATATTGCT

>Ptr_miRNA_215

TTAACCGAGAAAACCGAACCGAGATGAAAAACCGATTAAACCGATTACTAAAACCAAAAATCTTTCCGGTTCGGTTCGGTTTCGG

>Ptr_miRNA_216

CAATATCAAAATTTGTTGAGACGACGTTGAACCCACTCAAGTCATTGTTGAGTCGACGTTGATATTGCT

>Ptr_miRNA_219

ATAATATTCCTGACTCTGGCGCCAGTGAAAATTCCACCACGAATAATTCCAACTCTGACGTTGGTAAAAATTCCCCCAGGAATAATTCCAACTCTGGCGTTGGTAAAAATTCGTAGCTATAA

>Ptr_miRNA_220

TGCCTGGCTCCCTGTATGCCACAGCAAAGACCAATCTCTTCTGTCTTAGATTGGCTGCTGCCGGTGGCGTGCGAGGAGCCAAGCATA

>Ptr_miRNA_221

GCTTGCATGGTCGTTAATTTTAGGATCCGTAAGATTAGTCGAGGTGCGCGCAAGCTG

>Ptr_miRNA_223

GGTCTTGAGTTTGTTCCTCAATGATCACAAGTTCGAGTTCCTTGAAAGCCGTTGGAGACTTACATGATTTTTAACTTCAGGGTCTGTTGGATTAGTCGAGGTGCGCGCAAGCTG

>Ptr_miRNA_225

TTTTCACCGGAAGTAAGCCTCTTTTCCTTCGTTGATCCTTCTGTTAAATCCATTTCTAATCACTTTCTTGGAATAGATTTAATAGAAAGGATGATCAAAGGAAAAGAGGCTTACCTCCAGGTGAAAACA

>Ptr_miRNA_226

TGGAAAATACCGACGAAAAATTTTGTCGGTGATTCCCTTTGTAATTGACATGATGAACAGTGTTCACAGTTTACCGATGGAATTACCGACGGAATTTTCCGTCGGTATTTTCCAGA

>Ptr_miRNA_227

TATTGGTGCGGTTCAATCAGATAGTAATGCTCCATAAGCATAGAGCTCTATTGTTTGATTGAGCCGTGCCAATATC

>Ptr_miRNA_228

TCATTGAGTGCAGCGTTGATGAAATCCTCCATTTTGTGCTATTAAACTGTTACCAACCCTTTATGGGGCATGGCATCATTTCACCAGCGCTGCATTCAATCATG

>Ptr_miRNA_230

AATTATTTTTGAGATCAGCGCATCAAAACGATTCAAAATATACAAAAAGAATTAATTTTTAGCAAAAAGAATTAAATTTTGGTGGAACGCGGGCCCTCTAAAGCTAAACAATTTTGGTTTATGTGTTAAAAGCATTTTAAAAAAAGTTTAATTTTTTATATTTTTATATTATTTTGATTTATTAATATCAAAAATAAATTT

>Ptr_miRNA_231

TGCTATCAGAAACTAAGAACCTGAGGTGTTGGGAGTTTTTAACCGCTTTAGACTTGTTGGGATTTGGTTTTGAGCAAT

>Ptr_miRNA_232

AGTTCCTTTTTATTGACATTAGAACGATTAGGTTTTTACCCGATAAGATAAGGACCTC

>Ptr_miRNA_233

TTTACCGACGGATAATTGAATATTAATATTTTTTAAATATTCCGTCGGTAATTC

>Ptr_miRNA_234

TGTTGGGATGGCTCAATCATGTCAAATCTCCCAAATTATGATGTTGGGTCTTTTAATCTGATTGAGCCGTGCCAATATC

>Ptr_miRNA_235

TTCTGAACTCTCTCCCTCAACTGCTATTCTAGAGCTTCCTGAGCTAACCCTTGTGTTTCTGTGTAGCAGTCAAGGTGGGAGGCTCAGAAAA

>Ptr_miRNA_236

TCCAAAGGGATCGCATTGATCTAATGACTTTTGATGTCTATATGATGTTAATGTTTAGTCATTTCATTGGATCATGCGATCCCTTAGGAAT

>Ptr_miRNA_238

TTCTGAACTCTCTCCCTCAACCGCCACTCTAGAGGTTGGCACCTTGTCAATTGTTTCTGTGTAGCGGTCGAGGTGAGGGGTTCAGAACA

>Ptr_miRNA_239

TTGACAGAAGATAGAGAGCACAGATGATGTTTTGCAGTAGACTCTCTGGATCTCACTCCTTTGTGCTCTCTATGCTTCTGCCATC

>Ptr_miRNA_241

TACCGACGGACTGTATCCGTTGGCATTGGACACCGACGAATTTACAGACAGAATTAGTCCGTCGGCATTTCATAGTAGCTGCCACAATCACTGACGAATATACAAACGGATATCTTCGGTCGGTATT

>Ptr_miRNA_243

GTGGTATTGATCCGGTTCGTCTTCTGTATTTGCATCAGAAGTTCTTCGCTGAAGACGAGCCGAATCAATATCACTC

>Ptr_miRNA_245

TTGACAGAAGATAGAGAGCACAGATGATGTTTTGCAGTAGACTCTGGATCTCACTCCTTTGTGCTCTCTATGCTTCTGCCATC

>Ptr_miRNA_247

AGCTCCTTGAAGTCCAATAGAGGTTCTTGCTGGGTAGATTAAGCTGCTAAGCTATGGATCCACAGTCCTATCTATCAACTGAAGGATAGGTTTGCGGCTTGCATATCTCAGGAGCTTTATTGCCTAATGTTAGATCCCTTTTTGGATTGAAGGGAGCTCT

>Ptr_miRNA_248

GGAGGCAGCGGTTCATCGATCTCTTCCTGGCCAATTTTTTGTTTAGCACGAAAAACATGAACCGATCGATAAACCTCTGCATCCAG

>Ptr_miRNA_250

GGATTCCAAACCGACTCAAAATATTTTCTTTCAAAAACTTGATCACTGTTTTATGATCATTGTTTCGACTTGGAATTGCCT

>Ptr_miRNA_251

TCAGGGCACTGCAATTCTAAATGTCTGTTGGCAAGTGGCGATGCCAGACGTTTGAAATTTACTTATCATTTATTAGTCAGAATTGCAGTGCCTTGATT

>Ptr_miRNA_253

ACGTACTAATTTTGATTTTTGAAGTTTTCCCAGGCATGGAGGTTGTGGAAAGAGGAAAAGGTACTGTA

>Ptr_miRNA_254

TATGTGAGTAATCAAGATTTTTGTAGCCACTCTTTGTTTGTCTCTGCTACGTACTAATTTTGATTTTTGAAG

>Ptr_miRNA_255

TAAAAGTTCTAGGGAATTGTCTCATCTTTCCAGGGTAAACATTTGAGATCATTTGGACTTCTAGAACTCGAGAT

>Ptr_miRNA_256

TGGAGAAGCAGGGCACGTGCAAGCTCTCTCCTCAGGCTTTCCTTGCACGTGCTCCCCTTCTCCAAC

>Ptr_miRNA_257

GAGCCCGATGGAGCTGTTTGTGGAGACGCACGTGCGGAGTCAAGACCGCCAGAAGGAGGCGCAACAGTTCGTTGATAACCGT

>Ptr_miRNA_258

GATGGGTGAGTGGGGAAGATAACTAAGCTCTGTTTGTTATTTTCCCAACTCCACCCATCCC

>Ptr_miRNA_259

TTAACCGAGAAAACCGAACCGAGATAGAAAACCGATTAAACCGATTTTAAAACCATAAAATCTTGCCGGTTCGGTTCGGTTTCGG

>Ptr_miRNA_260

AGTTGAGAAAAATTTACGGGATTGTGCCATTTTCACCGACGGACTCACCAACGGACAATAACCCGTCGGTATTTCACAGAGAG

>Ptr_miRNA_261

TAGTATTTTGGTCACTAGGAACCCTAACTAGTCTTAGAGATCAGGCTCGG

>Ptr_miRNA_262

CTTCATGAAAGTTCTATGAAATTGTCTCAGCTTTGCAACCAAAACAGAATCGGTGCATTTGAACTTCTAGAACTCGAGATATGGGC

>Ptr_miRNA_263

TTTTCAAGAAAACAAATGGAGGTCATTTAGACTTCTAGAACTCGAGATATGGGC

>Ptr_miRNA_264

AGCGCGAAGAAAGGGCTCACAGAGCTCACCAGCAGAATAATCAAGAGGAGAAACTTTAGGGGAAGGAGAGCATCTCTCTAAATTCTCTCTCTATCTGGCTGACTGAGAGCTCTTTCTTGATTC

>Ptr_miRNA_267

GCTATGATGAGCCATGAATGAATTCACATGTCAGACTTCAGAGATTTTTCCTGTCAGGAAAACTCTATGAGAACATATTCATGATTCTGAGCAC

>Ptr_miRNA_268

GAAGTTACAACGTAAAAAGATTCTTATTGTTCTTGATGATGTAGATGACC

>Ptr_miRNA_269

AATCACTACTTCTGAATTGGGACCTGATAGGGATACTATAAAAGTCCTGACGATTCTTCCACTGATTCT

>Ptr_miRNA_271

CTGTATTATCATGTTGCGTGTTTCCAGTAGATGCGATGATGATAGAACAGTA

>Ptr_miRNA_272

ACTCTCCCTCAAGGCTTCCAACGCAATAACCAGCTACGTACATGTAACTGTGTTACGGCTGCAGGCTTGAGGCCTTTGGGGGAGAGTGG

>Ptr_miRNA_275

GGAATGTTGTCTGGCTCGAGGACTTTTTGTTCATCAATCTAATCGAACTTTCTACCTGTAGATCTAGTATCTTATTTAAGATTGATCACGTATTAGGGTTGTCGGACCAGGCTTCATTCCCC

>Ptr_miRNA_277

TGGAGAAGCAGGGCACGTGCAAAATCCTTCTCGGCTTCCAGATGCTGATGAAGCACTCTTTGCACGTGCTCCCCTCCTCCAAC

>Ptr_miRNA_278

GGAGCTATCGATGCTGAATAATCATTTGCACGTCAGTCTGATCCTG

>Ptr_miRNA_279

TTTGCACGTCAGTCTGATCCTGAAATTACACTGATTATGACTATTTTTCTACCTTTTCATTCTCATTGGGATTTCTACGTGTGTTTT

>Ptr_miRNA_282

TGCTCCTTTTAGTCCAATAAGGAGGGCTGAGAAGCGGCTAGAGCTGCCATCTCATGCATTTAGGCAATGCTTAACATTTGGCAAAGAGGTTTAAGCTTGGCCAGGTGCATGGTGTGGGAGCAACTCCTTCCGCATGCTTTGTTCGCCCATTGGACTGAAGGGAGCTCC

>Ptr_miRNA_283

TTCATTCCTCTTCCTAAAATGGCTTCTTCTCCTTTAGTTGTTGCAATTATAGGAGAGGAGAATCCATTTTAGGAAGGGAATGAATA

>Ptr_miRNA_284

TGTTGGGATGGCTCAATCATATCAAATCTCCCAAACTATGATGTTGGGTCGTTTAATCTGATTGAGCCGTGCCAATATC

>Ptr_miRNA_285

TAGCCAAGGACGACTTGCCTATTTCCTCCATGGGGTTCTGAAAAGAATGAAATACTGTCGTTCAGAGCTCACTGGTAGGGTTCATAGGCAGTCTCCTTTGGCTATC

>Ptr_miRNA_286

TTCTGAACTCTCTCCCTCAACTGCTATTCTAGAGCTTGTTAATCCTTGTCAAGTGTTTCCCTGTAGCAGTCGAGGCGAGGGGGTTTCGGAAAA

>Ptr_miRNA_289

TCCAAAGGGATCGCATTGATCTAATGACTCTCGATGTCTAAATCATATTAATGTTTAGTTTTTTCGTTGGATCATGCGATCCCTTAGGAAT

>Ptr_miRNA_291

TTCTGAACTCTCTCCCTCAACTGCTACTCTAGATCTTGCCAATCCCTGTCAAGTGTTTCTGTGTAGCAGTCGAGGTGAGAGGTTCAGGAAA

>Ptr_miRNA_292

CGTGGACTGCAGCGCGCGCCGTCACGGCGTGCCTCGGCATATGTG

>Ptr_miRNA_293

AATGTCGTCTGACTCGAGACAACAGGTAAACTGAAGGAATGGCCGGAGTTTAAGAGTTCTCTCGGACCAGGCTTCATTCC

>Ptr_miRNA_294

CCGGACGGGATCTCAGGTTAGCCCAACCACACAAAGGATATTGGTTCTGGTTAAAGGGGATCGCCAGATGGGA

>Ptr_miRNA_295

CATGAAAGTTCTAGGGAATTGTCTCATCTTTCCAGGGTAAACATTTGAGATCATTTGGACTTCTAGAACTCGAGAT

>Ptr_miRNA_296

TTGACAGAAGATAGAGAGCACAGATGATGATATGCAATGGACTCTGCATCCCACTCCTTTGTGCTCTCTATGCTTCTGTCATCA

>Ptr_miRNA_298

AGCTCCTTGAAGTCCAATAGAAGCTCCTGCTGGGTAGATCGAGCTGCTGAGCTATGAATCCCACAGCCCTATCACCATCAGTCATTTTGATGGGCCTGCGGCTTGCATATCTCAGGAGCTTTATTACCTAATGTTAGATCTTTTTTTGGATTGAAGGGAGCTCT

>Ptr_miRNA_299

TGAATGTTGTCTGGTTCAAGGCATGGCCACCACATCTCTTGGTGAATATATGTCCTCGGACCAGGCTTCATTCCCC

>Ptr_miRNA_301

TTTAGTTGAGATTGGTTTGGAAAAATATATGTTTGGTTAAAATTGTGGTTAAAATTGAGGTTGAACAAAAAATAGTTTAATGTGTTTGGTTAAGAA

>Ptr_miRNA_302

GGATTCCAAATCGACTTAAAATATTTTCTTTCAAGAACTTTATCACTGTTTTATGGTCATTGTTTCGACTTGGAATTGCCT

>Ptr_miRNA_303

GAAAGATTCACCGACGGAGCATTTTCGTCGATGATTTTGTTGGTAAATTAATTACTGACGGAATATGTGTCTTACATCGACGGAAAAATTCCGTCGGTAAAACTGTTAA

>Ptr_miRNA_306

GGAGGCAGCGGTTCATCGATCTTTTCCTGAAGATTTTTTTGTTTTACACGAACAACACGAACCGATCGATAAACCTCTGCATCCAG

>Ptr_miRNA_307

AAGCTCAGGAGGGATAGCGCCCTAAGGATAACCATGGGCTCTTTTTATGTGGTTTTTGACTATCAGTGGCGCTATCCATCCTGAGTTTTA

>Ptr_miRNA_309

GTTTCCCTGACCACTTCACTGGGGCTATAATTCTACTCTTCGTGTTTAAGGGTAGAGGCAACCCTTCCTCACTGAAGTGTTTGGGGGAACTC

>Ptr_miRNA_311

TAATCTGCATCCTGAGGTTTGGATCATCATGTAGTTTGATCTAGTCCTTGGGTTGCAGATTACC

>Ptr_miRNA_312

GGATTCCAAACCGACTCAAAATATTTTCTTTCAAAAACTTTATCACTGTTTTGTGATCATTGTTTCGACTTGGAATTGCCT

>Ptr_miRNA_313

TGAAAGTTCTAGGAAATTGTCTCAGCTTTCCAACAAAAAAGAATCAGTGCATTTGGACTTCTAGAACTCGAGAT

>Ptr_miRNA_320

GCATCAGTTTAGGACTTCAAAAGGTGGCCAACAACACTTGTTTCTCTCCAAAGGTCTTGCAGTAAACCTAAACATCAAACCATACTATCTATAAAAAGATCCTTCAAACTGTTTGAGATTTAGTTACCGATTTTTTTGAGAGAGAAAAAAACTGTTCTTTTCTTCCTTTTGAAACTCCAAACTAATGCCT

>Ptr_miRNA_321

TTCGGCAGCGAATCACAGTTTCGCTGCGCAACACACAATTCGGCAGCGAATGCTAATTCGCTGCAGAAGA

>Ptr_miRNA_322

AACGGTCGGATCAAAAGTTATGGCCCTTTTCAGCTGTTATTCCGGTCTGGCACGACAA

>Ptr_miRNA_323

AACGGTCGGATCAAAAGTTATGGCATTTTTCAGCTGTTATTCCGGTCTGGCACCACAAGATCTCCTCTTTAACTAGTAGGGATTCCATATGTCGTACAAGGAAAAAGTCCTCCGCAAACTGCTGTAAATATTTGAGAGCGATCCAACG

>Ptr_miRNA_324

AACGGTCGGATCAAAAGTTATGGCGCTATTCAGCTGTTATTCCGGTCTGGCGCGACGA

>Ptr_miRNA_325

AACGGTCGGATCAAAAGTTATGGCGCTATTCAGCTGTTATTCCGGTCTGGCGCGACTA

>Ptr_miRNA_326

TCTGTCGCTGGAAAGATGGTACCTAGGGCTGCATATTAATGTGTATAGCAAGCAATAGGCACCATCTTCCCGCGACGGAAC

>Ptr_miRNA_328

GGCATGAGGTGTTTGGCAAGAAAATGGATCTTTTCCTTATGATGATTTCTTACCAATACCTCTCATGCCAA

>Ptr_miRNA_329

TTGACAGAAGATAGAGAGCACAGATGATGAAATGCATGGAGCTTAATTGCATCTCACTCCTTTGTGCTCTCTAGTCTTCTGTCATCA

>Ptr_miRNA_330

TATGGGAGAGGCGGGAATGACTCTGAAAGAACTTAGAAGAGTCTTTCCGAGTCCTCCCATACC

>Ptr_miRNA_331

CAGTTGCGATCTAAGGCAGGAGCCAAAAACAGAAGAAACGCTTGCTGGAATTGAGGTTGATAAACTTAATTATGGCTGGAGATTTGTTGGTTGTGGAACAGGTTCTGAATGCACAAATTGACGTTAGTGAT

>Ptr_miRNA_332

TGAACAGTGTAGTCATGCTCCACTTTTCACGTGAACAGTGGAGCATGACTACACTATTCATG

>Ptr_miRNA_333

TTAAAGGAGATCGCCAGAAGGGATCTCATGTTTTGGATTTGGTTAAAGGAGGTCGCCAGAAGGGATCTTATATTTCAGATTTGGTTAAAGGAGGTCACCAGAAGGGATCTCATATTTCAGC

>Ptr_miRNA_334

CTCGTTATCTAATTAGTAACGCACATGAATGGATTAACGAGAT

>Ptr_miRNA_335

TTTAGAATTTATGTTGTTGGATGTTTTGTTTAAAATATTTTAGAATTTAATTTATGTTGTTGGATGTTTTGTTTAAAATATTTTAGAATTTATATTTGGTTTGTGTTTTGGATA

>Ptr_miRNA_336

TTATCATATGCTGATCTGAGCCTCACTTTTCTCTCTATCTGTCTTTTACTGCGTCCCTTCTTTGTGTTTTTTTTTTACCAATTACAATGTTTGGATGCAGCTGGGTTAAGGAGGAAAACAAGAATTGGGAAAAAGATTACGACATTAGGATAAAT

>Ptr_miRNA_337

TTCCACAGCTTTCTTGAACTGCACCTATTAGATTAATGTTGATGTTGTTGTGCGATATGCCATGACCATATGACATTGTATTCATTTTTGCTGCGGTTCAATAAAGCTGTGGGAAG

>Ptr_miRNA_339

CGATGTTGGTGAGGTTCAATCCGAAGACAGATTTACACGAGTGAAAGTAAAATCCGATCTCAGATTGAGCCGCGCCAATATCACT

>Ptr_miRNA_341

GTTTAATCTCGCAAGTCCTGGTCATGCTTTTCCACAGCTTTCTTGAACTT

>Ptr_miRNA_342

TGACAGAAGAGAGTGAGCACACACGAAAGCTAATTGTATGAAAGCATACCATTGCAGGGTGTGTGCTCACTTCTCTTCTGTCAGC

>Ptr_miRNA_344

CAATTGCCCACAACTTTCATGAACAAAGGGCTCAAATTCTGATGTTAGCAATGATG

>Ptr_miRNA_346

AACGGTCGGATCAAAAGTTATGGCCTTTTCAGCTTTTAATCCGGTCTGCCGC

>Ptr_miRNA_347

AACGGTCGGATCAAAAGTTATGGCCATTTTCACCCGTCATTCCGGTCTGGCTCCACAAGGCCTCCTCTTCAACTAGTAAGGATTCCATAGGTACTACAAGGAAAACAGCCTCCGGAAACTACAGTAAAAAATATGAGCGCGATCCAATGGTCGG

>Ptr_miRNA_348

AACGGTCGGATCAAAAGTTATGGCCCCACTCAGCTGTTATTCCGGTCTGGCGCGACAA

>Ptr_miRNA_349

AACGGTCGGATCAAAAGTTATGGCCCTTTTCAACTGTTATTCTGGTCTGGAGCGACAAGACCTCATATTCATCGAGTGGGGATTCCATCGGTAATACAAGGAAAATGGCCTCCGGAAACTACTGTAAAACTTTCATGGCGATCCAACTGTCGA

>Ptr_miRNA_350

AACGGTCGGATCAAAAGTTATGGCCCTTTTCAGCTGTTATTCCGGTCTGGCGCGACAA

>Ptr_miRNA_351

AACGGTCGGATCAAAAGTTATGGCCCTTTTCGGCTGTTATTCCGGTCTGGCGCGACAA

>Ptr_miRNA_352

AACGGTCGGATCAAAAGTTATGGCTCTTTTCAGCTGTTATTCTGATCTGGCGCGACAA

>Ptr_miRNA_353

AACGGTCGGATCAAAAGTTATGGCCCTTTTCAGCTGTTATTCCAGTCCGGCGCGACAA

>Ptr_miRNA_354

AACGGTCGGATCAAAAGTTATGGCCCTTTTCAGCTGTTATTCCGGTCCGGCGCGACAA

>Ptr_miRNA_355

AACGGTCGGATCAAAAGTTATGGCCTTTTTCAGCTGTTATTCCGGTCTGGCGCGACAA

>Ptr_miRNA_356

TGACAGAAGAGAGTGAGCACACAGAGGCATATTTGTATAAAATTATACCATTGCTTTTGCGTGCTCATTTCTCTTTCTGTCACT

>Ptr_miRNA_357

GCTTGCTTGTTTTCTTTCCCGTTTTTTCCAGCTTCTGGGACATTAGAAATTGACAGAAGAGAGTGAGCAC

>Ptr_miRNA_359

AACGGTCGGATCAAAAGTTATGGCCTTTTCGAACAGGTACTCTGGTCTGGCGCGTCGA

>Ptr_miRNA_360

AACGGTCGGATCAAAAGTTATGGCACTTTTGACCCAATACTCCAGTTTGACCGACCAGACA

>Ptr_miRNA_361

AACGGTCGGATCAAAAGTTATGGCCCTCTTAAGCCAATACTCAAGTTCGACCGACCA

>Ptr_miRNA_362

AACGGTCGGATCAAAAGTTATGGCCCTTTTGAGTCGATACTTAAGTTCGACCGACCA

>Ptr_miRNA_363

AACGGTCGGATCAAAAGTTATGGCCCTTTTGAGTCAATACTTAAGTTCGACCGACCA

>Ptr_miRNA_364

CTCTGTACTCCATTCACTTAAATCGAGTCACATTTACTAATTGACAAGAAAACAATATGTGAACATGGAAACAAAATCATATCAACTTTCAATGTTATTGTCGATTAGTACACATGGCTTGATTTAAGGGGATGGAAGATGGAGTG

>Ptr_miRNA_366

TGAACAGTGTAGTCATGCTCCACTGTTCACGTGAACAGTGGAGCATGCCTCCACTGTTCATG

>Ptr_miRNA_367

CCGGATGGGATCTCAGGTTAGCCCAACCACACACAGGATTTTGGTTCTGGTTAAAGGGGATCGCCAGATGGGA

>Ptr_miRNA_368

GAGCTTCCTTCAGTCCACTCATGGACGGGCGAAGGGTTTGGATTAGCTGCCGACTCATTCATTCAAACACAGTAGACAAGGAGTGGCAGCGGCTGCTATTGTGAATGTGTGAATGACGCGGGAGATTAATTTCATCCTTTTCTTCTCTGTGCTTGGACTGAAGGGAGCTCCC

>Ptr_miRNA_369

TAGTAATCCTTCTTTGCAAAGTCATTTATTATTTTTCCTTCAATTACTATAATCAATGACTTTGCAAAGATAGATTTACTAGC

>Ptr_miRNA_370

TGAAAGTTCTAGGAAATTGTCTCAGCTTTCCAACAAAAAAAGAATCAGTGCATTTGGACTTCTAGAACTCGAGAT

>Ptr_miRNA_371

AACGGTCGGATCAAAAGTTATGGCCCTTTTGAGCCGATACTGAAGTTCGACCGAATC

>Ptr_miRNA_372

AACGGTCGGATCAAAAGTTATGGCCCTTGTTAGCCAATACTCAAGTTCGACCGACCA

>Ptr_miRNA_375

AACGGTCGGATCAAAAGTTATGGCCCTTATGAGCCGATACTCATGGTTGCCGACCAGATG

>Ptr_miRNA_376

AACGGTCGGATCAAAAGTTATGGCCATTTTGAGCCGATACTGAAGTTCGACCGACCA

>Ptr_miRNA_377

AACGGTCGGATCAAAAGTTATGGCCCTTTTGAGCCGATACTCTTATTAGACCGACCAGATA

>Ptr_miRNA_380

AATGAAGTTTGATCCAAGATCCTTGTCTCTCCCGTTAACTTAGTCTCTGTTACTGTTAGGTTTTCATTACTGAGTGTATTTGCAGCCCCCTTGTCTGATTTTAGCATCATGAGAGTAGGAAGTGCTGGTGATCATAGGGTTTGGTTCAAGATCCATTTG

>Ptr_miRNA_381

GCGTCGTGGTGATCGTCCTCCTGGGTCGGGATCTCTGGTGATGGAGGAAAGAATAATAAATTCTTGTTTCTTATCAATAAAAAGGGGATGAAGTTGTCACCTAGTATTTTGGTCACTAGGAACCC

>Ptr_miRNA_382

ATCTAGTGAAAGGACTCAAGCTCGTGATAGATAATTATTTTCTTTGAGTCCTTCCATTAGATCC

>Ptr_miRNA_383

TGAAAGTTCTAGGAAATTGTCTCAGCTTTCCAACAAAAAATGAATCGGTGCATTTGGACTTCTAGAACTCGAGAT

>Ptr_miRNA_385

TTTGAGCTCATAGTCCTTATGAAAGTTGTGGGTAATTGTCTCAGCTTGTCAACAAAAAAAAACCCAGAGCTCATTTGGACTTCTAGAACTCGAGAT

>Ptr_miRNA_386

AACGGTCGGATCAAAAGTTATGGCCATTTCGAACCGTTACTCAACCCTGGCGCGACCAGACCTCATCATCACCAAGTATCCATTCCTCATCCACTCCATGGAAAAGGGCATCCGGAAACTCCAATAAGAGTTTGACCGCGATCCAACGGTCGGTTCAAAAGTTATGGCCATTTCGAACAGTTATTCAAGCGTGG

>Ptr_miRNA_387

AACGGTCGGATCAAAAGTTATGGCCATTTCGAACCGTTACTCAAGCCTGGCGCGACCAGACCTCATCATCACCGAGTATCCATTCCTCATCCTCTCCATGGAAAAGGGCATCCGGAAACTCCAGTAAGAGTTTGACCGCGATCCAACGGTCGGATCAAAAGTTATGGCCATTTCGAACCGTTAGTCAAGTGTGG

>Ptr_miRNA_388

TTTCCGTTGACCAACTTTTCTAATAGCAAACAAACACAGGAAAGTTTAGAAAGTGGTTTCTCGAAAAT

>Ptr_miRNA_389

TTCGGCAGCGAATCACAGTTTCGCTGCATAACACACAATTCGGCAGCGAATGCTAATTCGCTGCAGAAGAC

>Ptr_miRNA_391

AACGGTCGGATCAAAAGTTATGGCCCTTCTCAGCTGTTATTCTGGTCTGGCGCGACAA

>Ptr_miRNA_394

AACGGTCGGATCAAAAGTTATGGCCCTTTTCAGCTGTTATTCTGGTCTGGCACGACAA

>Ptr_miRNA_395

AACGGTCGGATCAAAAGTTATGGCGCTATTCAGCTGTTATTCCGGTCTGGTGCGACAAGACC

>Ptr_miRNA_396

AACGGTCGGATCAAAAGTTATGGCCCTTTTCAGCTGTTATTCCGGTCTGGCACCACA

>Ptr_miRNA_399

AACGGTCGGATCAAAAGTTATGGCCCTTTCGAACCGGTTCTCTGGTCTGGAGCGACGAGACTTCCAATTCACCGAGTA

>Ptr_miRNA_400

ACTCGAACCTGAGACCACAGAGAGATCAAACCTCTTAGTCCGAGGGGGAGTAT

>Ptr_miRNA_401

TCCATGTATATCGAGAGATTTTAATTCTGGGAGACTCTGGAATTATTTCCCTTTCACCATTCTCTTCTCTGATAATATGCTTC

>Ptr_miRNA_402

AATTCTGATGTTAGCAATGATGTTTTCTACAGACAAGAAGATAAGGATTGTTACCAAGTCA

>Ptr_miRNA_403

AACGGTCGGATCAAAAGTTATGGCCATTTCGAACCGTTACTCAAGCCTGGAGCGACCTGCCCTCATCATCACCAAGTATCGATTCCTCATCCACTCCATGCAAAAGGGCATTCGGAAAGTCCAGTAAGAGTTTGACCGCGATCCAACGGTAGGATCAAAAGTTATGGCTATTTCAAACCGTTAC

>Ptr_miRNA_404

AACGGTCGGATCAAAAGTTATGGCCCTTTTCACCTTTTATTCCGGCCTGGCGCGACATGACATCCTCTTCAACGAATTGGGATTCCATAGGTACAACAAGGAAAATGGCCTCCGGAAACTACTTTAAAACTTTGAGCGCGATCCAACGGTCGGATCAAAAGTTATGGCCCTTTTCACCTTTTATTCCGGCCTGGCG

>Ptr_miRNA_405

AACGGTCGGATCAAAAGTTATGGCCCTTTTCACCTTTTATTCCGGCCTGGCG

>Ptr_miRNA_406

AACGGTCGGATCAAAAGTTATGGCCCTTTTCACCTTTTATTCCGGCCTGG

>Ptr_miRNA_407

TGAAGCTGCCAGCATGATCTATCTTTGGTTAGAGAAGGATAGAAGCGAAAGAACTAACCCTAGCTAGGTCATGCTCTGACAGCCTCACT

>Ptr_miRNA_409

TTGTCGGCACCGGACTGTGAGCTTTGTGTAAACCGCTTAGACGTCACGTGCTCAATG

>Ptr_miRNA_412

AACGGTCGGATCAAAAGTTATGGCCCTTTTCAACAGTTATTCCGGTCTGGAGCGACAAGAGCCTCCTATTCATCCGAGTCAGGG

>Ptr_miRNA_413

AACGGTCGGATCAAAAGTTATGGCCGTTTCAAGGAAAAAGGTCTCCGGAAACTACTTACGAACTATGAGTGTGATCCAACTGTTGG

>Ptr_miRNA_414

AACGGTCGGATCAAAAGTTATGGCCCTTTTCAGCTGTTATTCAGGTCCGGCGCGACAA

>Ptr_miRNA_417

AGAAGCATTTTACCTGAAGGGAACAGGCTGAAAGAGAACTTCTAT

>Ptr_miRNA_418

AACGGTCGGATCAAAAGTTATGGCCCTTTTCAGCTGTTATTCCGATCTGG

>Ptr_miRNA_419

AACGGTCGGATCAAAAGTTATGGCCCTTTTCAACAGTTATTCTGGTCTAGAGCGACAAGACCTCCTATTCATCGAGTAGGGATTCCATCGGTAATACAAGGAAAATGGCCTCCAGAAACTACTGTAAAACTTTCATGGCGTCCAACGGTCGG

>Ptr_miRNA_420

AACGGTCGGATCAAAAGTTATGGCTCTTCTCAGCTGTTATTCCGGTCTGGCGCGACAA

>Ptr_miRNA_421

TTGTAAGAGCCTGGGACAGCCGGGGTTTTACTCGCTCACCTGGGCCCACAAAG

>Ptr_miRNA_422

AACGGTCGGATCAAAAGTTATGGCCATTTCGAACCGTTACTCAAGCCTGGCGCGACCAGACCTCATCATCACCGAGTATCGATTCCTTATCCACTTCATGAATTAGGGCATTCAGAAACTCCAGTAAGAGTTTGATCGCGATCCAACGGTCAGAACAAAAGTTATGGCCATTTCGAATCGTTACTCAAGCCTGG

>Ptr_miRNA_423

AACGGTCGGATCAAAAGTTATGGCCCTTTCGAACCGGTACTCTAGTCTGGCGCGTCGA

>Ptr_miRNA_424

CATTTCGAACCGTTACACAAGCCTGGCGCGACCAGACCTTAAAATCACCGAGTATCCATTCCTCATCCACTCAGTGGAAAAGGGCATCTGGAAAACTCCAGTAAGAGTTTGATCGCGATCCAACGGTCGGATCAAAAGTTATGGC

>Ptr_miRNA_425

AACGGTCGGATCAAAAGTTATGGCCATTTCGAACCGTTACTCAAGCCTGGAGCGACCAGCCCTCATCATCACCGAGTATCGATTCCTCATCCACTCCATGCAAAAGGGCATGCGGAAAGTCCAGTAAGAGTTTGACCGCGATCCAACGGTCGGATCACAATTATGGCCATTCGAACCGTCTCAAGCCTGG

>Ptr_miRNA_426

TCAACAACTTCAATACCATAACAAATCAATTGTCATCTGTTGAGATTGAGTTTGATG

>Ptr_miRNA_427

CAAGCCATAGGATGTCGTACCCCGAAGATAACGCATGATGCGTTTCATAGCAGCCCCAATGAGAATCTGTAGGAGCATGCA

>Ptr_miRNA_428

TTACCGACGGATAATTGAATATTAATATTTTTTAATTATTCCATCTGTAATTCCATCGGTAATA

>Ptr_miRNA_430

GGATTCCAAATCGACTCAAAATATTTTCTTTCAAAAACTTTATCACTGTTTTGTGATCATTGTTTCGACTTGGAATTGCCT

>Ptr_miRNA_431

AACGGTCGGATCAAAAGTTATGGCCCTTTTGAGCCGATACTCAAGTTCGACCGACCA

>Ptr_miRNA_432

ATGGTTCTTTGACATGGTATCAGAGCCTTGATAATCAAGCGGTCACGAGTTCGAATCTCACCATCC

>Ptr_miRNA_433

TGACAGAAGAGAGTGAGCACACAGGGTACTTTCTTGCATGACGTTCATGCTTGAAGCTTTGCGTGCCCACCCTCTATCTGTCACC

>Ptr_miRNA_436

TTGAGGACCAAATTTGATATAATCAACAAATAATATGATATTTTTAAATTTTTCACAAC

>Ptr_miRNA_438

AAGATCTGTGCACTGTGAAGAGAAAACTGAATGTGAAAAAGAAAGCCTTTTTAGCCGAACAAGTAAGTGCCATTCTTCAGAACAATAATGCTTTGAAATATAAAGACCCTGGTTGTCCTACAATTTCTTGCTTTATTGGAGAACATAAAATTGAAAGAGCTTTACTTGATCTTGGAGCTAGTGTGAATTTACTTCCATATTCAGTCTTTCAGAGTCTCAATCTAGG

>Ptr_miRNA_440

CGTAAGAGTTTGACCGCGATCCAACGGTCGGATCAAAAGTTATGGC

>Ptr_miRNA_442

TTACCGACGGATAATTGAATATTAATATTTTTTAATTATTCTGTCGGTAATT

>Ptr_miRNA_443

CAGTAACAGTTTGACCGCTATACAACGGTCGGATCAAAAGTTATGGC

>Ptr_miRNA_444

CATTTTCAACGTCGTCTCAAAAGAATTTAAATTTTCAAATGTATTTTGAACACGTTTAACTTTTAACGCGTTATTTTTACAATCATTGATTTTTCTCGTTGAGTCGACGTTGATATTGCT

>Ptr_miRNA_445

CATTTCGAACCGTTACTCAAGCCTGGCGCGATCAGACCTCATTATCACCGAGTATCGATTCGTCATCCAGTCCATGGAAAAGGGCATGCGGATAGTCCAGTAAGAGTTTGACCGCGATCCAACGGTCGGATCAAAAGTTATGGC

>Ptr_miRNA_446

AACGGTCGGATCAAAAGTTATGGCCCTTTTAAGCCGATACTTAAGTTCGACCGATCA

>Ptr_miRNA_447

AACGGTCGGATCAAAAGTTATGGCCCTTTTAAGCCGATACTTAAGTTCGACCGACCA

>Ptr_miRNA_455

TTTACCGACGGATAATTGAATATTAATATTTTTTATTTATTCCGTCGGTAATTC

>Ptr_miRNA_456

AACGGTCGGATCAAAAGTTATGGCCCTTTTGAGCCGATACTCATGTTCGACCGACTG

>Ptr_miRNA_457

CCTTTTGAGCTGATTGTCAAGGTTGCCAACCAGATGTCCTCGTCACTGAACAACGATTCAATAACCTAACCAAAGACAATAGACTCTGGAAACCCCTATAAAAATTTGAGCGCGATCCAACGGTCGGATCAAAAGTTATGGC

>Ptr_miRNA_458

AAGGAAGTTTGAGGCAATAACAGGTCTGTGATGCCCTTAGATGTTCTGGG

>Ptr_miRNA_459

GGACTTTTCTTGAACCATAGACGGACCAACATATATAGACATTAGAATGGTTAGGTTTTACCCGATAAGATAAGGACCTC

>Ptr_miRNA_460

TATTTGTAAAGCCATGTAGATCAAAGGAGGAACCACGAGTGATATGTAAGCCATAGGTGGTCGTACCCTGTAGATAACGTAGAATACATTTAACGGCACCCCAATGAGAATCTGTAGGAGCATGCA

>Ptr_miRNA_461

ATTGGATCACGGTGTAGAAGTACAAACTTGTACGAAAGTTGTATTTCTAAATTCCGATGAAAAGATAA

>Ptr_miRNA_462

TGTATGTTTTGGATTGTTTTGATGTGCTGATGTCAAAAATGATTTTTAAAAAATAAAAAAATATTATTGGCATGCATTTAGGCACGAAAAGTTATTTGAAAAGCACC

>Ptr_miRNA_463

ACCGTTAGAAAGATCTCGACAAGATGAATTCAATGAGACCAAAGGTCACAGATGGTGAATGGAATGGGCCACACGAGTTATCCAAAGGTGTCGGGGCTCACTTGCTTTTGGGCTGCTTGTGTGACCCACTCTAATTACCGTTGATGACCTTTCTTGGTATCGCTGGATTCATTTTGTCAAGATTTTTTTAACGATAC

>Ptr_miRNA_464

TGTGTGTGTGTGTGTGTGAATAATAAGATATGCATATATTATATTGTATTTTAAAAGAAATATAAATGTTCTATATGTATTTATATTGTCGCCACCTAACACCGAGA

>Ptr_miRNA_465

AACGGTCGGATCAAAAGTTATGGCCCTTTTCAGCTTTTATTCCGGCATGG

>Ptr_miRNA_466

CCAAATTGAATTTTAATTATGTTCAAGAATTAATTTGGGTCCAATTGAAGGATTTAATTAAGTGCAAGGACTTAATTATACTTTAAGCGGGTCAAATTAATTTGAGGGCTTAATTGGTGAAAAATTAAGTTTGAGAGCTTAATTTGGACTTAATTGAAAAATCAGAATTTTAAGAGACCCAATTTAATTTTTACCAAGTGAATTGATTGAAATTAGGGGCC

>Ptr_miRNA_467

TATAACCCGTCGGTATTTCACAGAGAGTTGCAAAAAAAATTACGGGATTTTGCCACATTTACCGACGGATATACCGACGGAATATAAC

>Ptr_miRNA_469

AACCCGTCGGTATTTCACAGAGAGTTGCAAAAAAATTACGGGATTGTGACACAATCACCGACGGGTACACCGACCGATGTAAAATACCGACGGAATCA

>Ptr_miRNA_470

AAGATCTGTGCACTGTGAAGAGAAAACTGAATGTGAAAAAGAAAGCCTTTTTAGCTGAACAAGTAAGTGCCATTCTTCATAACAATAATGCTTTGAAGTATAAAGACCCTGGTTGTCCTACAATTTCTTGCTTTATTGGAGAACATAAAATTGAAAGAGCTTTACTTGATCTTGGAGCTAGTGTGAATTTACTTCCATATTCAGTCTTTCAGAGTCTCAATCTAGG

>Ptr_miRNA_471

TGCTGGTTGTCTTAAATTGCTAAACATTTATTAGCTTATGCTATGATAATTTGTTTATAATATTCTTGACTCTGGCGCC

>Ptr_miRNA_472

TTGTTTCGACTTGGAATTGCCTCTATCCATTTTGAAACATAATTTATTGCGACTAATATGTACAAGAAACCAAATGATGGAGGAAATGGACCCATGAAATCTAT

>Ptr_miRNA_473

TGAAAGTTCTGGGAAATTGTCTCCGCTTTTCAAGAAAACAAGAATGAGCTCATTTGGACTTCTAGAACTCGAGAT

>Ptr_miRNA_474

GGATTCCAAATCGACTCAAAATATTTTCTTTCAGAACTTTATCACTGTTTTATGGTCATTGTTTCGACTTGGAATTGCCT

>Ptr_miRNA_475

TAGTATTTTGGTCACTAGGAACCCTAACTGGTCTTAGAGATCGGCTACG

>Ptr_miRNA_476

TTAAAGGAGATCGCCAGAAGGGATCTCATGTTTTGGATTTGGTTAAAGGAGGTCGCCAGAAGGGATCTTATATTTCAGATTTGGTAAAAGGAGGTCACCAGAAGGGATCTCATATTTTAGC

>Ptr_miRNA_477

GTGAGCCGAGTCCAATGGAGCTGTTTGTAGAGACGCATGTGCGGAGTTAAGACCGCCAAAAGGGGGTGCAGCAGTTCGTGGACAACCGTGCTCAGCA

>Ptr_miRNA_478

ATGCTAAAAACGATGCAAATGTATTAAAAACGCATTTTTTTAGATTTTCAATGTTTTTCGCTATTT

>Ptr_miRNA_480

AAGGACTAACTTGTAAAAGGCGCCGAAATGCAGGGCCAATTACAGTTTAAACCAGGG
