# Supplementary material for: Identification of Populus Small RNAs Responsive to Mutualistic Interactions With Mycorrhizal Fungi, Laccaria bicolor and Rhizophagus irregularis
Source: Front Microbiol. 2019 Mar 18;10:515. doi: 10.3389/fmicb.2019.00515 (PMC6431645; doi:10.3389/fmicb.2019.00515)
Supplement: Supplementary file 4 [file Table_4.DOCX]

*Populus deltoides* mature miRNA sequences

>Pde_miRNA_1

AAAAATTCCGTCGGTAAAACTGTT

>Pde_miRNA_2

AGTAGGATTTCTGTTTATGATACT

>Pde_miRNA_3

AAAAGTTGGTCAACGGAAAACACT

>Pde_miRNA_4

AAATCACCGACGGATTGAAAAGTC

>Pde_miRNA_5

AAATTCCGTCGGTAAAACTGTTAA

>Pde_miRNA_6

AACGGTCGGATCAAAAGTTATGGC

>Pde_miRNA_7

AACGGTCGGATCAAAAGTTATGGC

>Pde_miRNA_8

AACGGTCGGATCAAAAGTTATGGC

>Pde_miRNA_9

AACGGTCGGATCAAAAGTTATGGC

>Pde_miRNA_10

AACGGTCGGATCAAAAGTTATGGC

>Pde_miRNA_11

AACTCGAGATATGGGCTGAACACT

>Pde_miRNA_12

AGCGGGGTAGAGTAATGGTTAACT

>Pde_miRNA_13

AGCTGTCAGACCTAACACTCTTGG

>Pde_miRNA_14

AAGATCTGTGCACTGTGAAGAGAA

>Pde_miRNA_15

AAGATCTGTGCACTGTGAAGAGAA

>Pde_miRNA_16

AAGCTCAGGAGGGATAGCGCC

>Pde_miRNA_17

AAGCTCAGGAGGGATAGCGCC

>Pde_miRNA_18

AAGCTCAGGAGGGATAGCGCC

>Pde_miRNA_19

AAGCTCAGGAGGGATAGCGCC

>Pde_miRNA_20

AAGGACTAACTTGTAAAAGGCGCC

>Pde_miRNA_21

AAGGCAACAAATTAGAGTCTCTGA

>Pde_miRNA_22

AAGGCTCTGATACCATGTTAAAAA

>Pde_miRNA_23

TTACAAGCATGTGCATTTAGAGGT

>Pde_miRNA_24

AATCACTACTTCTGAATTGGGACC

>Pde_miRNA_25

TCTCGGACCAGGCTTCATTCC

>Pde_miRNA_26

AATGGCCATTGTAAGAGTAGA

>Pde_miRNA_27

ACCGAACCGAACCGTGAACACCCC

>Pde_miRNA_28

TCCACATTCGGTCAATGTTCC

>Pde_miRNA_29

AGTGACGCGCATGAATGGATT

>Pde_miRNA_30

ACCCAAGTTTAAGTGAGTCTGGCT

>Pde_miRNA_31

ACGTACTAATTTTGATTTTTGAAG

>Pde_miRNA_32

ACTCATCATCTTTCTCACAAGGTT

>Pde_miRNA_33

ACTCATCATCTTTCTCACAAGGTT

>Pde_miRNA_34

ACTCCCCCTCAAGGGCTTCCTGTT

>Pde_miRNA_35

ACTCTCCCTCAAGGCTTCCAA

>Pde_miRNA_37

ATTAGTCGAGATACACGCAAGCTG

>Pde_miRNA_38

AGAATCTTGATGATGCTGCAT

>Pde_miRNA_39

GATCATACCAGCACTAATGCACC

>Pde_miRNA_40

AGCCATTTTATCAACGGACAACT

>Pde_miRNA_41

TCCATGGAAGATAATGACTCG

>Pde_miRNA_42

TTTGGATTGAAGGGAGCTCT

>Pde_miRNA_43

TTTGGATTGAAGGGAGCTCT

>Pde_miRNA_44

CGCCAAAGGAGAGTTGCCCTC

>Pde_miRNA_45

AGTAAGTAGACATATTGATAAAGT

>Pde_miRNA_46

AGTAGGATTTTCGGAAGGTTATCG

>Pde_miRNA_47

AGTGTCTGGGCTGCAGGACAGATT

>Pde_miRNA_48

TTTACCCGATAAGATAAGGACCTC

>Pde_miRNA_49

TTAGATTCACGCACAAACTCG

>Pde_miRNA_50

ATAATATTCCTGACTCTGGCGCCA

>Pde_miRNA_51

ATAATATTCCTGACTCTGGCGCCA

>Pde_miRNA_52

TCGCTCCTGATCGAACAAGTCT

>Pde_miRNA_53

ATCAATAGTTCTGAATTTTGGCCT

>Pde_miRNA_54

ATCACGAGCCATCATAACTGT

>Pde_miRNA_55

AGGCGCCATGGGTCTGGGAGGTGC

>Pde_miRNA_56

ATGAAAAGGACTTTGAAAAG

>Pde_miRNA_58

AGCTGCCGACTCATTCATTC

>Pde_miRNA_59

TGAAGCTGCCAGCATGATCT

>Pde_miRNA_60

ATTCTATGGTACGAGACTGGTTAC

>Pde_miRNA_61

ATTTGAGTTTTGTAGGCGTTAGCT

>Pde_miRNA_62

ATTTGCACGTCAGTCTGATCCTG

>Pde_miRNA_63

ATTTGGACTTCTAGAACTCGAGAT

>Pde_miRNA_64

ATTTGGACTTCTAGAACTCGAGAT

>Pde_miRNA_65

ATTTGGACTTCTAGAACTCGAGAT

>Pde_miRNA_66

ATTTGGACTTCTAGAACTCGAGAT

>Pde_miRNA_67

ATTTGGACTTCTAGAACTCGAGAT

>Pde_miRNA_68

ATTTTAGGAAGGGAATGAATA

>Pde_miRNA_69

CCTTCCAAGCAATAGACCCG

>Pde_miRNA_70

ATTTTTATTCCTGACTCTGGCGTC

>Pde_miRNA_71

ATTGGGTTGAGATGGTTCTTTGAC

>Pde_miRNA_72

CAAATTCTGATGTTAGCAATGATG

>Pde_miRNA_73

CAAATTCTGATGTTAGCAATGATG

>Pde_miRNA_75

AGTTACTAATTCATGATCTGG

>Pde_miRNA_76

CACATTCGGTCAACGTTCGAG

>Pde_miRNA_77

ATTTGGACTTCTAGAACTCGAGAT

>Pde_miRNA_78

ATTTGGACTTCTAGAACTCGAGAT

>Pde_miRNA_81

CCACACATGTGATTGCTGGCT

>Pde_miRNA_82

ACTTCTAGAACTCGAGATATGGGC

>Pde_miRNA_83

TGTGAGGACCATGATATGAGT

>Pde_miRNA_84

CCTAATGACGAACTATTTTGACA

>Pde_miRNA_85

CCTTATGAGAAATCAAAGTTTT

>Pde_miRNA_86

TTGAGCCGCGCCAATATCACT

>Pde_miRNA_87

TTGAGCCGCGCCAATATCACT

>Pde_miRNA_88

TGCACTGCCTCTTCCCTGGC

>Pde_miRNA_89

AACGGTCGGATCAAAAGTTATGGC

>Pde_miRNA_90

GTGATCCAAATGCTCTGAAATGTT

>Pde_miRNA_91

ATTTGGACTTCTAGAACTCGAGAT

>Pde_miRNA_92

ATTTTTATTCCTGACTCTGGCGTC

>Pde_miRNA_95

CTGCTCGCAAGTCGGAGGCCT

>Pde_miRNA_96

ATTTGGACTTCTAGAACTCGAGAT

>Pde_miRNA_97

CTTCGCGTCCAGCGGTGCGGGCAC

>Pde_miRNA_98

ACTTCTAGAACTCGAGATATGGGC

>Pde_miRNA_99

AGACCGGGGTCGAACCACAGAGAG

>Pde_miRNA_100

CTTTTTGGATCGTTTTGATGTGCT

>Pde_miRNA_101

TCTCTTATACGTTTTTGTCTC

>Pde_miRNA_102

TCTCTTATGCGTTTTTGTCTC

>Pde_miRNA_103

TTCGCTCGTCGGGATTGTTGT

>Pde_miRNA_104

TTGTTTCGACTTGGAATTGCCT

>Pde_miRNA_105

ATTGGAGTGAAGGGAGCTCGA

>Pde_miRNA_106

TTGGACTGAAGGGAGCTCCC

>Pde_miRNA_107

TCTCGGACCAGGCTTCATTCC

>Pde_miRNA_108

TTTTCCCAACTCCACCCATCCC

>Pde_miRNA_109

AGAATCTTGATGATGCTGCAT

>Pde_miRNA_110

ATCAATAGTTCTGAATTTTGGCCT

>Pde_miRNA_111

ATCAATAGTTCTGAATTTTGGCCT

>Pde_miRNA_112

GGAATCTTGATGATGCTGCAT

>Pde_miRNA_113

ATTCCTGAGCGGTTATCTGAGCAA

>Pde_miRNA_114

TCGAATTTGGGCTTGAGATT

>Pde_miRNA_115

AGGCTTGATAAATCATTCCGGACC

>Pde_miRNA_116

TGACAGAAGAGAGTGAGCAC

>Pde_miRNA_117

TGACAGAAGAGAGTGAGCAC

>Pde_miRNA_118

TGACAGAAGAGAGTGAGCAC

>Pde_miRNA_119

GCTTGGGCGAGAGTAGTACTAGG

>Pde_miRNA_120

GCTTGGGCGAGAGTAGTACTAGG

>Pde_miRNA_121

TCGGACCAGGCTTCATTCCCC

>Pde_miRNA_122

TCGGACCAGGCTTCATTCCCC

>Pde_miRNA_123

TCGGACCAGGCTTCATTCCCC

>Pde_miRNA_124

GGAATCTTGATGATGCTGCAG

>Pde_miRNA_125

GGAATCTTGATGATGCTGCAG

>Pde_miRNA_126

AGAATCTTGATGATGCTGCAT

>Pde_miRNA_127

TGTGTTCTCAGGTCGCCCCTG

>Pde_miRNA_128

TTTGCACGTCAGTCTGATCCTG

>Pde_miRNA_129

TCGATAAACCTCTGCATCCAG

>Pde_miRNA_130

TCGATAAACCTCTGCATCCAG

>Pde_miRNA_131

TGTGTTCTCAGGTCACCCCTT

>Pde_miRNA_132

TTGTTTCGACTTGGAATTGCCT

>Pde_miRNA_133

TTGTTTCGACTTGGAATTGCCT

>Pde_miRNA_134

TTGTTTCGACTTGGAATTGCCT

>Pde_miRNA_135

TTGTTTCGACTTGGAATTGCCT

>Pde_miRNA_136

TTGTTTCGACTTGGAATTGCCT

>Pde_miRNA_137

TTGTTTCGACTTGGAATTGCCT

>Pde_miRNA_138

TTGTTTCGACTTGGAATTGCCT

>Pde_miRNA_139

TCGGACCAGGCTTCATTCCCC

>Pde_miRNA_140

TCGGACCAGGCTTCATTCCCC

>Pde_miRNA_141

TTACCAATACCTCTCATGCCAA

>Pde_miRNA_143

TTTTCCCTACTCCACCCATCCC

>Pde_miRNA_144

TTAGATTCACGCACAAACTCG

>Pde_miRNA_145

TTAGATTCACGCACAAACTCG

>Pde_miRNA_146

GTAACCAGGCTCTGATACCA

>Pde_miRNA_147

ACTAGGACGGTCTGAGGCTT

>Pde_miRNA_148

ACTAGGACGGTCTGAGGCTT

>Pde_miRNA_149

TAAGAATGAAGAGATTGTGAACCT

>Pde_miRNA_150

GTGCTTATCGACGGTCTTGTGCT

>Pde_miRNA_151

CGAGCCGAATCAATATCACTC

>Pde_miRNA_152

TGCGATGATGATAGAACAGTA

>Pde_miRNA_153

TTTCGCTGCCGCAACTGAACCT

>Pde_miRNA_154

ACAGATTATTCAGAATTCTAGCCT

>Pde_miRNA_155

ACAGATTATTCAGAATTCTAGCCT

>Pde_miRNA_156

GTTTAATATCTGATACGTGGGC

>Pde_miRNA_157

TAAGAATGAAGAGATTGTGAACCT

>Pde_miRNA_158

GTCCTTGGGTTGCAGATTACC

>Pde_miRNA_159

GTCCTTGGGTTGCAGATTACC

>Pde_miRNA_160

TGATTGAGCCGTGCCAATATC

>Pde_miRNA_161

TAATTGATTATTGATGTAGTGGCC

>Pde_miRNA_163

GAGGATTGTACAAAGTGAAGAAAT

>Pde_miRNA_164

ACTGAGAGCTCTTTCTTGATT

>Pde_miRNA_165

TAGGCAGTCTCCTTTGGCTATC

>Pde_miRNA_166

TAGGCTGGAAACCTGTGACCCGAT

>Pde_miRNA_167

TAGTATTTTGGTCACTAGGAACCC

>Pde_miRNA_168

TAGTATTTTGGTCACTAGGAACCC

>Pde_miRNA_169

TAGTATTTTGGTCACTAGGAACCC

>Pde_miRNA_170

TGAGACGCATCCTAGTGGAGA

>Pde_miRNA_171

TCTTTCCGAGTCCTCCCATACC

>Pde_miRNA_172

TCTTTCCAACGCCTCCCATACC

>Pde_miRNA_173

TATTCCGTCGGTAATTAATTTACC

>Pde_miRNA_174

TGATTGAGCCGTGCCAATATC

>Pde_miRNA_175

TGATTGAGCCGTGCCAATATC

>Pde_miRNA_176

AAAAATTCCGTCGGTAAAACTGTT

>Pde_miRNA_177

CACAAGCGATCTAGTTGGCT

>Pde_miRNA_178

ATGATCCAATGTTTTCTGATGATT

>Pde_miRNA_179

CTTGTATTTAAGTCTCTGATGATT

>Pde_miRNA_181

TCCAAAGGGATCGCATTGATCT

>Pde_miRNA_182

TCCAAAGGGATCGCATTGATCT

>Pde_miRNA_183

TCGCTTGGTGCAGGTCGGGAA

>Pde_miRNA_184

GTGGGAATGAACATTATGAGA

>Pde_miRNA_185

ATGGAGACCTGAACTGAAGAGA

>Pde_miRNA_186

TCTGTCGCTGGAAAGATGGTACC

>Pde_miRNA_187

CGAGATATGGCTAAAATACTG

>Pde_miRNA_188

ATTTGGACTTCTAGAACTCGAGAT

>Pde_miRNA_189

TTACAATGTCCATTGATTAAG

>Pde_miRNA_190

TGAAGCTGCCAGCATGATCT

>Pde_miRNA_191

TGAAGCTGCCAGCATGATCT

>Pde_miRNA_192

TGAAGCTGCCAGCATGATCT

>Pde_miRNA_193

TGAAGCTGCCAGCATGATCT

>Pde_miRNA_194

TGAAGCTGCCAGCATGATCTG

>Pde_miRNA_195

TGAAGCTGCCAGCATGATCTG

>Pde_miRNA_196

AGATCATGTGGCAGTTTCACC

>Pde_miRNA_197

AGATCATGTGGCAGTTTCACC

>Pde_miRNA_198

TCGGACCAGGCTTCATTCCCC

>Pde_miRNA_199

TGACAGAAGAGAGTGAGCAC

>Pde_miRNA_200

TGACAGAAGAGAGTGAGCAC

>Pde_miRNA_201

TGACAGAAGAGAGTGAGCAC

>Pde_miRNA_202

TGACAGAAGAGAGTGAGCAC

>Pde_miRNA_203

TGACAGAAGAGAGTGAGCAC

>Pde_miRNA_204

TGACAGAAGAGAGTGAGCAC

>Pde_miRNA_205

TGACAGAAGAGAGTGAGCAC

>Pde_miRNA_206

TGCAGTCAAGTAATCATCAAG

>Pde_miRNA_207

CGGAGATGATGAATATGCACT

>Pde_miRNA_208

TTCATGAAAGTTGTTGGCAATC

>Pde_miRNA_209

TGCATTTGCACCTGCACCTTA

>Pde_miRNA_210

TGCCTGGCTCCCTGAATGCCA

>Pde_miRNA_211

TGCCTGGCTCCCTGTATGCCA

>Pde_miRNA_212

TGCCTGGCTCCCTGTATGCCA

>Pde_miRNA_213

TGCCTGGCTCCCTGTATGCCA

>Pde_miRNA_214

TGCCTGGCTCCCTGTATGCCA

>Pde_miRNA_215

TGCCTGGCTCCCTGTATGCCA

>Pde_miRNA_216

TGCTGAAATCTTGAGACACAG

>Pde_miRNA_217

ATGGTCTGCGAGAGTCTGAGTAAG

>Pde_miRNA_218

ATTTGGACTTCTAGAACTCGAGAT

>Pde_miRNA_219

TGGAGAAGCAGGGCACGTGCA

>Pde_miRNA_220

TGGAGAAGCAGGGCACGTGCA

>Pde_miRNA_221

TGGAGAAGCAGGGCACGTGCA

>Pde_miRNA_222

TGGAGAAGCAGGGCACGTGCA

>Pde_miRNA_223

GAAAAGTTATTTGAAAAGCACCCG

>Pde_miRNA_224

TGTGTTCTCAGGTCACCCCTT

>Pde_miRNA_225

TGTTATCAATTTCTGTCTGAGCCT

>Pde_miRNA_226

ACGAAAAGTTATTTGAAAAGCACC

>Pde_miRNA_227

TGATTGAGCCGTGCCAATATC

>Pde_miRNA_228

TGATTGAGCCGTGCCAATATC

>Pde_miRNA_229

TGATTGAGCCGTGCCAATATC

>Pde_miRNA_230

TGATTGAGCCGTGCCAATATC

>Pde_miRNA_231

TTAAAACTCGAAATCGGTCAAACT

>Pde_miRNA_232

TTAAAACTCGAAATCGGTCAAACT

>Pde_miRNA_233

TTAAAACTCGAAATCGGTCAAACT

>Pde_miRNA_234

ACTTCTAGAACTCGAGATATAGGC

>Pde_miRNA_235

TTAAGGCTCTGATACCATGTTAAA

>Pde_miRNA_236

TTACCGACGGATAATTGAATATT

>Pde_miRNA_237

TTACCGACGGATAATTGAATATT

>Pde_miRNA_238

TTACCGACGGATAATTGAATATT

>Pde_miRNA_239

TTACCGACGGATAATTGAATATT

>Pde_miRNA_240

TTGAGGACCAAATTTGATATAATC

>Pde_miRNA_241

TTCATTCCTCTTCCTAAAATGG

>Pde_miRNA_242

TTCATTCCTCTTCCTAAAATGG

>Pde_miRNA_243

TTCCACAGCTTTCTTGAACTG

>Pde_miRNA_244

TTCCACAGCTTTCTTGAACTG

>Pde_miRNA_245

TTCCACAGCTTTCTTGAACTG

>Pde_miRNA_246

TTCCACAGCTTTCTTGAACTT

>Pde_miRNA_247

TTCCACAGCTTTCTTGAACTT

>Pde_miRNA_248

TTCCGAGATTCTGGACAGAAT

>Pde_miRNA_249

TATGGAATGCTTTCTGGTTGGAGC

>Pde_miRNA_250

TTCGACCGTTGATTGATGAGT

>Pde_miRNA_251

TTCGGCAGCGAATCACAGTTTCGC

>Pde_miRNA_252

TTCTGAACTCTCTCCCTCAAC

>Pde_miRNA_253

TTCTGAACTCTCTCCCTCAAC

>Pde_miRNA_254

TTCTGAACTCTCTCCCTCAAC

>Pde_miRNA_255

TTCTGAACTCTCTCCCTCAAC

>Pde_miRNA_257

TTGACAGAAGATAGAGAGCAC

>Pde_miRNA_258

TTGACAGAAGATAGAGAGCAC

>Pde_miRNA_259

TTGACAGAAGATAGAGAGCAC

>Pde_miRNA_260

TTGACAGAAGATAGAGAGCAC

>Pde_miRNA_261

TTGACAGAAGATAGAGAGCAC

>Pde_miRNA_262

TTGAGGACCAAATTTGATATAATC

>Pde_miRNA_263

TTGAGGACCAAATTTGATATAATC

>Pde_miRNA_264

TTGAGGACCAAATTTGATATAATC

>Pde_miRNA_265

TTGATTGATTGATTGATTGAT

>Pde_miRNA_266

AATTATTTTTGAGATCAGCGCATC

>Pde_miRNA_267

ACAGAAGAGAGTGAGCACAC

>Pde_miRNA_268

TTGGATTCCTGACTTAAAGAC

>Pde_miRNA_269

TTGGCATTCTGTCCACCTCC

>Pde_miRNA_270

ATTTGGACTTCTAGAACTCGAGAT

>Pde_miRNA_271

AATTAGTCGAGATACATGTAAACT

>Pde_miRNA_272

TTTACCCGATAAGATAAGGACCTC

>Pde_miRNA_273

TTTACCCGATAAGATAAGGACCTC

>Pde_miRNA_274

TTTACCCGATAAGATAAGGACCTC

>Pde_miRNA_275

TTTACCGACGGATAATTGAATATT

>Pde_miRNA_276

TTTACCGACGGATAATTGAATATT

>Pde_miRNA_277

TTTACCGACGGATAATTGAATATT

>Pde_miRNA_278

TTTACCGACGGATAATTGAATATT

>Pde_miRNA_279

TTTACCGACGGATAATTGAATATT

>Pde_miRNA_280

TTTACCGACGGATAATTGAATATT

>Pde_miRNA_281

TTTACCGACGGATAATTGAATATT

>Pde_miRNA_283

TTTCCAAAATTATCTTCGTCGATT

>Pde_miRNA_284

TTTCCACGGCTTTCTTGAACT

>Pde_miRNA_285

TTTCGCTGCCGCAACTGAACCT

>Pde_miRNA_286

TTTCGCTGCCGCAACTGAACCT

>Pde_miRNA_287

TTTCTTCACGAAACCTGTTTGC

>Pde_miRNA_288

TTTGCACGTCAGTCTGATCCTG

>Pde_miRNA_289

TTTGCACGTCAGTCTGATCCTG

>Pde_miRNA_290

TTGAGGAGAATGAGCAAGGG

>Pde_miRNA_291

TTAGATGACCATCAACGAAAA

>Pde_miRNA_292

TTGAGGAGAATGAGCAAGGGG

>Pde_miRNA_293

TAGTATTTTGGTCACTAGGAACCC

>Pde_miRNA_294

aagatctgtgcactgtgaagagaa

>Pde_miRNA_295

ttacaagcatgtgcatttagaggt

>Pde_miRNA_296

atttttattcctgactctggcgtc

>Pde_miRNA_297

taggctggaaacctgtgacccgat

>Pde_miRNA_298

tagtattttggtcactaggaaccc

>Pde_miRNA_299

ttaccgacggataattgaatatt

*Populus trichocarpa* mature miRNA sequences

>Ptr_miRNA_1

TGAGACGCATCCTAGTGGAGA

>Ptr_miRNA_2

ATTTGGACTTCTAGAACTCGAGAT

>Ptr_miRNA_3

ACGAAAAGTTATTTAAAAAGCACC

>Ptr_miRNA_4

TTAAAACTCGAAATCGGTCAAACT

>Ptr_miRNA_5

TCGGACCAGGCTTCATTCCCC

>Ptr_miRNA_7

GGCGACATGGCCGAGTGGTAAGGC

>Ptr_miRNA_8

TTAAAACTCGAAATCGGTCAAACT

>Ptr_miRNA_9

TTTCTTCACGAAACCTGTTTGC

>Ptr_miRNA_10

AACAAGGTCTTTTTTAGTCAGATT

>Ptr_miRNA_11

ACTTCTAGAACTCGAGATATGGGC

>Ptr_miRNA_12

TTTACCCGATAAGATAAGGACCTC

>Ptr_miRNA_13

AAGCTCAGGAGGGATAGCGCC

>Ptr_miRNA_15

ACGAAAAGTTATTTGAAAAGCACC

>Ptr_miRNA_16

ACTTCTAGAACTCGAGATATGGGC

>Ptr_miRNA_17

AAAGAGAGAGAGAGAGAGAGA

>Ptr_miRNA_18

AAATTCCGTCGGTAAAACTGTTAA

>Ptr_miRNA_19

TGCCTGGCTCCCTGTATGCCA

>Ptr_miRNA_21

TCTTCTCTGATAATATGCTTC

>Ptr_miRNA_22

ATTCATGGCACAACTGATATTGGT

>Ptr_miRNA_23

TTACCGACGGATAATTGAATATT

>Ptr_miRNA_25

TGCAGTCAAGTAATCATCAAG

>Ptr_miRNA_27

TGATTGAGCCGTGCCAATATC

>Ptr_miRNA_28

TGTGTTCTCAGGTCGCCCCTG

>Ptr_miRNA_29

GTGGGAATGAACATTATGAGA

>Ptr_miRNA_30

TGATTCCTTCCAATTCCATTTG

>Ptr_miRNA_32

TTTCTGTGGTGGTTTGGAAGC

>Ptr_miRNA_33

TCCATGGAAGATAATGACTCG

>Ptr_miRNA_41

TCCATGGAAGATAATGACTCG

>Ptr_miRNA_43

TTTCCAAAATTATCTTCGTCGATT

>Ptr_miRNA_44

ATTTGGACTTCTAGAACTCGAGAT

>Ptr_miRNA_45

TGGAGAAGCAGGGCACGTGCA

>Ptr_miRNA_46

TTTCTTCACGAAACCTGTTTGC

>Ptr_miRNA_47

CCGGACGGGATCTCAGGTTAGCCC

>Ptr_miRNA_48

TCGGACCAGGCTTCATTCCCC

>Ptr_miRNA_49

CCCTATGAGGAAGCAATCCCA

>Ptr_miRNA_50

AATTATTTTTGAGATCAGCGCATC

>Ptr_miRNA_51

TTAAACCTGAACATTGCAAAGAAA

>Ptr_miRNA_52

CTTGTATTTAAGTCTCTGATGATT

>Ptr_miRNA_53

TGAAGCTGCCAGCATGATCT

>Ptr_miRNA_55

TGAAGCTGCCAGCATGATCT

>Ptr_miRNA_56

TTGGCATTCTGTCCACCTCC

>Ptr_miRNA_57

TGATTGAGCCGTGCCAATATC

>Ptr_miRNA_58

ACGAAAAGTTATTTGAAAAGCACC

>Ptr_miRNA_59

ACTCCCCCTCAAGGGCTTCCTGTT

>Ptr_miRNA_60

TGCACTGCCTCTTCCCTGGC

>Ptr_miRNA_61

TTAGATTCACGCACAAACTCG

>Ptr_miRNA_62

ACTTCTAGAACTCGAGATATGGGC

>Ptr_miRNA_63

TGGAGAAGCAGGGCACATGCT

>Ptr_miRNA_65

TCGAATTTGGGCTTGAGATT

>Ptr_miRNA_66

TTCCACAGCTTTCTTGAACTT

>Ptr_miRNA_67

AGAGTCATGCCGGATTAACCTGTT

>Ptr_miRNA_68

CGCCAAAGGAGAGTTGCCCTC

>Ptr_miRNA_69

AAAAGTTGGTCAACGGAAAACACT

>Ptr_miRNA_70

TGAGTCGACGTTGATATTGCT

>Ptr_miRNA_71

TTAACCGAGAAAACCGAACCGA

>Ptr_miRNA_72

AAATCGGTCAAACTCGGTCAAACT

>Ptr_miRNA_74

TAATTGATTATTGATGTAGTGGCC

>Ptr_miRNA_75

TGACGCATGGATTCGACAGCCC

>Ptr_miRNA_76

AGGGACTCAGAAATCATCAACGAA

>Ptr_miRNA_77

ATTTGGACTTCTAGAACTCGAGAT

>Ptr_miRNA_78

AGAATCTTGATGATGCTGCAT

>Ptr_miRNA_79

TCGCTTGGTGCAGGTCGGGAA

>Ptr_miRNA_80

AAATTCCGTCGGTAAAACTGTTAA

>Ptr_miRNA_81

ATTTGGACTTCTAGAACTCGAGAT

>Ptr_miRNA_84

ACTTCTAGAACTCGAGATATGGGC

>Ptr_miRNA_85

AATTCCATCGGTAAAACTGTTAAA

>Ptr_miRNA_86

TGATTGAGCCGTGCCAATATC

>Ptr_miRNA_88

TTGTTTCGACTTGGAATTGCCT

>Ptr_miRNA_89

CTGCCCGAGGTGCACGATTTGG

>Ptr_miRNA_90

TATATATATTTTGTGGGGACCC

>Ptr_miRNA_91

ATTCCTGAGCGGTTATCTGAGCAA

>Ptr_miRNA_92

TTGTTTCGACTTGGAATTGCCT

>Ptr_miRNA_93

TGAAGCTGCCAGCATGATCT

>Ptr_miRNA_94

TTGTATTAGCTCTATCTGATCATT

>Ptr_miRNA_95

ATGATCCAATGTTTTCTGATGATT

>Ptr_miRNA_96

TCTCTTATGCGTTTTTGTCTC

>Ptr_miRNA_100

TCTCTTATACGTTTTTGTCTC

>Ptr_miRNA_102

TTGGCATTCTGTCCACCTCC

>Ptr_miRNA_103

TCGGACCAGGCTTCATTCCCC

>Ptr_miRNA_105

TTTTCCCTACTCCACCCATCCC

>Ptr_miRNA_106

AGTAGGATTTTCGGAAGGTTATCG

>Ptr_miRNA_107

AATCACTACTTCTGAATTGGGACC

>Ptr_miRNA_108

AGGGATATAACTCAGCGGTAGAG

>Ptr_miRNA_109

TGAAGCTGCCAGCATGATCTG

>Ptr_miRNA_110

ACTTCTAGAACTCGAGATATGGGC

>Ptr_miRNA_112

TCGGACCAGGCTTCATTCCCC

>Ptr_miRNA_113

TCGGACCAGGCTTCATTCCCC

>Ptr_miRNA_116

GTGCTTATCGACGGTCTTGTGCT

>Ptr_miRNA_117

TTTACCGACGGATAATTGAATATT

>Ptr_miRNA_118

ATTTGGACTTCTAGAACTCGAGAT

>Ptr_miRNA_120

ATTTGGACTTCTAGAACTCGAGAT

>Ptr_miRNA_122

TGCCTGGCTCCCTGAATGCCA

>Ptr_miRNA_124

TGTTATCAATTTCTGTCTGAGCCT

>Ptr_miRNA_125

TTATCAATTTCTGTCTGAGCCTA

>Ptr_miRNA_126

TTGGGCTGAAGGGAGCTCCC

>Ptr_miRNA_127

TGACAGAAGAGAGTGAGCAC

>Ptr_miRNA_128

GACTTGAAATCTGTTGGGCT

>Ptr_miRNA_129

TTTCCACGGCTTTCTTGAACT

>Ptr_miRNA_130

TGACAGAAGAGAGTGAGCAC

>Ptr_miRNA_131

TGACAGAAGAGAGTGAGCAC

>Ptr_miRNA_133

TTCCACAGCTTTCTTGAACTG

>Ptr_miRNA_134

ATTTGGACTTCTAGAACTCGAGAT

>Ptr_miRNA_135

AACTTGATGTGAAAACTGCCT

>Ptr_miRNA_136

ATTAATCGAGGTACGCACAAACTG

>Ptr_miRNA_137

GTCCTTGGGTTGCAGATTACC

>Ptr_miRNA_138

ATCACGAGCCATCATAACTGT

>Ptr_miRNA_140

TTGAGCCGCGCCAATATCACT

>Ptr_miRNA_142

TTCCACAGCTTTCTTGAACTT

>Ptr_miRNA_143

AACGGACCGAGATTTAAAATGAGA

>Ptr_miRNA_144

AAGCTCAGGAGGGATAGCGCC

>Ptr_miRNA_146

ATTAGTCGAGATGCACATAAACTG

>Ptr_miRNA_147

TAGTATTTTGGTCACTAGGAACCC

>Ptr_miRNA_148

TCGGACCAGGCTTCATTCCCC

>Ptr_miRNA_150

TTAGATGACCATCAACGAAAA

>Ptr_miRNA_151

CTAGTTCACATGTTGCTATCAACT

>Ptr_miRNA_152

GACATATAATTAGTTCTGAGGCTT

>Ptr_miRNA_154

ACTTCTAGAACTCGAGATATGGGC

>Ptr_miRNA_155

ATTTGGACTTCTAGAACTCGAGAT

>Ptr_miRNA_156

ATTTGGACTTCTAGAACTCGAGAT

>Ptr_miRNA_157

AGGGATGTAGCGCAGCTTGGTAGC

>Ptr_miRNA_159

ACAGATTATTCAGAATTGTAGCCT

>Ptr_miRNA_160

TATTCCGAGATTCTGAACAGA

>Ptr_miRNA_161

GATTAGTCGAGATGCGCGCAAGCT

>Ptr_miRNA_162

TTGAGACGCATCCTAGTGGAGA

>Ptr_miRNA_163

ATTTGGACTTCTAGAACTCGAGAT

>Ptr_miRNA_164

TCGCAAGTCGGAGGCCTGGCT

>Ptr_miRNA_165

GGAATCTTGATGATGCTGCAG

>Ptr_miRNA_167

GAGTCCCTAGGGGCTGTTTGGGCT

>Ptr_miRNA_168

TGTGAGGACCATGATATGAGT

>Ptr_miRNA_169

TCTCGGACCAGGCTTCATTCC

>Ptr_miRNA_170

TCCACATTCGGTCAATGTTCC

>Ptr_miRNA_171

AATGGCCATTGTAAGAGTAGA

>Ptr_miRNA_172

AACAAATCTCTCCCTCTCACCCC

>Ptr_miRNA_174

TGCCTGGCTCCCTGTATGCCA

>Ptr_miRNA_176

TTAGATTCACGCACAAACTCG

>Ptr_miRNA_178

TCTTTCCAACGCCTCCCATACC

>Ptr_miRNA_179

AGATCATGTGGCAGTTTCACC

>Ptr_miRNA_180

ATTAGTCGAGGTACGCGCAAGCTG

>Ptr_miRNA_181

CGGTTCGGTTCGGTTTCGGTT

>Ptr_miRNA_182

TGTGTTCTCAGGTCGCCCCTG

>Ptr_miRNA_183

AGAATCTTGATGATGCTGCAT

>Ptr_miRNA_185

TGCATTTGCACCTGCACCTTA

>Ptr_miRNA_186

CCGACCTTAGCTCAGTTGGT

>Ptr_miRNA_187

GGAATCTTGATGATGCTGCAT

>Ptr_miRNA_188

AAGCTCAGGAGGGATAGCGCC

>Ptr_miRNA_190

TCTCGGACCAGGCTTCATTCC

>Ptr_miRNA_191

TTACAATGTCCATTGATTAAG

>Ptr_miRNA_193

AAAAACTGAAACCGAACCGAAACC

>Ptr_miRNA_194

TTCGGTTCGGTTCGGTTTTTATC

>Ptr_miRNA_195

TGTGTTCTCAGGTCACCCCTT

>Ptr_miRNA_196

ACTCGGATAGACTTAGGCTCGGTC

>Ptr_miRNA_197

TGAGTCGACGTTGATATTGCT

>Ptr_miRNA_198

TGCCTGGCTCCCTGTATGCCA

>Ptr_miRNA_200

AGAATCTTGATGATGCTGCAT

>Ptr_miRNA_201

AGAATCTTGATGATGCTGCAT

>Ptr_miRNA_202

GGAATCTTGATGATGCTGCAG

>Ptr_miRNA_204

TTAGATTCACGCACAAACTCG

>Ptr_miRNA_206

AGATCATGTGGCAGTTTCACC

>Ptr_miRNA_208

ATTTGGACTTCTAGAACTCGAGAT

>Ptr_miRNA_209

ACTAGGACGGTCTGAGGCTT

>Ptr_miRNA_210

ATTTGGACTTCTAGAACTCGAGAT

>Ptr_miRNA_211

TGAGTCGACGTTGATATTGCT

>Ptr_miRNA_213

TGAGTCGACGTTGATATTGCT

>Ptr_miRNA_215

TTAACCGAGAAAACCGAACCGA

>Ptr_miRNA_216

TGAGTCGACGTTGATATTGCT

>Ptr_miRNA_219

ATAATATTCCTGACTCTGGCGCCA

>Ptr_miRNA_220

TGCCTGGCTCCCTGTATGCCA

>Ptr_miRNA_221

ATTAGTCGAGGTGCGCGCAAGCTG

>Ptr_miRNA_223

ATTAGTCGAGGTGCGCGCAAGCTG

>Ptr_miRNA_225

TTTTCACCGGAAGTAAGCCTC

>Ptr_miRNA_226

AATTTTCCGTCGGTATTTTCCAGA

>Ptr_miRNA_227

TGATTGAGCCGTGCCAATATC

>Ptr_miRNA_228

TCATTGAGTGCAGCGTTGATG

>Ptr_miRNA_230

AATTATTTTTGAGATCAGCGCATC

>Ptr_miRNA_231

TGCTATCAGAAACTAAGAACCT

>Ptr_miRNA_232

TTTACCCGATAAGATAAGGACCTC

>Ptr_miRNA_233

TTTACCGACGGATAATTGAATATT

>Ptr_miRNA_234

TGATTGAGCCGTGCCAATATC

>Ptr_miRNA_235

TTCTGAACTCTCTCCCTCAAC

>Ptr_miRNA_236

TCCAAAGGGATCGCATTGATCT

>Ptr_miRNA_238

TTCTGAACTCTCTCCCTCAAC

>Ptr_miRNA_239

TTGACAGAAGATAGAGAGCAC

>Ptr_miRNA_241

AAACGGATATCTTCGGTCGGTATT

>Ptr_miRNA_243

CGAGCCGAATCAATATCACTC

>Ptr_miRNA_245

TTGACAGAAGATAGAGAGCAC

>Ptr_miRNA_247

TTTGGATTGAAGGGAGCTCT

>Ptr_miRNA_248

TCGATAAACCTCTGCATCCAG

>Ptr_miRNA_250

TTGTTTCGACTTGGAATTGCCT

>Ptr_miRNA_251

CAGAATTGCAGTGCCTTGATT

>Ptr_miRNA_253

ACGTACTAATTTTGATTTTTGAAG

>Ptr_miRNA_254

ACGTACTAATTTTGATTTTTGAAG

>Ptr_miRNA_255

ATTTGGACTTCTAGAACTCGAGAT

>Ptr_miRNA_256

TGGAGAAGCAGGGCACGTGCA

>Ptr_miRNA_257

GCGCAACAGTTCGTTGATAACCGT

>Ptr_miRNA_258

TTTTCCCAACTCCACCCATCCC

>Ptr_miRNA_259

TTAACCGAGAAAACCGAACCGA

>Ptr_miRNA_260

AACCCGTCGGTATTTCACAGAGAG

>Ptr_miRNA_261

TAGTATTTTGGTCACTAGGAACCC

>Ptr_miRNA_262

ACTTCTAGAACTCGAGATATGGGC

>Ptr_miRNA_263

ACTTCTAGAACTCGAGATATGGGC

>Ptr_miRNA_264

CTGAGAGCTCTTTCTTGATTC

>Ptr_miRNA_267

ACATATTCATGATTCTGAGCAC

>Ptr_miRNA_268

TTCTTGATGATGTAGATGACC

>Ptr_miRNA_269

AATCACTACTTCTGAATTGGGACC

>Ptr_miRNA_271

TGCGATGATGATAGAACAGTA

>Ptr_miRNA_272

ACTCTCCCTCAAGGCTTCCAA

>Ptr_miRNA_275

TCGGACCAGGCTTCATTCCCC

>Ptr_miRNA_277

TGGAGAAGCAGGGCACGTGCA

>Ptr_miRNA_278

TTTGCACGTCAGTCTGATCCTG

>Ptr_miRNA_279

TTTGCACGTCAGTCTGATCCTG

>Ptr_miRNA_282

ATTGGACTGAAGGGAGCTCC

>Ptr_miRNA_283

TTCATTCCTCTTCCTAAAATGG

>Ptr_miRNA_284

TGATTGAGCCGTGCCAATATC

>Ptr_miRNA_285

TAGGCAGTCTCCTTTGGCTATC

>Ptr_miRNA_286

TTCTGAACTCTCTCCCTCAAC

>Ptr_miRNA_289

TCCAAAGGGATCGCATTGATCT

>Ptr_miRNA_291

TTCTGAACTCTCTCCCTCAAC

>Ptr_miRNA_292

CGTGGACTGCAGCGCGCGCC

>Ptr_miRNA_293

TCTCGGACCAGGCTTCATTCC

>Ptr_miRNA_294

CCGGACGGGATCTCAGGTTAGCCC

>Ptr_miRNA_295

ATTTGGACTTCTAGAACTCGAGAT

>Ptr_miRNA_296

TTGACAGAAGATAGAGAGCAC

>Ptr_miRNA_298

TTTGGATTGAAGGGAGCTCT

>Ptr_miRNA_299

TCGGACCAGGCTTCATTCCCC

>Ptr_miRNA_301

TTTAGTTGAGATTGGTTTGGAAA

>Ptr_miRNA_302

TTGTTTCGACTTGGAATTGCCT

>Ptr_miRNA_303

AAATTCCGTCGGTAAAACTGTTAA

>Ptr_miRNA_306

TCGATAAACCTCTGCATCCAG

>Ptr_miRNA_307

AAGCTCAGGAGGGATAGCGCC

>Ptr_miRNA_309

CTGAAGTGTTTGGGGGAACTC

>Ptr_miRNA_311

GTCCTTGGGTTGCAGATTACC

>Ptr_miRNA_312

TTGTTTCGACTTGGAATTGCCT

>Ptr_miRNA_313

ATTTGGACTTCTAGAACTCGAGAT

>Ptr_miRNA_320

TTTTGAAACTCCAAACTAATGCCT

>Ptr_miRNA_321

TTCGGCAGCGAATCACAGTTTCGC

>Ptr_miRNA_322

AACGGTCGGATCAAAAGTTATGGC

>Ptr_miRNA_323

AACGGTCGGATCAAAAGTTATGGC

>Ptr_miRNA_324

AACGGTCGGATCAAAAGTTATGGC

>Ptr_miRNA_325

AACGGTCGGATCAAAAGTTATGGC

>Ptr_miRNA_326

ACCATCTTCCCGCGACGGAAC

>Ptr_miRNA_328

TTACCAATACCTCTCATGCCAA

>Ptr_miRNA_329

TTGACAGAAGATAGAGAGCAC

>Ptr_miRNA_330

TCTTTCCGAGTCCTCCCATACC

>Ptr_miRNA_331

CAGTTGCGATCTAAGGCAGGA

>Ptr_miRNA_332

TGAACAGTGTAGTCATGCTCC

>Ptr_miRNA_333

TTAAAGGAGATCGCCAGAAGGG

>Ptr_miRNA_334

CATGAATGGATTAACGAGAT

>Ptr_miRNA_335

TTTAGAATTTATGTTGTTGGATGT

>Ptr_miRNA_336

TTATCATATGCTGATCTGAGCCT

>Ptr_miRNA_337

TTCCACAGCTTTCTTGAACTG

>Ptr_miRNA_339

TTGAGCCGCGCCAATATCACT

>Ptr_miRNA_341

TTCCACAGCTTTCTTGAACTT

>Ptr_miRNA_342

TGACAGAAGAGAGTGAGCAC

>Ptr_miRNA_344

CAAATTCTGATGTTAGCAATGATG

>Ptr_miRNA_346

AACGGTCGGATCAAAAGTTATGGC

>Ptr_miRNA_347

AACGGTCGGATCAAAAGTTATGGC

>Ptr_miRNA_348

AACGGTCGGATCAAAAGTTATGGC

>Ptr_miRNA_349

AACGGTCGGATCAAAAGTTATGGC

>Ptr_miRNA_350

AACGGTCGGATCAAAAGTTATGGC

>Ptr_miRNA_351

AACGGTCGGATCAAAAGTTATGGC

>Ptr_miRNA_352

AACGGTCGGATCAAAAGTTATGGC

>Ptr_miRNA_353

AACGGTCGGATCAAAAGTTATGGC

>Ptr_miRNA_354

AACGGTCGGATCAAAAGTTATGGC

>Ptr_miRNA_355

AACGGTCGGATCAAAAGTTATGGC

>Ptr_miRNA_356

TGACAGAAGAGAGTGAGCAC

>Ptr_miRNA_357

TGACAGAAGAGAGTGAGCAC

>Ptr_miRNA_359

AACGGTCGGATCAAAAGTTATGGC

>Ptr_miRNA_360

AACGGTCGGATCAAAAGTTATGGC

>Ptr_miRNA_361

AACGGTCGGATCAAAAGTTATGGC

>Ptr_miRNA_362

AACGGTCGGATCAAAAGTTATGGC

>Ptr_miRNA_363

AACGGTCGGATCAAAAGTTATGGC

>Ptr_miRNA_364

TTAAGGGGATGGAAGATGGAGTG

>Ptr_miRNA_366

TGAACAGTGTAGTCATGCTCC

>Ptr_miRNA_367

CCGGATGGGATCTCAGGTTAGCCC

>Ptr_miRNA_368

TTGGACTGAAGGGAGCTCCC

>Ptr_miRNA_369

TAGTAATCCTTCTTTGCAAAG

>Ptr_miRNA_370

ATTTGGACTTCTAGAACTCGAGAT

>Ptr_miRNA_371

AACGGTCGGATCAAAAGTTATGGC

>Ptr_miRNA_372

AACGGTCGGATCAAAAGTTATGGC

>Ptr_miRNA_375

AACGGTCGGATCAAAAGTTATGGC

>Ptr_miRNA_376

AACGGTCGGATCAAAAGTTATGGC

>Ptr_miRNA_377

AACGGTCGGATCAAAAGTTATGGC

>Ptr_miRNA_380

AATGAAGTTTGATCCAAGATC

>Ptr_miRNA_381

TAGTATTTTGGTCACTAGGAACCC

>Ptr_miRNA_382

TTGAGTCCTTCCATTAGATCC

>Ptr_miRNA_383

ATTTGGACTTCTAGAACTCGAGAT

>Ptr_miRNA_385

ATTTGGACTTCTAGAACTCGAGAT

>Ptr_miRNA_386

AACGGTCGGATCAAAAGTTATGGC

>Ptr_miRNA_387

AACGGTCGGATCAAAAGTTATGGC

>Ptr_miRNA_388

TTTCCGTTGACCAACTTTTCTAAT

>Ptr_miRNA_389

TTCGGCAGCGAATCACAGTTTCGC

>Ptr_miRNA_391

AACGGTCGGATCAAAAGTTATGGC

>Ptr_miRNA_394

AACGGTCGGATCAAAAGTTATGGC

>Ptr_miRNA_395

AACGGTCGGATCAAAAGTTATGGC

>Ptr_miRNA_396

AACGGTCGGATCAAAAGTTATGGC

>Ptr_miRNA_399

AACGGTCGGATCAAAAGTTATGGC

>Ptr_miRNA_400

ACTCGAACCTGAGACCACAGAGAG

>Ptr_miRNA_401

TCTTCTCTGATAATATGCTTC

>Ptr_miRNA_402

AATTCTGATGTTAGCAATGATGTT

>Ptr_miRNA_403

AACGGTCGGATCAAAAGTTATGGC

>Ptr_miRNA_404

AACGGTCGGATCAAAAGTTATGGC

>Ptr_miRNA_405

AACGGTCGGATCAAAAGTTATGGC

>Ptr_miRNA_406

AACGGTCGGATCAAAAGTTATGGC

>Ptr_miRNA_407

TGAAGCTGCCAGCATGATCT

>Ptr_miRNA_409

TTGTCGGCACCGGACTGTGAGC

>Ptr_miRNA_412

AACGGTCGGATCAAAAGTTATGGC

>Ptr_miRNA_413

AACGGTCGGATCAAAAGTTATGGC

>Ptr_miRNA_414

AACGGTCGGATCAAAAGTTATGGC

>Ptr_miRNA_417

AACAGGCTGAAAGAGAACTTCTAT

>Ptr_miRNA_418

AACGGTCGGATCAAAAGTTATGGC

>Ptr_miRNA_419

AACGGTCGGATCAAAAGTTATGGC

>Ptr_miRNA_420

AACGGTCGGATCAAAAGTTATGGC

>Ptr_miRNA_421

TTGTAAGAGCCTGGGACAGCC

>Ptr_miRNA_422

AACGGTCGGATCAAAAGTTATGGC

>Ptr_miRNA_423

AACGGTCGGATCAAAAGTTATGGC

>Ptr_miRNA_424

AACGGTCGGATCAAAAGTTATGGC

>Ptr_miRNA_425

AACGGTCGGATCAAAAGTTATGGC

>Ptr_miRNA_426

TCTGTTGAGATTGAGTTTGATG

>Ptr_miRNA_427

AATGAGAATCTGTAGGAGCATGCA

>Ptr_miRNA_428

TTACCGACGGATAATTGAATATT

>Ptr_miRNA_430

TTGTTTCGACTTGGAATTGCCT

>Ptr_miRNA_431

AACGGTCGGATCAAAAGTTATGGC

>Ptr_miRNA_432

ATGGTTCTTTGACATGGTATCAGA

>Ptr_miRNA_433

TGACAGAAGAGAGTGAGCAC

>Ptr_miRNA_436

TTGAGGACCAAATTTGATATAATC

>Ptr_miRNA_438

AAGATCTGTGCACTGTGAAGAGAA

>Ptr_miRNA_440

AACGGTCGGATCAAAAGTTATGGC

>Ptr_miRNA_442

TTACCGACGGATAATTGAATATT

>Ptr_miRNA_443

AACGGTCGGATCAAAAGTTATGGC

>Ptr_miRNA_444

TGAGTCGACGTTGATATTGCT

>Ptr_miRNA_445

AACGGTCGGATCAAAAGTTATGGC

>Ptr_miRNA_446

AACGGTCGGATCAAAAGTTATGGC

>Ptr_miRNA_447

AACGGTCGGATCAAAAGTTATGGC

>Ptr_miRNA_455

TTTACCGACGGATAATTGAATATT

>Ptr_miRNA_456

AACGGTCGGATCAAAAGTTATGGC

>Ptr_miRNA_457

AACGGTCGGATCAAAAGTTATGGC

>Ptr_miRNA_458

AAGGAAGTTTGAGGCAATAAC

>Ptr_miRNA_459

TTTACCCGATAAGATAAGGACCTC

>Ptr_miRNA_460

AATGAGAATCTGTAGGAGCATGCA

>Ptr_miRNA_461

ATTGGATCACGGTGTAGAAGTACA

>Ptr_miRNA_462

ACGAAAAGTTATTTGAAAAGCACC

>Ptr_miRNA_463

ACCGTTAGAAAGATCTCGACAAGA

>Ptr_miRNA_464

TATTGTCGCCACCTAACACCGAGA

>Ptr_miRNA_465

AACGGTCGGATCAAAAGTTATGGC

>Ptr_miRNA_466

GTGAATTGATTGAAATTAGGGGCC

>Ptr_miRNA_467

TATAACCCGTCGGTATTTCACAGA

>Ptr_miRNA_469

AACCCGTCGGTATTTCACAGAGAG

>Ptr_miRNA_470

AAGATCTGTGCACTGTGAAGAGAA

>Ptr_miRNA_471

TATAATATTCTTGACTCTGGCGCC

>Ptr_miRNA_472

TTGTTTCGACTTGGAATTGCCT

>Ptr_miRNA_473

ATTTGGACTTCTAGAACTCGAGAT

>Ptr_miRNA_474

TTGTTTCGACTTGGAATTGCCT

>Ptr_miRNA_475

TAGTATTTTGGTCACTAGGAACCC

>Ptr_miRNA_476

TTAAAGGAGATCGCCAGAAGGG

>Ptr_miRNA_477

GTGAGCCGAGTCCAATGGAGCTGT

>Ptr_miRNA_478

TTTTCAATGTTTTTCGCTATTT

>Ptr_miRNA_480

AAGGACTAACTTGTAAAAGGCGCC
