# Supplementary material for: Identification of Populus Small RNAs Responsive to Mutualistic Interactions With Mycorrhizal Fungi, Laccaria bicolor and Rhizophagus irregularis
Source: Front Microbiol. 2019 Mar 18;10:515. doi: 10.3389/fmicb.2019.00515 (PMC6431645; doi:10.3389/fmicb.2019.00515)
Supplement: Supplementary file 5 [file Table_5.DOCX]

**Coding sequences of small open-reading-frames (sORFs) in *Populus deltoids***

>Podel.CUFF.196.1

ATGGATTTTGAAATTAACAACCATGTAAACTTCCAGTGA

>Podel.CUFF.3016.1-sense

ATGTATCCTAAACAGAAATCCTACTTACGTTTTTTTTAG

>Podel.CUFF.830.1

ATGGTCCCGTGGTCTAGTGGTGAGGACATTGGACTCTGA

>Podel.CUFF.1615.1

ATGTTGCTGCCCAGGATCGAACTGGGGACCTTTAGTGTGTAA

>Podel.CUFF.1796.1

ATGATGGTTATATGTTTCATTCATATTCGGTCAACGTTCGAGTGA

>Podel.CUFF.2933.1

ATGGTTCCTAGGGTGGAACAGAGGGCAGATGCAACTCCTGGATGA

>Podel.CUFF.2671.1

ATGCAGGTTTCGAACCTGCGACTTCGCGTTATTAGCACGACGCTCTAA

>Podel.CUFF.1658.1

ATGGTCATTAACTTCAGAATTCATGAAATTAGTCGAGTTATGCCCAAACTAACCTAG

>Podel.07G087100.1.v2.1

ATGATGATGATAGATCCAAAGGCTTGTTTCTCAAGAACATTCGCAGTGGCCGCCTGA

>Podel.CUFF.2992.1

ATGGTGATGAAGGATTGTGTGGATAAGAGCATAGTAATTAATTATTTACTAAAAAGTTCTTCAGTGACT

>Podel.CUFF.1650.1

ATGGGTTTGACAAATATGTCAGATCTAAGCCGCTTAAACTTAGCAGTCAGCTACGTACAAAACACTTGGACTTAG

>Podel.CUFF.2373.1

ATGAATACCCTTGGGTGCGCAGAATTAGAAACGGTGCAGGCGAAGTTGCGCAGGCTAAGGGTCTGCATGACCTAG

>Podel.CUFF.279.1

ATGAATAAAGCAGGTACTTATGATAGCTTGTCATATCTAGATGTTCTCCTGGTGCAGCATATATTGATGTTGTGCTGA

>Podel.17G101000.1.v2.1

ATGGAGGAATTGCTCGCTTCCTCATGGGAGAATCCCTTTGCTAGAGTTTTAGCTAAAATAACTAGAAGAAAGCTATTTTAG

>Podel.CUFF.142.1

ATGGAATTTAGGAGTGATTCTTGGAAGTTGTGGAATCTAGCATCTCGGAGTATATCTAGAATGGAGGTATTTAAGCCTTCTTTTGTAAGTAGTTTTATTTCGACTTGA

>Podel.CUFF.2820.1

ATGAGCTTGCATGCGTTGATCAGTTCTTGGTATTTCTGTTTGGCCTTGCATGTTGAGGGCTGGACGTGCATGGTTCCTCGTTTCTGCATGGTCTTTCATTTAATTTGGCTGGGTGGGAGCTTCTGA

>Podel.CUFF.2100.1

ATGTTATATATGTATGTCAGGAAACTAATCATACAGAACCTAATATTATGGGATGATTATGTCAGGAGTAGGAGTTCTAATAGTTATGATGATAGAGCTAAATTGTTATGGAGATTAGATTGGGCTCAAAAGCTTGTACCTCTAGCACATTAA

>Podel.CUFF.139.1

ATGCTGTTTGAGATCAACCTTCATTTGAATAGGTCTTGCCTTTATAGGTATATGTTTTTTCGGAAAAAGTATGTTCATAAGCAAGATCAGCAACATGTTGTTTTCTTTTCCAGAAGGCATTGGGTAAGTCTGAACAGATTTGTTGTTCAATCTTACTTTGTAAATCAGTGA

>Podel.10G150900.1.v2.1

ATGAGGGAAAGAGAGAAGGGTCTTTCAAAGGTGAAAGAAAGGCACAAGTTTCTGCAAGGTAACCTGTATAAGGGTATGAACAAAGCCATCATGTGTTACACTACAAGCCAGGAAGGAAGCCTAGTAGATGGATTCTTTGCTGGCTTTCAAAAGGCGGTTTCTTCATGTTAA

>Podel.05G157600.1.v2.1

ATGGGTTTGGAACAGATCAGTCTGTCTGGAAGCACTTGGTTCCCCACCTTGTTGATGAGTACACTGTTATTTTGTATGATAACATGGGAGCTGGTACTACAAATCCAGATTACTTTGATTTCAGTAGGTACTCTACCCTCGAAGGTTTTGCTTATGATTTACTTGCCATTTTAG

>Podel.08G078000.1.v2.1

ATGAAATCCGAACTTAGCCAACAGTTTGATGAAAATACCACGCTAATTACCAATGGGCCTATTGGTTCCAGTCGTAGGTTACTCTTGCAGGCGAGAGGTGCTGGGTTCAACTTGGGGGTGGAGTTGAACCTTACCTTCCCGACAACCCGAAATTGTTCTAGTTTCGGGTTGGAGAGCACTGAGAGTTCTTGA

>Podel.CUFF.3113.1

ATGTCAACCATAAGAAGTGCCATTCAGGGGCCGTGTCGGGGTGGAAACAATGCTGATGGAAGAATGAGGCGTGTCTCTCTTGGTATCCTTGTTTATATTATCTGGGAGGAGAGGAACAAAAGAATTTTTTACAGCACATGCAGCTCTATTGCTTCTCTCTTTCGCAAATTCCAGACTCTATTTTTCATGGTTTTCCATTTCCACGACAATGATCATTTTTCTCTCCATGTTGGCTGCTGA

**Coding sequences of small open-reading-frames (sORFs) in *Populus trichocarpa***

>Potri.CUFF.1343.1

ATGTTAGTTCTCAACATTATAATCATTGATTCTTTGAATTAA

>Potri.CUFF.184.1

ATGAGCAGATGTATATTGGAGGATTTGTTCTTTGTTGTTTCGGACTTGTTTAAGAATCCCTCGGTAACCCAAGTCTGA

>Potri.CUFF.347.1

ATGATAACTAGAGCTCAATGTGAGTTCTATCGGGTAAAGACAATGATTAAAGGCATCGAGGGCATAACTCCCTTAACCTATTCTCAAACTTTAAATATGTAG

>Potri.005G145000.1.v3.1-sense

ATGACTGTAACGTCCATTGTGGTTTGGCTGTGTCAAGTTGTGATTACTCATCATGTAATAAGATTCAAGAGAACATGGAGTTCGAATAGGCCTTTTTTCAAACAAAGAAAGCGAAAGAAGTTCGGTTCTTGCTTGCAAACTACATAA

>Potri.007G077200.1.v3.1

ATGCAGCAGAAGCTCTCAAGCCTTGGGCGAAAGGCGAAAGCTCTTAGGCGGCCACTGCGAATGTTCTTGAGAAACAAGCCTTTGGATCTATCATCATTGACCTCTCTAACATCATCATCCATTCATTAG

>Potri.013G036500.1.v3.1

ATGATGGTAGGAGCATGGCCACCAAGCACAAGAAGAATATTTGCGTTGTGCGTAAAAGTAGTAATGAGAGCTACTCATGTTTTAGCGCAGAAAGTCTTTCATCACCTTGATAACAAAAAGGAACCAACCTCCTTGAAATTTTCAGTTCTTGTTTGGAGTGAGTGTCTGGGAATCCTAGGTGCTGTGGTGGTGGAGATGGCGGTGGTGCATGCGCGCATGGTTTCAATATTTCCTTGCTATTCAGTTGCTGGTTCATGTGGGGCTCGTGAATAG
