# Supplementary material for: Identification of Populus Small RNAs Responsive to Mutualistic Interactions With Mycorrhizal Fungi, Laccaria bicolor and Rhizophagus irregularis
Source: Front Microbiol. 2019 Mar 18;10:515. doi: 10.3389/fmicb.2019.00515 (PMC6431645; doi:10.3389/fmicb.2019.00515)
Supplement: Supplementary file 6 [file Table_6.DOCX]

Protein sequences of small open-reading-frames (sORFs) in *Populus deltoids*

>Podel.CUFF.196.1

MDFEINNHVNFQ

>Podel.CUFF.3016.1-sense

MYPKQKSYLRFF

>Podel.CUFF.830.1

MVPWSSGEDIGL

>Podel.CUFF.1615.1

MLLPRIELGTFSV

>Podel.CUFF.1796.1

MMVICFIHIRSTFE

>Podel.CUFF.2933.1

MVPRVEQRADATPG

>Podel.CUFF.2671.1

MQVSNLRLRVISTTL

>Podel.CUFF.1658.1

MVINFRIHEISRVMPKLT

>Podel.07G087100.1.v2.1

MMMIDPKACFSRTFAVAA

>Podel.CUFF.2992.1

MVMKDCVDKSIVINYLLKSSSVT

>Podel.CUFF.1650.1

MGLTNMSDLSRLNLAVSYVQNTWT

>Podel.CUFF.2373.1

MNTLGCAELETVQAKLRRLRVCMT

>Podel.CUFF.279.1

MNKAGTYDSLSYLDVLLVQHILMLC

>Podel.17G101000.1.v2.1

MEELLASSWENPFARVLAKITRRKLF

>Podel.CUFF.142.1

MEFRSDSWKLWNLASRSISRMEVFKPSFVSSFIST

>Podel.CUFF.2820.1

MSLHALISSWYFCLALHVEGWTCMVPRFCMVFHLIWLGGSF

>Podel.CUFF.2100.1

MLYMYVRKLIIQNLILWDDYVRSRSSNSYDDRAKLLWRLDWAQKLVPLAH

>Podel.CUFF.139.1

MLFEINLHLNRSCLYRYMFFRKKYVHKQDQQHVVFFSRRHWVSLNRFVVQSYFVNQ

>Podel.10G150900.1.v2.1

MREREKGLSKVKERHKFLQGNLYKGMNKAIMCYTTSQEGSLVDGFFAGFQKAVSSC

>Podel.05G157600.1.v2.1

MGLEQISLSGSTWFPTLLMSTLLFCMITWELVLQIQITLISVGTLPSKVLLMIYLPF

>Podel.08G078000.1.v2.1

MKSELSQQFDENTTLITNGPIGSSRRLLLQARGAGFNLGVELNLTFPTTRNCSSFGLESTESS

>Podel.CUFF.3113.1

MSTIRSAIQGPCRGGNNADGRMRRVSLGILVYIIWEERNKRIFYSTCSSIASLFRKFQTLFFMVFHFHDNDHFSLHVGC

Protein sequences of small open-reading-frames (sORFs) in *Populus trichocarpa*

>Potri.CUFF.1343.1

MLVLNIIIIDSLN

>Potri.CUFF.184.1

MSRCILEDLFFVVSDLFKNPSVTQV

>Potri.CUFF.347.1

MITRAQCEFYRVKTMIKGIEGITPLTYSQTLNM

>Potri.005G145000.1.v3.1-sense

MTVTSIVVWLCQVVITHHVIRFKRTWSSNRPFFKQRKRKKFGSCLQTT

>Potri.007G077200.1.v3.1

MDDDVREVNDDRSKGLFLKNIRSGRLRAFAFRPRLESFCCIFPSLDSLIQFCDYFANLHNDPAA

>Potri.013G036500.1.v3.1

MMVGAWPPSTRRIFALCVKVVMRATHVLAQKVFHHLDNKKEPTSLKFSVLVWSECLGILGAVVVEMAVVHARMVSIFPCYSVAGSCGARE
